# In Silico Approach Using Free Software to Optimize the Antiproliferative Activity and Predict the Potential Mechanism of Action of Pyrrolizine-Based Schiff Bases

**DOI:** 10.3390/molecules26134002

**Published:** 2021-06-30

**Authors:** Faisal A. Almalki, Ashraf N. Abdalla, Ahmed M. Shawky, Mahmoud A. El Hassab, Ahmed M. Gouda

**Affiliations:** 1 Department of Pharmaceutical Chemistry, Faculty of Pharmacy, Umm Al-Qura University, Makkah 21955, Saudi Arabia; famalki@uqu.edu.sa; 2 Department of Pharmacology and Toxicology, Faculty of Pharmacy, Umm Al-Qura University, Makkah 21955, Saudi Arabia; anabdrabo@uqu.edu.sa; 3 Department of Pharmacology and Toxicology, Medicinal and Aromatic Plants Research Institute, National Center for Research, Khartoum 2404, Sudan; 4 Science and Technology Unit (STU), Umm Al-Qura University, Makkah 21955, Saudi Arabia; amesmail@uqu.edu.sa; 5 Central Laboratory for Micro-Analysis, Minia University, Minia 61519, Egypt; 6 Department of Pharmaceutical Chemistry, School of Pharmacy, Badr University in Cairo (BUC), Cairo 11829, Egypt; mahmoud65582@pharm.tanta.edu.eg; 7 Medicinal Chemistry Department, Faculty of Pharmacy, Beni-Suef University, Beni-Suef 62514, Egypt

**Keywords:** pyrrolizine, antiproliferative, cell cycle analysis, apoptosis, pharmacophore search, docking study, MD simulation

## Abstract

In the current study, a simple in silico approach using free software was used with the experimental studies to optimize the antiproliferative activity and predict the potential mechanism of action of pyrrolizine-based Schiff bases. A compound library of 288 Schiff bases was designed based on compound **10,** and a pharmacophore search was performed. Structural analysis of the top scoring hits and a docking study were used to select the best derivatives for the synthesis. Chemical synthesis and structural elucidation of compounds **16a–h** were discussed. The antiproliferative activity of **16a**–**h** was evaluated against three cancer (MCF7, A2780 and HT29, IC_50_ = 0.01–40.50 μM) and one normal MRC5 (IC_50_ = 1.27–24.06 μM) cell lines using the MTT assay. The results revealed the highest antiproliferative activity against MCF7 cells for **16g** (IC_50_ = 0.01 μM) with an exceptionally high selectivity index of (SI = 578). Cell cycle analysis of MCF7 cells treated with compound **16g** revealed a cell cycle arrest at the G_2_/M phase. In addition, compound **16g** induced a dose-dependent increase in apoptotic events in MCF7 cells compared to the control. In silico target prediction of compound **16g** showed six potential targets that could mediate these activities. Molecular docking analysis of compound **16g** revealed high binding affinities toward COX-2, MAP P38α, EGFR, and CDK2. The results of the MD simulation revealed low RMSD values and high negative binding free energies for the two complexes formed between compound **16g** with EGFR, and CDK2, while COX-2 was in the third order. These results highlighted a great potentiality for **16g** to inhibit both CDK2 and EGFR. Taken together, the results mentioned above highlighted compound **16g** as a potential anticancer agent.

## 1. Introduction

Cancer is still one of the leading causes of death in the world [1]. Among females, breast cancer is the most common type of cancers, whereas, in males, colon cancer was ranked third in the incidence in the USA in 2021. Due to the consistently high rates of cancer incidence and mortality, research in this field is always in demand and of continuous interest.

**Figure 1:**
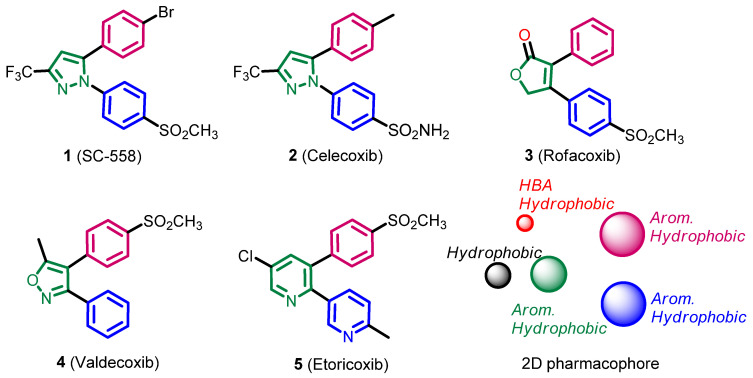
Coxibs **1–5** and a 2D pharmacophore generated based on their chemical structures.

Before thirty years, the antiproliferative effect of nonsteroidal anti-inflammatory drugs (NSAIDs) was discovered [2]. Since then, there has been much greater interest from many researchers around the world, hoping to explore the antiproliferative potential of NSAIDs and investigate their mechanisms of action. The selective COX-2 inhibitors, coxibs **1**–**5** (Figure 1), attracted much attention in cancer research, although most of them were removed from the market [3]. Considering their chemical structures, we can observe that compounds **1–5** have similar pharmacophoric features which include three aromatic rings and hydrophobic atom/group attached to the five-membered ring. The aryl rings in these compounds are either unsubstituted or substituted at the *para*-position with hydrophobic groups (CH_3_/Br) or hydrogen bond-forming groups (SO_2_CH_3_/SO_2_NH_2_) which occupy the side pocket in COX-2.

Among these drugs, celecoxib **2** was extensively studied for its antiproliferative, either alone or in combination with anticancer drugs [4,5,6,7,8]. Celecoxib **2** showed antiproliferative activity against breast and colon cancer cells [4,5,6]. It also showed a chemopreventive effect and was approved by the FDA for familial adenomatous polyposis (FAP) [6]. Moreover, Celecoxib **2** exhibited anticancer and antimetastatic effects against several types of ovarian cancer cells [7]. 

Substantial research was also conducted to investigate the mechanism of action of NSAIDs [2,8,9,10,11]. Based on these studies, the anticancer activity of NSAIDs can be attributed to the COX-dependent mechanisms [2]. However, COX-independent mechanisms were also reported to mediate the anticancer potential of celecoxib **2** [8,9,10,11]. The induction of apoptosis in HT29 cells by celecoxib **2** was attributed to the inhibition of 3-phosphoinositide-dependent protein kinase-1 [9]. The inhibition of Akt kinase activation could also play an important role in the induction of apoptosis in prostate cancer cells [10]. Mechanistic studies also showed that celecoxib **2** blocks the activation of MAP p38 kinase and downregulates COX-2 [11]. 

**Figure 2:**
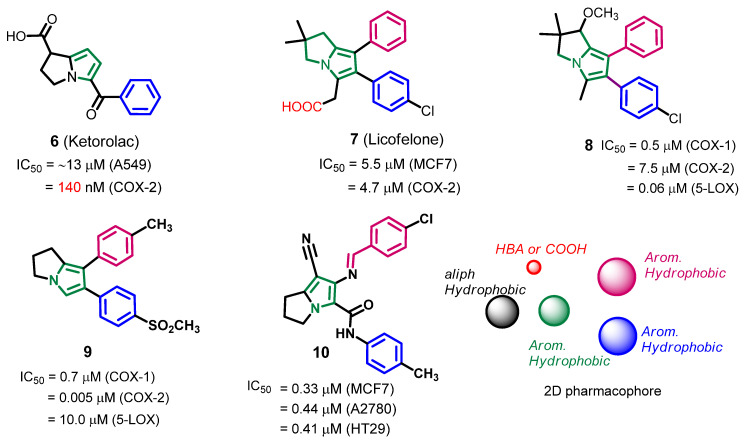
Pyrrolizines **6**–**10** with anti-inflammatory and/or anticancer activities and a 2D pharmacophore generated based on their chemical structures.

To date, celecoxib is still the only NSAID that has been approved for FAP [6]. This may be attributed to the weak anticancer activities of NSAIDs or to their toxic side effects of these drugs [2,12]. According to this, extensive research in this field should be conducted to develop new scaffolds with potent anticancer activities and good safety profiles. Among the promising scaffolds, pyrrolizine was found in many compounds **6–10** (Figure 2) with potent anti-inflammatory and cytotoxic activities [13,14,15,16,17,18,19,20]. Among these pyrrolizines, ketorolac **6** exhibited potent analgesic and anti-inflammatory activity [13]. In addition, ketorolac **6** showed anticancer activity against A549 cells (IC_50_ = ~13 µM) [14]. This weak anticancer activity of ketorolac was attributed to the carboxylic acid group which hinders its permeability into the cells [14]. 

Licofelone **7** (Figure 2) also displayed a strong anti-inflammatory activity mediated by inhibition of COX and 5-LOX enzymes [15,16]. Compounds **8**–**9** are also pyrrolizine derivatives which lack the carboxylic acid group of licofelone; however, they also exhibited inhibitory activities against COXs [15]. Biological evaluation of licofelone **7** also revealed cytotoxic activities against breast, colon, and prostate cancer cell lines [17,18,19]. In addition, compound **10** showed moderate anti-inflammatory activity compared to ibuprofen and cytotoxic activity against three cancer cell lines [20]. Mechanistic studies of compound **10** revealed weak inhibitory activity against COX-2. 

Investigation of the chemical structure of compounds **7**–**10** also revealed similar pharmacophoric features which include a central five-membered pyrrole ring attached to two (un)substituted phenyl rings. These features are also similar to those compounds **1**–**5** (Figure 1). However, compound **10,** which lacks the acidic group, exhibited higher antiproliferative activity compared to both celecoxib **2** and licofelone **7** [6,17]. 

The high antiproliferative activity of compound **10** may deserve further investigation and optimization. Accordingly, the current study aimed to optimize the antiproliferative activity of compound **10**. This aim will be achieved by designing a series of new analogs and evaluate their antiproliferative activities. The design of the new analogs will use different techniques of computer-aided drug design [21,22,23], which proved successful in optimization of lead compounds, will be used to in the optimization of compound **10**.

## 2. Results and Discussion 

### 2.1. Pharmacophore Search

#### 2.1.1. Design of the Compound Library

**Figure 3:**
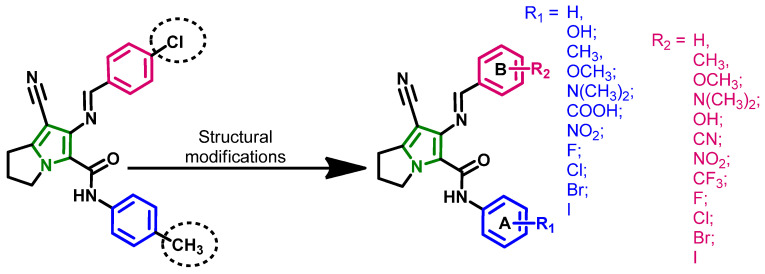
Structural modifications of compound **10**.

To optimize the antiproliferative activity of compound **10**, a small compound library of pyrrolizine-based Schiff bases was designed. The new analogs were obtained by modification of the chemical structure of compound **10**. In this study, we will focus mainly on variation of the substituents on the two phenyl rings (A and B), Figure 3.

The compound library included 288 Schiff bases designed bearing electron-donating/withdrawing substituents at the *ortho/meta*/*para-*positions of the two phenyl rings. The chemical structures of these compound library are provided in Supplementary Data (Figures S52–S61).

#### 2.1.2. Pharmacophore Search 

To identify the new analogs with the highest potential inhibitory activity against COX-2, pharmacophore search of the compound library was performed using Pharmit (http://pharmit.csb.pitt.edu) [24]. This software package is available online and provides a free tool to perform pharmacophore/shape search of the large compound libraries (up to 10 million compounds). In an attempt to build a valid pharmacophore to screen our compound library, three of the crystal structures of COX-2 bound to the selective inhibitors SC-558 **1** (pdb: 1CX2) [25], celecoxib **2** (pdb: 3LN1) [26], and rofecoxib **3** (pdb: 5KIR) [27] were analyzed to identify different types of binding interactions. Analysis of the binding interactions was done using Discovery Studio Visualizer (DSV) [28]. Among the three compounds **1**–**3**, celecoxib **2** displayed the highest number of hydrogen bonds (Supplementary Data, Figure S1). However, rofecoxib **3** exhibited only one conventional hydrogen bond with Arg513 and displayed higher inhibitory activity and selectivity against COX-2 (IC_50_ = 0.53 μM, SR = 35.5) compared to celecoxib **2** [29]. In addition, SC-558 **1** also showed higher inhibitory activity and selectivity toward COX-2 compared to celecoxib [25], although it formed a fewer number of hydrogen bonds. Moreover, analysis of the binding interactions of compounds **1**–**3** revealed multiple hydrophobic interactions with the amino acids in COX-2.

**Figure 4:**
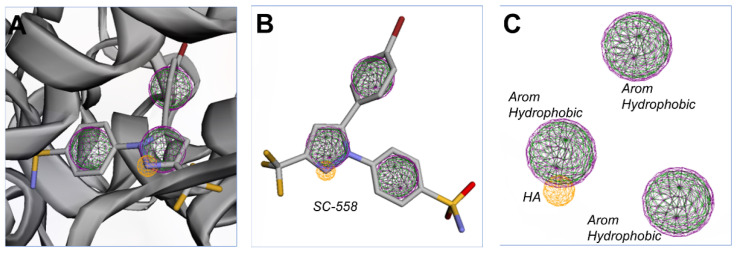
Pharmacophore model A of SC-558 (shown as sticks) into COX-2 (a potential target of the designed compound): (**A**) 3D binding mode of SC-558 into COX-2; (**B**) SC-558; (**C**) pharmacophoric features of SC-558.

Based on the above results, a simple pharmacophore model A (Figure 4) was generated based on the binding interactions of SC-558 with COX-2 (pdb: 1CX2). This model consists of seven pharmacophoric features including three aromatic rings, hydrophobic groups and one hydrogen bond acceptor. 

**Figure 5:**
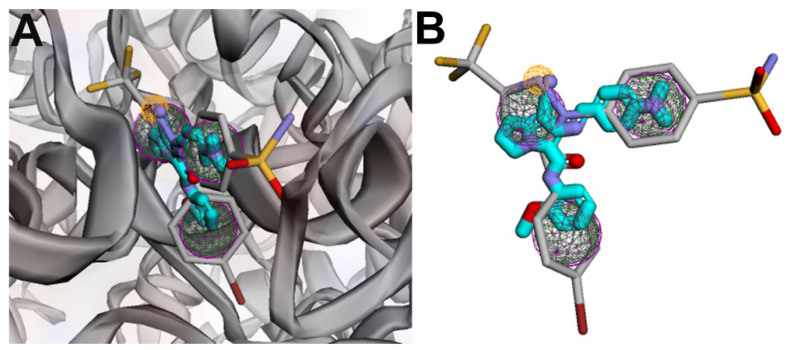
The binding site of SC-558 (shown as sticks colored by element) into COX-2: (**A**) 3D binding mode of SC-558 into COX-2 overlaid with hit1 (cyan sticks); (**B**) 3D overlay of SC-558 overlaid with hit1 (cyan sticks).

To perform the pharmacophore search, the compound library was first uploaded to the Pharmit server. The pharmacophore search was performed using the pharmacophore model A, Figure 4. The active site of COX-2 enzyme where each derivative of the compound library was located overlaid with SC-558 was illustrated in Figure 5. 

The Pharmacophore search was performed, and the results in the form of hits ranked based on their root-mean-square deviation (RMSD) were presented in Table 1. The molecular weight (MWs) and the number of rotatable bonds (RBs) in each hit were also calculated. 

**Figure 6:**
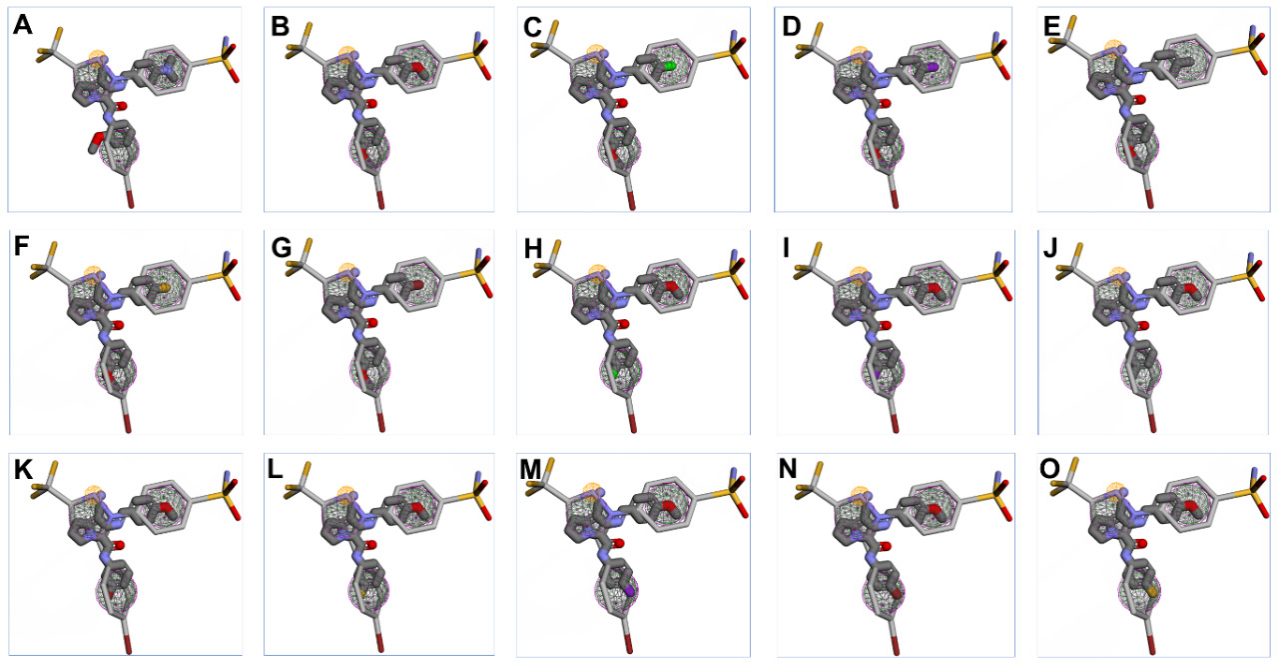
The top fifteen hits **1**–**15** (shown as sticks) overlaid with SC-558 (shown as sticks): (**A**) hit **1**; (**B**) hit **2**; (**C**) hit **3**; (**D**) hit **4**; (**E**) hit **5**; (**F**) hit **6**; (**G**) hit **7**; (**H**) hit **8**; (**I**) hit **9**; (**J**) hit **10**; (**K**) hit **11**; (**L**) hit **12**; (**M**) hit **13**; (**N**) hit **14**; (**O**) hit **15**.

Analysis of the results of the pharmacophore search revealed that the top seven scoring hits **1**–**7** bear the methoxy group on the phenyl ring A, Table 1. Of these derivatives, hit **1** with the methoxy group on ring A and dimethylamino group at *para*-position of the ring (B) exhibited the lowest RMSD. The chemical structure of the top fifteen hits **1**–**15**, overlaid with SC-558 were arranged based on their RMSD values as illustrated in Figure 6. 

**Figure 7:**
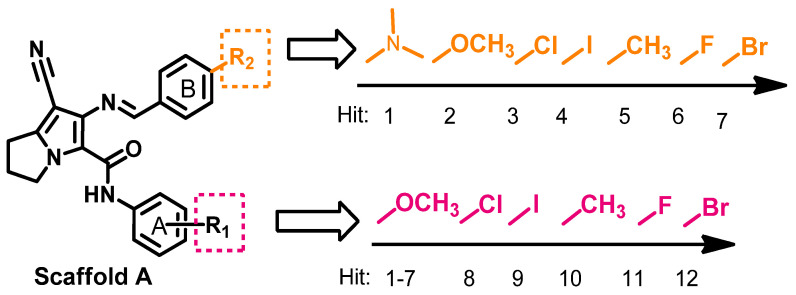
The order of substituents on the phenyl rings of the top fifteen hits.

The results of the pharmacophore search were analyzed to identify the substituents on the phenyl rings of the top-scoring hits. The results revealed that the order of substituents on the phenyl ring (A) in the following sequence: OCH_3_ (in the top 7 Hits), Cl, I, CH_3_, F, and Br. On the other hand, the substituents of phenyl ring (B) were in the following order: N(CH_3_)_2_, OCH_3_, Cl, I, CH_3_, F, and Br, Figure 7.

Although hit **4**, hit **9**, and hit **13** have displayed low RMSD values (Table 1), they have high molecular weights (>500 daltons). Considering the limits of Lipinski’s rule, the three hits will be excluded from the compounds selected for the chemical synthesis. 

#### 2.1.3. Compounds Selected for the Synthesis

**Figure 8:**
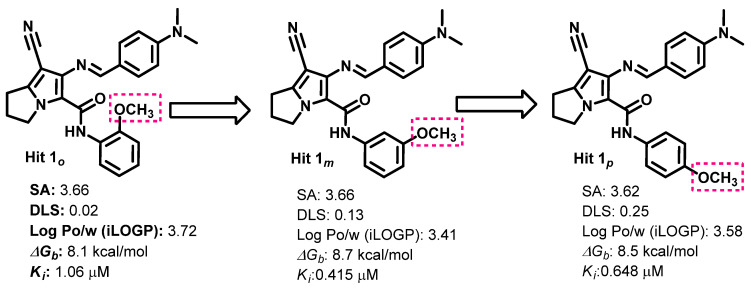
Effect of changing the position of OCH_3_ group on SA, DLS, ClogP values, binding affinities (Δ*G_b_*), and inhibition constants (K_i_) of hits **1***_o,m,p_*.

Based on the above results of the pharmacophore search, hit **1** was selected for the next step in this study. The three isomers of hit **1** (Figure 8) were evaluated for their binding affinities toward COX-2 in a preliminary docking study. The study was to evaluate the impact of the position of the methoxy group on the binding affinities toward COX-2. The results of this study are provided in the Supplementary Data (Figure S2). The results revealed higher binding affinities for hits **1*_m,p_*** than hit **1*_o_*** which has a methoxy group in *ortho*-position. Investigation of the binding interactions of the three derivatives revealed that hit **1*_o_*** lacks any conventional hydrogen bonds with COX-2, while hits **1*_m,p_*** showed two conventional hydrogen bonds each. 

In addition, logP (ilogP) and synthetic accessibility (SA) of the three hits **1***_o,m,p_* were calculated using SwissADME (http://www.swissadme.ch) [30], while their drug-likeness scores (DLSs) were calculated by Molsoft (http://molsoft.com/mprop/) [31]. The results are presented in Figure 8. The results revealed the lowest SA and highest DLS for hit **1*_p_***. These results are also matched with the substitution pattern of compounds **1–10**, which include phenyl groups substituted at the *para*-position (Figure 1 and Figure 2). Accordingly, hit **1*_p_*** was used as the first target compound to be synthesized in this study. 

**Figure 9:**
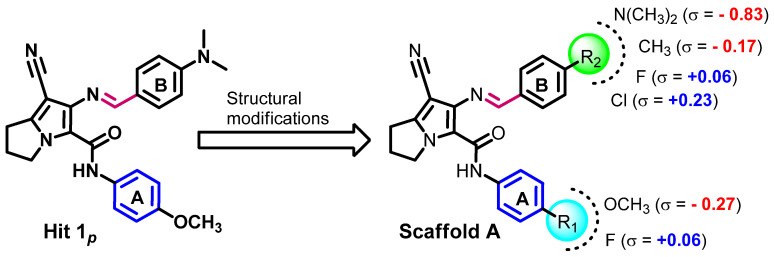
Structural modifications of hits **1***_p_*.

To study the SAR of hit **1*_p_***, the electron-donating (methoxy) group was replaced with an electron-withdrawing flour–atom to investigate the impact of the electronic effect of substituents on their antiproliferative activities, Figure 9. In addition, four other substituents including two electron-donating (-N(CH_3_)_2_ and -CH_3_) and two electron-withdrawing (-F and -Cl) atoms. These substituents were selected based on the results of the pharmacophore search, Figure 7. 

### 2.2. Synthesis of the New Derivatives

**Scheme 1:**
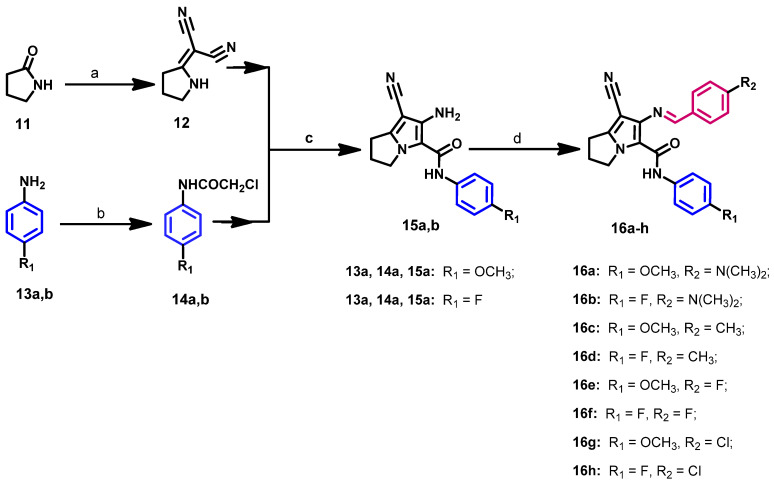
Reagents and reaction conditions (**a**) (CH_3_)_2_SO_4_, benzene, CH_2_(CN)_2_; (**b**) ClCH_2_COCl, glacial AcOH, CH_3_COONa; (**c**) acetone, K_2_CO_3_, reflux, 24 h; (**d**) appropriate aldehyde, EtOH, AcOH, reflux, 4–6 h.

In this section, the starting materials **12**, **14a**,**b**, and **15a**,**b** (Scheme 1) were prepared according to the previously reported procedures [32,33,34]. In addition, the target Schiff bases **16a**–**h** were prepared by refluxing compounds **15a**,**b** with the appropriate aldehydes following the previous report [20]. 

The IR spectra of **16a**–**h** revealed an absorption band at the range of 2209–2214 cm^−1^ indicating the cyano groups. In addition, an absorption band was also observed at the range of 1656–1669 cm^−1^ indicating the carbonyl groups in **16a**–**h**.

The proton magnetic spectrum of compound **16a**,**b** revealed a singlet signal at 3.13 ppm indicating the six protons of the N(CH_3_)_2_ group. Four doublets at the range of *δ* 6.79–7.82 ppm indicating the *para*-substituted phenyl rings in compound **16a**, while the aromatic protons of **16b** was observed at the range of *δ* 6.86–7.95. Two singlet signals at the range of *δ* 8.77–11.00 ppm indicating the benzylidene (N=CH) and amide (CONH) protons of **16a**,**b**.

The ^13^C-NMR also revealed a signal at *δ* 40.27 and 40.67 ppm indicating the carbon atoms of N(CH_3_)_2_ group in **16a** and **16b**, respectively. DEPT C^135^ spectrum showed a signal at *δ* 159.59 and 158.43 ppm indicating benzylidene carbon (N=CH) in **16a** and **16b**, respectively. 

The ^13^C-NMR and DEPT C^135^ spectra of compounds **16b**,**d**,**f**,**g** which include 4-flourophenyl moiety, revealed splitting of the four carbon signals of the aromatic ring due to the coupling of carbon with the fluorine atom. 

Mass spectra of the **16a**–**h** revealed the molecular ions at m/z (%) 427 (M^+^, 28), 415 (M^+^, 17), 399 ([M+1]^+^, 13), 386 (M^+^, 51), 402 (M^+^, 28), 391 ([M+1]^+^, 27), 418 (M^+^, 21), and at 406 (M^+^, 8).

Copies of the spectra data including IR, mass, ^1^H-NMR, ^13^C-NMR, and DEPT C^135^ spectra of compounds **16a**–**h** are provided in Supplementary Data (Figures S3–S45). 

### 2.3. Biological Evaluation of the New Compounds

#### 2.3.1. Antiproliferative Activity

##### Antiproliferative Activity Assay

The antiproliferative activities of the eight Schiff bases **16a**–**h** against three cancer cell lines (MCF-7, A2780, and HT29) were evaluated using the MTT assay. The selection of these cell lines was done to compare the antiproliferative activities of the new compounds with those of compound **10** [20]. The assay was performed following the previous report [35]. These results of the MTT assay are presented in Table 2. 

Compounds **16a**–**h** exhibited their antiproliferative activity at IC_50_ values in the range of 0.01–40.50 μM against the three cancer cell lines compared to compound **10** (IC_50_ = 0.33–0.44 μM). These results also indicated high antiproliferative activity for compounds **16a**–**h** compared to lapatinib (IC_50_ = 5.98–13.22 μM).

In addition, compounds **16b**,**c**, **16e**, and **16g** showed higher activity against MCF7 cells than compound **10**. Moreover, higher antiproliferative activities were observed for compounds **16a**, **16d**–**g** against HT29 cells compared to compound **10**. However, only compound **16c** was more active against A2780 cells than compound **10**. Among the new derivatives, compound **16g** was the most active against MCF7 cells, while **16c** and **16d** showed the highest antiproliferative activities against A2780 and HT29 cells, respectively, Table 2.

##### Evaluation of Antiproliferative Selectivity

Selectivity of the antiproliferative agents toward cancer cells plays a critical role in the development of these agents as potential anticancer drugs. Although the lead compound **10** exhibited high antiproliferative activity, its toxicity and selectivity were not evaluated [20]. In the current study, all of the new compounds **16a**–**h** were evaluated for the antiproliferative activities against the normal MRC5 cells. The cells were treated compounds for 72 h, and the IC_50_ values were calculated in Table 3. The aim of this study was to assess the toxicity of the new compounds against normal MRC5 cells. In addition, the IC_50_ values were also used to calculate the selectivity index (SI) which measures the selective cytotoxicity of the new compounds.

The results showed that compounds **16a**–**h** exhibited their antiproliferative activities against MRC5 cells at IC_50_ values in the range of 1.27–24.06 μM compared to lapatinib (IC_50_ = 14.89 μM). In addition, the new compounds exhibited SIs in the range of 0.03–802 toward the three cancer cell lines compared to MRC5 cells. Compounds **16b**,**c**,**e**,**g** showed SIs > 10 toward MCF7 cells, while **16c**,**d**,**g** were selective to HT29 cells (SIs >10). On the other hand, only two compounds (**16c**,**g**) showed more than 10-fold higher selectivity toward A2780 cells compared to MRC5 cells, Table 2.

Among these new compounds, **16c** showed the least toxicity toward MRC5 cells. Moreover, **16c** showed the highest SI against MCF7 and A2780 cells, while **16g** was the most selective to HT29 cell line. The results also revealed an SI in the range of 10.32–578.00 for compound **16g** against the three cancer cell lines compared to MRC5 cells, Table 2.

##### Structure–Activity Relationship (SAR)

**Figure 10:**
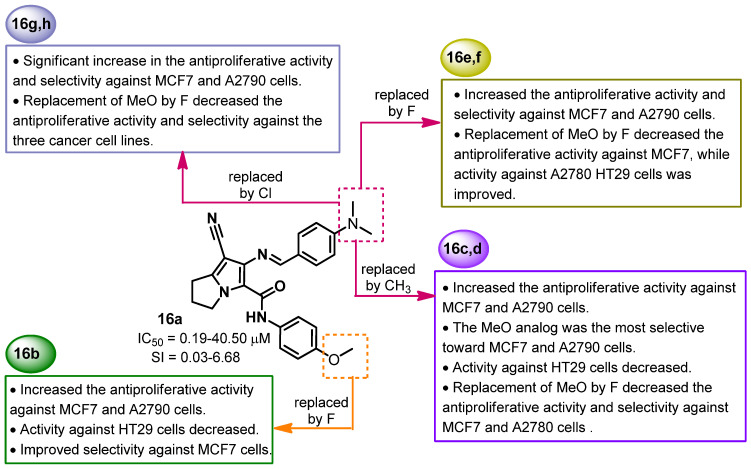
SAR of the antiproliferative activity/selectivity of compounds **16a**–**h**.

In an attempt to illustrate the impact of different substituents on the antiproliferative activity and selectivity of the new compounds **16a**–**h**, the study of SAR of these compounds was illustrated in Figure 10. To evaluate the effect of different substituents on activity/selectivity, we started with compound **16a** which exhibited IC_50_ values in the range of 0.19–40.50 μM against the tested cancer cell lines, and SI in the range of 0.03–6.68. We found that the replacement of the methoxy group in **16a** by fluoro atom caused a significant increase in the antiproliferative activities against MCF7 and A2790 cells, while activity against HT29 cells was decreased. An improvement in the selectivity of compound **16b** toward MCF7 cells compared to **16a** was also observed, Figure 10.

Compound **16c** also showed higher antiproliferative activity against MCF7 and A2790 cells compared to **16a**. Among the new compounds, compound **16c** was the most selective toward MCF7 and A2790 cells. However, a sharp decrease in antiproliferative activity and selectivity toward MCF7 and A2780 cells was observed upon replacement of the methoxy group in **16c** by fluoro atoms, Figure 10.

Meanwhile, the replacement of the dimethylamino group in **16a** by fluoro resulted in an increase in the antiproliferative activity and selectivity toward MCF7 and A2790 cells. However, the replacement of the methoxy group in **16e** by F atom decreased the antiproliferative activity against MCF7, while the activities against A2780 HT29 cells were slightly improved. Similarly, the replacement of the dimethylamino group in **16a** by chloro also increased the antiproliferative activity and selectivity toward MCF7 and A2790 cells. On the other hand, replacement of methoxy group in **16g** by F also decreased antiproliferative activity and selectivity against the three cancer cell lines.

In conclusion, the antiproliferative activity and selectivity of compounds **16c**,**e**,**g** toward MCF7 cells were decreased upon replacement of methoxy group by the flour atom. These results suggested that the methoxy analogs are more favored for high antiproliferative activity and selectivity.

#### 2.3.2. Determination of Cell Cycle Perturbations

**Figure 11:**
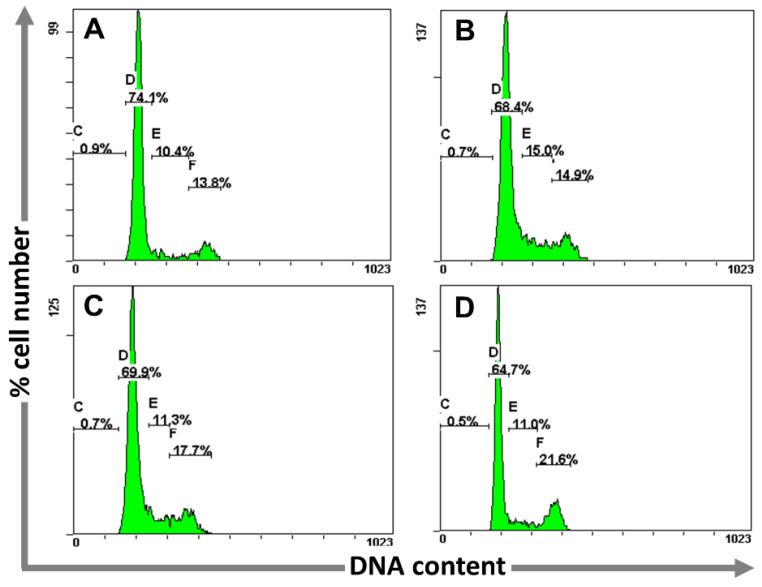
Flow cytometry histograms showing the effect of compound **16g** on cell cycle distribution after 72 h treatment in MCF-7 cells. *X*-axis: DNA content of 20,000 events, y axis: % cell number. (**A**); 0 μM; (**B**): 0.01 μM; (**C**): 0.05 μM; (**D**): 0.10 μM. (*n* = 3).

Cell cycle analysis of MCF7 cells was also performed to investigate the mechanism which mediates the antiproliferative activity of the new compounds. Compound **16g,** the most active in MTT assay (Table 2), was selected for this assay. The assay was performed according to the previous report [36]. The results are represented in Figure 11.

**Figure 12:**
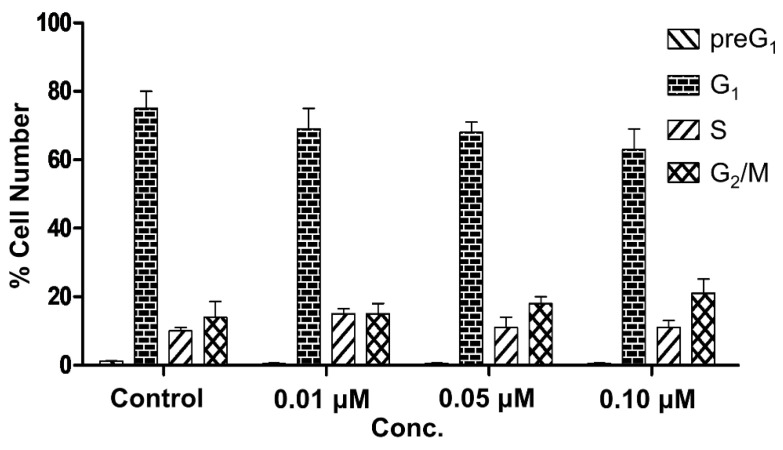
A bar chart showing the effects of compound **16g** on cell cycle distribution after 72 h treatment of MCF-7 cells with 0, 0.01, 0.05, and 0.10 μM. (*n* = 3).

The results revealed cell cycle arrest at the G_2_/M phase by **16g**. The increase in the number of MCF7 cells in the G_2_/M phase was associated with a dose-dependent decrease in the number of cells in the G_1_ phase, Figure 12.

#### 2.3.3. Annexin V FITC/PI Apoptosis Assay

**Figure 13:**
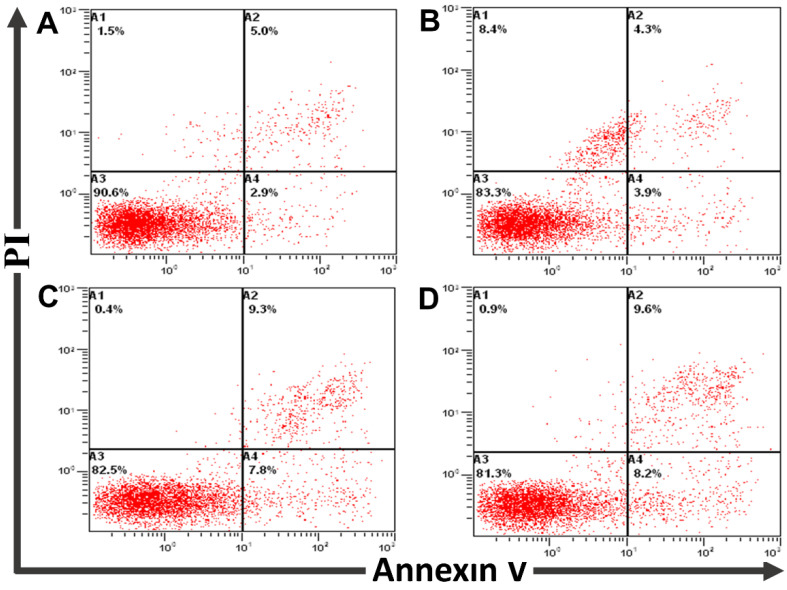
Detection of early/late apoptosis in MCF7 cells treated with **16g** using annexin V FITC/PI staining assay for 72 h (*n* = 3), *x*-axis: annexin V, *y*-axis: PI; (**A**); 0 μM; (**B**): 0.01 μM; (**C**): 0.05 μM; (**D**): 0.10 μM. Top left quarter: necrosis (PI+/annexin V−); top right quarter: late apoptosis (PI+/annexin V+); bottom left quarter: living cells (PI-/annexin V−); bottom right: early apoptosis (PI-/annexin V+).

To investigate the ability of compound **16g** to induce apoptosis, MCF7 cells were treated with three different concentrations (0.01, 0.05, and 0.10 μM) of the test compound following the previous report [37]. The results showed that compound **16g** induced a dose-dependent increase in the apoptotic events in MCF7 cells compared to the control group (from 7.9–17.8%), Figure 13.

**Figure 14:**
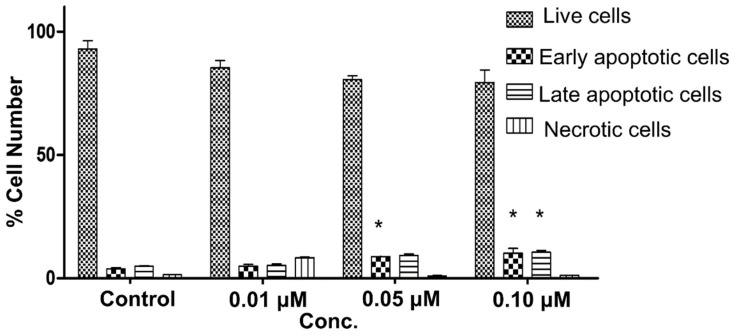
Bar graph showing the effect of compound **16g** on apoptotic events in MCF7 cells (72 h) after staining with annexin V and PI; *x*-axis: concentration, *y*-axis: % cell number. Data shown are % mean ± SD (*n* = 3). The experiment was repeated 3×. after treatment with the test compound at 0, 0.01, 0.05, and 0.10 μM (*n* = 3), statistical differences, compared with control cells, were assessed by one-way ANOVA with the Tukey’s post-hoc multiple comparison test (GraphPad Prism). *p* < 0.05 (*) was taken as significant.

The increase in apoptotic events was associated with a dose-dependent decrease in the number of the living cells, Figure 14. These results also indicated the ability of compound **16g** to induce apoptosis in MCF7 cells at much lower concentration than compound **10** [20].

### 2.4. Target Prediction

In the current study, the new compounds **16a**–**h** exhibited potent antiproliferative activity against one or more of the three cancer cell lines at IC_50_ values much lower than those of NSAIDs [2,38]. These results could be attributed to the ability of the new compounds to act on other targets besides the COX-2 enzyme.

**Figure 15:**
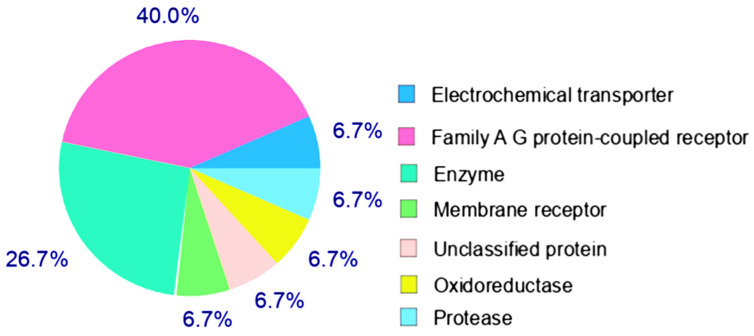
Pie chart showing classification of the potential molecular targets for compound **16g**.

To identify the potential targets which could contribute to the antiproliferative activity of the new compounds, target prediction of compound **16g**, the most active in MTT assay, was evaluated using SwissTargetPrediction [39]. The prediction of the most probable molecular targets by SwissTargetPrediction is done based on 2/3D similarity with a library of 370,000 active compounds. The results in the form of a pie chart showing the top potential targets classified as electrochemical transporters, family A G coupled proteins, enzymes, membrane receptor, oxidoreductase enzyme, protease, and unclassified protein, Figure 15.

In addition, the results of SwissTargetPrediction also include a detailed report showing the potential targets that could mediate the activity of compound **16g**. The report includes the targets arranged in descending order of their probabilities, their common names, and classification. The results of the target prediction of compound **16g** are provided in Supplementary Data (Table S1).

Among 100 entries, the potential targets of compound **16g** were investigated to identify the targets which could contribute to the anti-inflammatory/antiproliferative activities. Among these targets, COX-2, and MAP P38α could contribute to anti-inflammatory and antiproliferative activities of **16g**. In addition, four oncogenic kinases (EGFR, CDK2, BRAF, and VEGFR1) are also among the potential targets that may contribute to the antiproliferative activity of compound **16g**, Table 4.

Similarly, target prediction was also performed for the remaining compounds (**16a-f**, and **16h**). The results of this study were analyzed to identify the shared molecular targets with those of compound **16g**, Table 4.

The roles of COX-2 and MAP P38α in different types of solid tumors were discussed in several reports [2,40]. Accordingly, small molecule inhibitors of these two target proteins exhibited antiproliferative activities against several types of cancer cells [2,40,41]. Moreover, many of the small molecule inhibitors that target EGFR, CDK2, BRAF, and VEGFR1 kinases were also reported with potent anticancer activity [42,43,44,45]. Among these six targets (Table 4), COX-2 and CDK2 were identified as potential targets for all the new compounds, while seven of the new compounds were expected to act on EGFR and VEGFR1. On the other hand, MAP P38α and BRAF were identified as the potential targets for five and four of the new compounds, respectively, Table 4.

### 2.5. Molecular Docking and Binding Mode Analysis

In the current study, the new compounds **16a**–**h** exhibited potent antiproliferative activity against one or more of the three cancer cell lines, Table 2. Considering the weak antiproliferative activity of most of the NSAIDs [2,38], the high potency of the new compounds **16a**–**h** against the tested cancer cell lines could be attributed to their multi-target activity. This conclusion was supported with the results of the target prediction, Table 4. These findings are also in concordance with the findings in our previous report [32]. Accordingly, a molecular docking study of compound **16g** was performed into the molecular targets identified in the target prediction test, Table 4. The aim of this study was to evaluate the binding affinity, orientation, and interactions of compound **16g** against those of the co-crystallized ligands of the six target proteins. The crystal structures of the target proteins were downloaded for the Protein Data Bank. The study was done by AutoDock 4.2 [46], and the results were analyzed and visualized using DSV [28].

Validation of the docking procedures was performed for each of the six targets. The co-crystallized ligands were re-docked into their corresponding proteins and the binding orientation and interactions were compared with those of the co-crystallized ligands. The results of the validation process are provided in the Supplementary Data (Figures S46–S51).

#### 2.5.1. Docking into the Targets Involved in Inflammation

In addition to their role in inflammation, COX-2 and MAP p38α also play important roles in cell proliferation and apoptosis [2,40]. Several small molecule inhibitors of COXs and MAP p38α exhibited potent antiproliferative activity against several types of solid tumors [2,40]. In the current study, COXs and MAP p38α were identified as potential targets for compound **16g**. Accordingly, a molecular docking study of compound **16g** into the two proteins was performed.

##### Docking into COX-2

**Figure 16:**
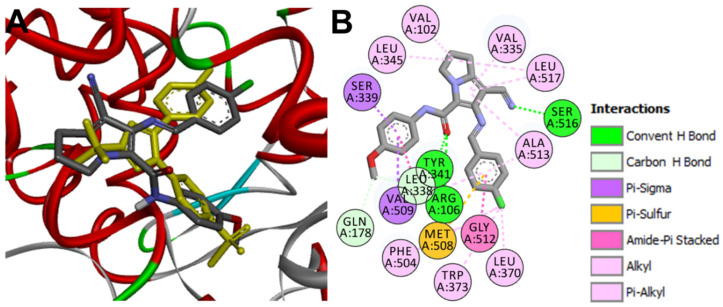
Binding mode of compound **16g** (shown as sticks colored by element) into COX-2 (pdb: 3LN1): (**A**) 3D binding mode of compound **16g** overlaid with celecoxib (yellow sticks); (**B**) 2D binding mode of compound **16g** showing different types of interactions with amino acids in COX-2.

Compound **16g** was docked into the active site of COX-2 (pdb: 3LN1). The results revealed a binding free energy of −10.13 kcal/mol compared to −10.27 kcal/mol for celecoxib. Analysis of the binding mode of compound **16g** revealed superposition of the phenyl rings over the two phenyl rings of celecoxib, Figure 16. The phenyl ring (A) superposed with the sulfonamide-bearing ring in celecoxib, where the methoxy group extended with the sulfonamide group into the side pocket and formed one carbon-hydrogen bond with Gln178. Moreover, the chlorophenyl moiety in compound **16g** superposed partially with the tolyl moiety of celecoxib, where the chloro atom also formed similar hydrophobic interactions with Leu370 and Trp373 like the methyl group in celecoxib, Figure 16.

Investigation of the binding interaction of compound **16g** into COX-2 revealed four conventional hydrogen bonds between the cyano and carbonyl groups with Ser516, Arg106, and Tyr341 amino acids in COX-2, Figure 16. In addition, the methoxy group in compound **16g** formed two carbon–hydrogen bonds with Gln178 and Leu338, similar to the hydrogen bonds formed by the sulfonamido group in celecoxib, Supplementary Data (Figure S46).

##### Docking into MAP p38α

The docking study of compound **16g** into MAP p38α (pdb: 3GCP) [41] was also performed using AutoDock 4.2 [46]. The results showed a higher binding affinity for **16g** (Δ*G_b_* = −10.69 kcal/mol) compared to the co-crystallized ligand, SB2 (Δ*G_b_* = −9.22 kcal/mol).

**Figure 17:**
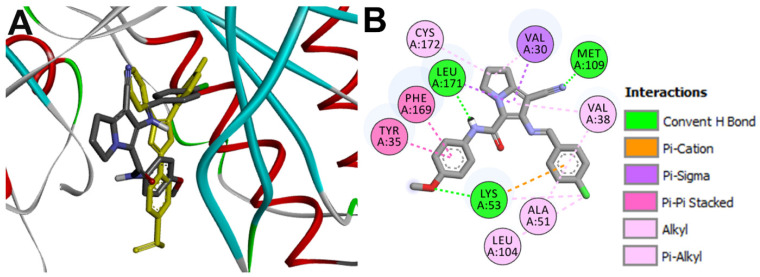
Binding mode of compound **16g** (shown as sticks colored by element) into MAP p38α (pdb: 3GCP): (**A**) 3D binding mode of compound **16g** overlaid with the co-crystallized ligand, SB2 (yellow sticks); (**B**) 2D binding mode of compound **16g** showing different types of interactions with amino acids in p38α.

Analysis of the binding mode revealed partial superposition of the phenyl rings of **16g** over the two phenyl rings in SB2. Moreover, the nitrogen atom of the cyano group in **16g** occupied the same position of the pyridinyl nitrogen of SB2 and also formed one conventional hydrogen bond with Met109, Figure 17.

Investigation of the binding interactions of compound **16g** into MAP p38 showed four conventional hydrogen bonds with the key amino acids Lys53, Met109, and Leu171, Figure 17 Unlike the co-crystallized ligand SB2, no unfavorable interactions or steric clashes were observed between compound **16g** and MAP p38α, Supplementary Data (Figure S47).

#### 2.5.2. Docking Study into Oncogenic Kinases

In addition, a docking study of compound **16g** was performed on the four oncogenic kinases (EGFR, CDK2, BRAF, and VEGFR1) identified in the target prediction test, Table 4. The results were also compared with those of the co-crystallized ligands of the four kinases.

##### Docking into EGFR

**Figure 18:**
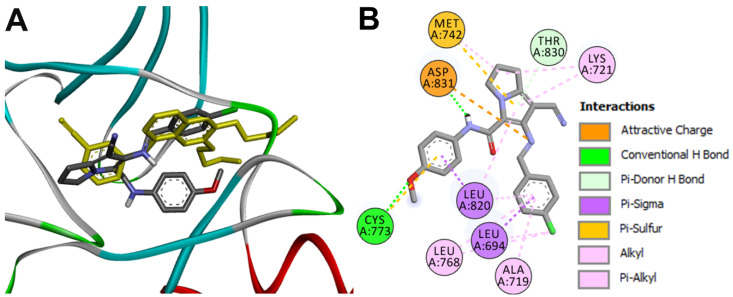
Binding mode of compound **16g** (shown as sticks colored by element) into EGFR (pdb: 1M17): (**A**) 3D binding mode of compound **16g** overlaid with the co-crystallized ligand, erlotinib (yellow sticks); (**B**) 2D binding mode of compound **16g** showing different types of interactions with amino acids in EGFR.

Compound **16g** was docked into EGFR (pdb: 1M17) [42], and the results revealed a significantly higher binding affinity (Δ*G_b_* = −9.52 kcal/mol) compared to the co-crystallized ligand, erlotinib (Δ*G_b_* = −7.39 kcal/mol). The higher affinity of compound **16g** could be attributed to the higher number of hydrogen bonds and to the electrostatic interaction with Asp831. Analysis of the binding interactions of **16g** into EGFR revealed two conventional hydrogen bonds with the key amino acids Cys773 and Asp831 (Figure 18) compared to one conventional hydrogen bond for erlotinib, Supplementary Data (Figure S48).

Investigation of the binding mode of compound **16g** revealed partial superposition of the pyrrole ring over the ethynylphenyl moiety of erlotinib, where the two moieties formed similar binding interactions with Thr830 (carbon-hydrogen bond) and Lys721 (pi-alkyl interaction), Figure 18. The chlorophenyl moiety in **16g** also superposed with the phenyl ring of the quinazoline nucleus in erlotinib and extended into the front pockets, forming similar hydrophobic interactions with Leu694, Ala719, and Leu820 like the 7-methoxyethoxy moieties of erlotinib.

##### Docking into CDK2

**Figure 19:**
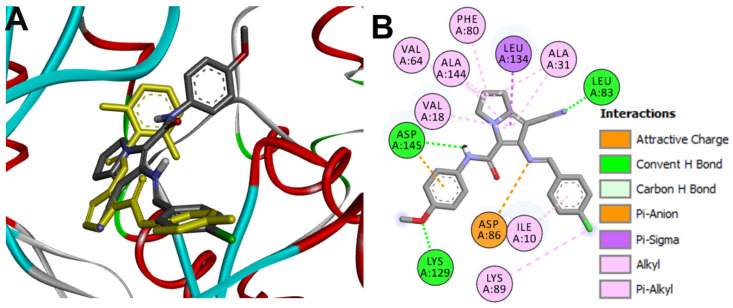
Binding mode of compound **16g** (shown as sticks colored by element) into CDK2 (pdb: 2VTP): (**A**) 3D binding mode of the top-ranked pose of compound **16g** overlaid with the co-crystallized ligand, LZ9 (yellow sticks); (**B**) 2D binding mode of compound **16g** showing different types of interactions with amino acids in CDK2.

To perform a docking study of compound **16g** into CDK2, the pdb: 2VTP [43] was used. The results revealed a significantly higher binding affinity for compound **16g** (Δ*G_b_* = −10.0 kcal/mol) compared to the co-crystallized ligand, LZ9 (Δ*G_b_* = −7.57 kcal/mol). An overlay view of the best ranked pose of compound **16g** into CDK2 revealed partial superposition of the pyrrolizine nucleus over the pyrazole ring in LZ9, where the nitrogen of the cyano group occupied the same position of N^10^ of the pyrazole ring. This nitrogen atom also formed one conventional hydrogen bond with Leu83 like LZ9, Figure 19.

The higher affinity of compound **16g** compared to the co-crystallized ligand (LZ9) is also attributed to the higher amount of hydrogen, Supplementary Data (Figure S49). Compound **16g** formed three conventional hydrogen bonds with Leu83, Lys129, and Asp145 in CDK2, Figure 19.

##### Docking into BRAF

**Figure 20:**
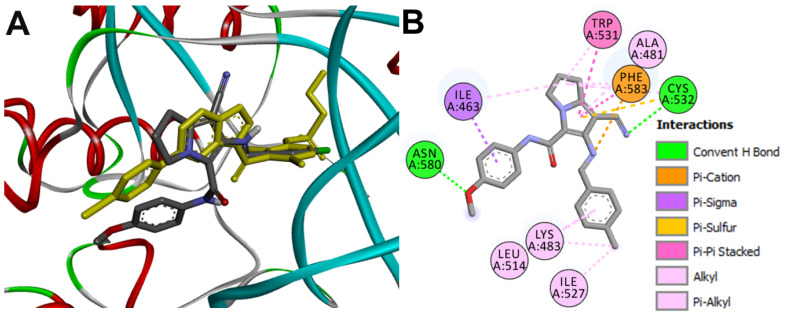
Binding mode of compound **16g** (shown as sticks colored by element) into BRAF (pdb: 4RZV): (**A**) 3D binding mode of best-ranked pose of **16g** overlaid with the co-crystallized ligand, vemurafenib (shown as yellow sticks); (**B**) 2D binding mode of **16g** showing different types of interactions with amino acids BRAF.

Contrary to the docking results for EGFR and CDK2, the results of the docking study of compound **16g** for the wild type BRAF (pdb: 4RZV) [44] revealed a lower binding affinity (Δ*G_b_* = −11.02 kcal/mol) compared to the co-crystallized ligand, vemurafenib (Δ*G_b_* = −12.77 kcal/mol). Investigation of the binding orientation revealed that the pyrrolizine nucleus of compound **16g** partially superposed over the pyrrolo [2,3-*b*]pyridine nucleus of vemurafenib, Figure 20.

In addition, the nitrogen atom of the cyano group in compound **16g** was able to form one conventional hydrogen bond with the key amino acid Cys532 like the pyridine nitrogen in vemurafenib. However, **16g** displayed only two conventional hydrogens with BRAF compared to six hydrogen bonds for vemurafenib, Supplementary Data (Figure S50).

##### Docking into VEGFR1

**Figure 21:**
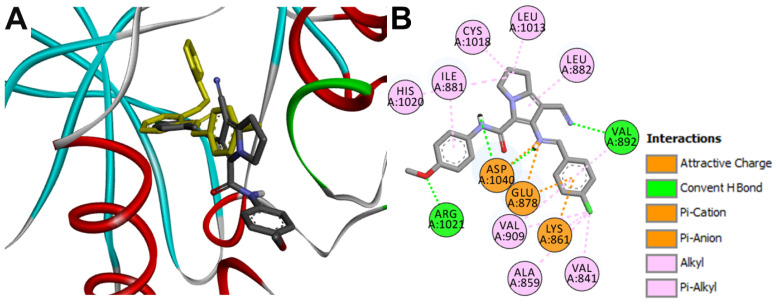
Binding mode of compound **16g** (shown as sticks colored by element) into VEGFR1 (pdb: 3HNG): (**A**) 3D binding mode of the best-ranked pose of compound **16g** overlaid with the co-crystallized ligand, 8ST (yellow sticks); (**B**) 2D binding mode of compound **16g** showing different types of interactions with amino acids in VEGFR1.

The docking study into VEGFR1 (pdb: 3HNG) also revealed a lower binding affinity for compound **16g** (Δ*G_b_* = −10.67 kcal/mol) compared to –12.06 kcal/mol for the co-crystallized ligand, 8ST. The best fit conformation of compound **16g** superposed only partially with the chlorophenyl-carboxamide moiety of 8ST, Figure 21. In addition, compound **16g** also showed a fewer number of hydrogen bonds with VEGFR1 than 8ST, Supplementary Data (Figure S51). These results were also similar to those obtained from the docking study of compounds **16g** into BRAF.

In conclusion, the results of the docking study revealed higher binding affinities for compound **16g** toward four (COX-2, MAP P38α, EGFR, and CDK2) of the six targets compared to their co-crystallized ligands. However, additional in silico studies are needed to support these results.

### 2.6. Molecular Dynamic Simulation

#### 2.6.1. RMSD Analysis and Hydrogen Bond Interaction Estimation

**Figure 22:**
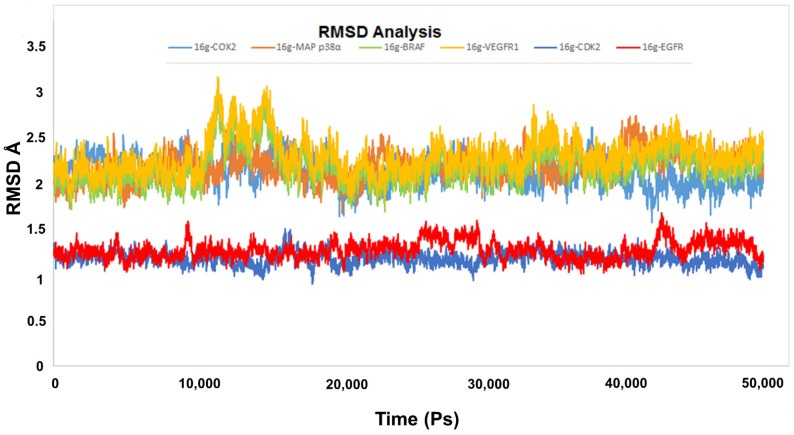
RMSD analysis for **16g** in complex with the six potential targets after 50 ns of MDS.

Molecular dynamic simulations (MDS) have proven to have a high value in many computational studies especially in the accurate determination of the binding affinity and the stability of the ligand-protein complexes. Accordingly, six molecular dynamic experiments were conducted for the synthesized compound **16g** in complex with each of the six potential targets as generated from the docking study. Based on the calculated RMSD values for the enzyme Cα atoms as well as compound **16g** heavy atoms, the two complexes of CDK2 and EGFR bound to **16g** were predicted to be the most stable as depicted from Figure 22.

The maximum RMSD values of the two complexes mentioned only reached 1.5 and 1.7 Å for CDK2 and EGFR, respectively. In contrast, the maximum RMSD values of the other four targets in complex with **16g** reached 2.6, 2.7, 3 and 3.2 Å for COX-2, MAP p38α, BRAF and VEGFR1, respectively. Furthermore, the stability of the hydrogen bond interactions between the lead compound **16g**, and the six potential targets were monitored through the entire MDS. Generally, a hydrogen bond is considered stable and valid when the distance between the hydrogen bond donor and acceptor is kept less than 3.5 Å. This criterion was maintained only in all the formed hydrogen bonds of **16g**-CDK2 and **16g**-EGFR complexes. Some of the hydrogen bonds in the other four complexes were significantly unstable Table 5. The MDS results highlighted a high potentiality for compound **16g** to inhibit both CDK2 and EGFR.

#### 2.6.2. MM-PBSA Calculations

The binding free energy that resulted from the binding of **16g** to each of the potential targets were calculated using the MM-PBSA approach. In this approach, the calculations are based on all trajectories extracted from the MDS, in contrast to the docking score that is based on a single conformation. Accordingly, this technique is considered as a more reliable indicator compared to the energy score obtained from the docking studies. As part of these calculations, the free energy of each component (receptor, ligand, and complex) was calculated by summing its free energy of solvation and molecular mechanics’ potential energy in vacuum. The free energy of solvation includes both the polar solvation energy and nonpolar solvation energy (non-electrostatic, calculated by the solvent accessible surface area; SASA model). Finally, the free energy of binding was calculated by subtracting the energy of the receptor and ligand from the energy of the complex. The calculated types of energies and binding free energy values for the six complexes are summarized in Table 6. The results indicated a higher stability for the 16g-CDK2 and 16g-EGFR complexes than the other four complexes as revealed by the high negative binding free energy of the two complexes. The average binding free energy of the 16g-CDK2 and 16g-EGFR complexes were −401 and 387 KJ/mol, respectively, which suggests a strong and stable binding for **16g** with the two targets. On the other hand, **16g** achieved average binding free energy of −360, −354, −337, and −328 KJ/mol with COX-2, p38α, BRAF, and VEGFR-1. We believe that the results from the MD simulations validated our design and supported our hypothesis for CDK2 and EGFR as potential targets for **16g**.

### 2.7. ADME Study

#### 2.7.1. Physicochemical Properties and Drug-Likeness

To calculate physicochemical properties related to drug-likeness, several web-based tools are available for free. Of these tools, SwissADME (http://www.swissadme.ch) [47] was used to calculate physicochemical properties of compounds **10**, and **16a**–**h**, Table 7. Meanwhile, the calculation of molecular volumes and drug-likeness score (DLS) of these compounds was done using Molsoft (http://molsoft.com/mprop).

The molecular weight of all the new compounds **16a**–**h** was in the range of 386.42–427.50 daltons, which fall within the limits of Lipinski’s rule. They also showed comparable or slightly higher molecular volumes compared to compound **10**. In addition, the number of the hydrogen bond acceptors (H_A_)/donors (**H_D_**) were also within the limits of Lipinski’s rule. Accordingly, no violations from Lipinski’s rule was observed for any of the new compounds **16a–h**.

All of the new compounds exhibited similar bioavailability scores with 81.60–84.79% oral absorption compared to compound **10** (84.79%). The new compounds also exhibited drug-likeness score in the range of 0.25–0.95 compared to 0.80 for compound **10**. Among the new compounds, compound **16g** exhibited the highest DLS.

#### 2.7.2. Metabolic Study

**Figure 23:**
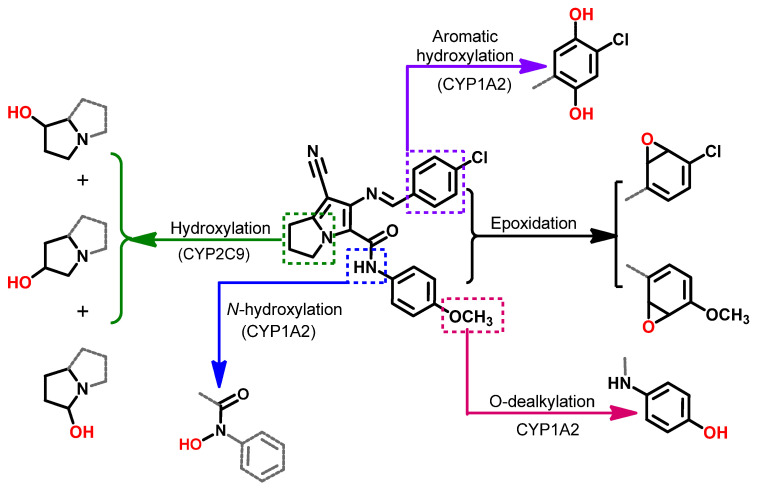
Expected phase I metabolic pathways and metabolites of compound **16g** in humans.

In the current study, Biotransformer (http://biotransformer.ca) [48] was used to predict the metabolic pathways and the expected metabolites of compound **16g**. The test compound was submitted to the server. Thereafter, phase I metabolic transformation was selected. The output results in the form of metabolic pathways were obtained describing the expected metabolites and the transforming phase I enzymes which could perform this action in humans. The results were collected in one figure, Figure 23.

The results of the metabolic study of compounds **16g** revealed five potential metabolic pathways and eight expected metabolites. The metabolic pathways included epoxidation of the aromatic rings, aromatic hydroxylation, *O*-demethylation of the methoxy group, *N*-hydroxylation of the carboxamide nitrogen, and oxidation of one of the secondary carbons of the pyrrolidine ring, Figure 23.

The prediction of phase II metabolites of compound **16g** did not show any results. This was due to the absence of the polar functional groups (OH and COOH), which can undergo conjugation metabolism. However, most of the expected phase I metabolites of compound **16g** (Figure 23) have alcoholic/phenolic OH groups that can undergo glucuronidation of sulfate conjugation.

## 3. Materials and Methods

### 3.1. Pharmacophore Search

The pharmacophore search was done using Pharmit [24]. The compound library was initially uploaded to Pharmit as a compressed file in “sdf.gz.” format. The study was started by selecting the crystal structure of COX-2 (pdb: 1CX2) with the co-crystallized ligand (SC-558). The displayed pharmacophore features were edited to include the pharmacophore features in Figure 4. The settings including hits reduction and screening were set to the default values. The search MolPort was used to select the uploaded compound library. The search type was set to pharmacophore search. The results of the pharmacophore search appeared in a tabular form including codes of the hits, RMSD, MW, and number of RBs.

### 3.2. Chemistry

Chemical reagents and solvents were purchased from Sigma Aldrich (Darmstadt, Germany). Melting points (uncorrected) of compounds **16a**–**h** were determined by an IA 9100MK-Digital melting point apparatus (Cole-Parmer, Vernon Hills, IL, USA). Absorption bands in the infrared (IR) spectra were recorded on a RUKER TENSOR 37 FTIR spectrophotometer. Molecular ions and mass spectra (MS) of compounds **16a**–**h** were analyzed using the Shimadzu Qp-2010 Plus mass spectrometer (EI ionization mode). The elemental analyses (C, H, and N) of compounds **16a**–**h** were measured in a Microanalytical Center, Cairo University. ^1^H-NMR, ^13^C-NMR, and DEPT C^135^ spectra were recorded using BRUKER AVANCE III at 500 MHz, 125 and 125 MHz, respectively.

Preparation of the starting compounds **12**, **14a,b**, and **15a**,**b** was achieved following the previous reports [32,33,34].

Copies of the spectra data including IR, mass, ^1^H-NMR, ^13^C-NMR, and DEPT C^135^ spectra of compounds **16a**–**h** are provided in Supplementary Data (Figures S3–S45).

The readers are advised to consider that the two phenyl rings are assigned as phenyl ring A and B (Figure 9).

#### 3.2.1. General Procedure (A) for Preparation of Compounds (**16a–h**)

The new compounds were prepared from compound **15a**,**b** according to the reported procedures [20]. A mixture of the pyrrolizine **15a**,**b** (3.4 mmol), the appropriate aldehyde (4.4 mmol), and glacial acetic acid (0.5 mL) in absolute ethanol (30 mL) was refluxed for 4–6 h. The reaction mixture was then concentrated and set aside to cool. The solid product obtained was recrystallized from acetone-chloroform.

##### 7-Cyano-6-((4-(dimethylamino)benzylidene)amino)-N-(4-methoxyphenyl)-2,3-dihydro-1H-pyrrolizine-5-carboxamide (**16a**)

Compound **15a** (1 g, 3.4 mmol) was refluxed with 4-(dimethylamino)benzaldehyde (0.65 g, 4.4 mmol) according to the general procedure A to afford compound **16a** as a yellow solid product, m.p. 226-8 °C, yield 58%. IRʋ_max_/cm^−1^ 3218 (NH), 3076, 3002, (aromatic C-H), 2958, 2904, 2830 (aliphatic C-H), 2209 (CN), 1656 (C=O), 1583, 1539, 1509 (C=C, C=N). ^1^H-NMR (CDCl_3_-500 MHz, *δ* ppm): *δ* 2.55 (m, 2H, CH_2_-2), 3.04 (t, 2H, *J* = 6.8 Hz, CH_2_-1), 3.13 (s, 6H, N(CH_3_)_2_), 3.83 (s, 3H, OCH_3_), 4.52 (t, 2H, *J* = 7.4 Hz, CH_2_-3), 6.79 (d, 2H, *J* = 7.6 Hz, phenyl B CH-3+CH-5), 6.92 (d, 2H, *J* = 7.2 Hz, phenyl A CH-3+CH-5), 7.62 (d, 2H, *J* = 7.4 Hz, phenyl B CH-2+CH-6), 7.82 (d, 2H, *J* = 7.7 Hz, phenyl A CH-2+CH-6), 8.98 (s, 1H, (N=CH), 10.82 (s, 1H, CONH). ^13^C-NMR (CDCl_3_-125 MHz, *δ* ppm): *δ* 24.59 (CH_2_-2), 25.43 (CH_2_-2), 40.27 (N(CH_3_)_2_), 49.98 (CH_2_-3), 55.53 (OCH_3_), 111.85 (phenyl B CH-3 + CH-5), 114.22 (phenyl A CH-3 + CH-5), 116.71 (C-7), 116.76 (CN), 121.25 (phenyl A CH-2 + CH-6), 122.06 (phenyl B CH-2 + CH-6), 123.15 (C-6), 130.90 (phenyl A C-1), 131.87 (phenyl B C-1), 147.61 (C-5), 153.17 (C-7a), 155.94 (phenyl B C-4), 158.75 (phenyl A C-4), 159.59 (N=CH), 163.43 (CO). DEPT C^135^ (CDCl_3_-125 MHz, *δ* ppm): *δ* 24.59 (CH_2_-2), 25.43 (CH_2_-2), 40.28 (N(CH_3_)_2_), 49.98 (CH_2_-3), 55.54 (OCH_3_), 111.86 (phenyl B CH-3 + CH-5), 114.22 (phenyl A CH-3 + CH-5), 121.25 (phenyl A CH-2 + CH-6), 122.06 (phenyl B CH-2 + CH-6), 159.59 (N=CH). MS (EI): m/z (%) 427 (M^+^, 28), 411 ([M-16]^+^, 43), 402 (33), 312 ([M-15]^+^, 52), 287 (88), 261 (81), 256 (85), 227 (38), 218 (42), 191 (67), 147 (33), 72 (57), 52 (100). Anal. Calcd. for C_25_H_25_N_5_O_2_ (427.50): C, 70.24; H, 5.89; N, 16.38. Found: C, 69.85; H, 5.62; N, 16.84.

##### 7-Cyano-6-((4-(dimethylamino)benzylidene)amino)-N-(4-fluorophenyl)-2,3-dihydro-1H-pyrrolizine-5-carboxamide (**16b**)

Compound **15b** (0.96 g, 3.4 mmol) was refluxed with 4-(dimethylamino)benzaldehyde (0.65 g, 4.4 mmol) according to the general procedure A to afford compound **16b** as a yellow solid product, m.p. 244-6 °C, yield 54%. IRʋ_max_/cm^−1^ 3236 (NH), 3061 (aromatic C-H), 2997, 2907, 2822 (aliphatic C-H), 2213 (CN), 1660 (C=O), 1611, 1580, 1529 (C=C, C=N). ^1^H-NMR (CDCl_3_-500 MHz, *δ* ppm): *δ* 2.57 (m, 2H, CH_2_-2), 3.05 (t, 2H, *J* = 6.5 Hz, CH_2_-1), 3.17 (s, 6H, N(CH_3_)_2_), 4.49 (t, 2H, *J* = 6.4 Hz, CH_2_-3), 6.86 (d, 2H, *J* = 7.4 Hz, phenyl B CH-3+CH-5), 7.05 (d, 2H, *J* = 8.3 Hz, phenyl A CH-3 + CH-5), 7.72 (broad s, 2H, phenyl B CH-2 + CH-6), 7.95 (broad s, 2H, phenyl A CH-2 + CH-6), 8.77 (s, H, N=CH), 11.00 (s, H, CONH). ^13^C-NMR (CDCl_3_-125 MHz, *δ* ppm): *δ* 24.72 (CH_2_-2), 25.48 (CH_2_-1), 40.67 (N(CH_3_)_2_), 49.98 (CH_2_-3), 111.19 (phenyl B CH-3 + CH5), 112.56 (phenyl B CH-2 + CH-6), 115.57 (d, *J* = 22.3 Hz, phenyl A CH-3 + CH-5), 115.92 (C-7), 116.54 (CN), 117.21 (C-6), 121.58 (d, *J* = 7.8 Hz, phenyl A CH-2 + CH6), 130.46 (phenyl B C-1), 132.34 (C-5), 134.59 (d, *J* = 2.8 Hz, phenyl A C-1), 147.62 (C-7a), 152.24 (phenyl B C-4), 158.43 (N=CH), 159.11 (d, *J* = 242.7 Hz, phenyl A C-4), 160.48 (CO). DEPT C^135^ (CDCl_3_-125 MHz, *δ* ppm): *δ* 24.72 (CH_2_-2), 25.48 (CH_2_-1), 40.67 (N(CH_3_)_2_), 49.98 (CH_2_-3), 111.19 (phenyl B CH-3 + CH5), 112.61 (phenyl B CH-2 + CH6), 115.57 (d, *J* = 22.3 Hz, phenyl A CH-3 + CH5), 121.58 (d, *J* = 7.8 Hz, phenyl A CH-2 + CH6), 158.43 (N=CH). MS (EI): m/z (%) 415 (M^+^, 17), 412 ([M-3]^+^, 48), 395 (37), 384 (100), 346 (34), 301 (48), 295 (86), 253 (49), 223 (37), 199 (49), 173 (62), 144 (68), 105 (79). Anal. Calcd. for C_24_H_22_FN_5_O (415.46): C, 69.38; H, 5.34; N, 16.86. Found: C, 68.91; H, 5.77; N, 17.14.

##### 7-Cyano-N-(4-methoxyphenyl)-6-((4-methylbenzylidene)amino)-2,3-dihydro-1H-pyrrolizine-5-carboxamide (**16c**)

Compound **15a** (1 g, 3.4 mmol) was refluxed with 4-methylbenzaldehyde (0.53 g, 4.4 mmol) according to the general procedure A to afford compound **16c** as a yellow solid product, m.p. 231-3°C, yield 61%. IRʋ_max_/cm^−1^ 3230 (NH), 3061, 3000 (aromatic C-H), 2948, 2830 (aliphatic C-H), 2209 (CN), 1663 (C=O), 1600, 1549, 1509 (C=C, C=N). ^1^H-NMR (CDCl_3_-500 MHz, *δ* ppm): *δ* 2.47 (s, 3H, CH_3_), 2.54 (m, 2H, CH_2_-2), 3.01 (t, 2H, *J* = 7.1 Hz, CH_2_-1), 3.83 (s, 3H, OCH_3_), 4.51 (t, 2H, *J* = 7.3 Hz, CH_2_-3), 6.91 (d, 2H, *J* = 7.8 Hz, phenyl A CH-3′+CH-5′), 7.34 (d, 2H, *J* = 7.1 Hz, phenyl B CH-3″+CH-5″), 7.58 (d, 2H, *J* = 7.8 Hz, phenyl A CH-2′+CH-6′), 7.80 (d, 2H, *J* = 7.1 Hz, phenyl B CH-2″+CH-6″), 9.11 (s, 1H, N=CH), 10.60 (s, 1H, CONH). ^13^C-NMR (CDCl_3_-125 MHz, *δ* ppm): *δ* 21.78 (CH_3_), 24.50 (CH_2_-2), 25.41 (CH_2_-1), 50.08 (CH_2_-3), 55.53 (OCH_3_), 114.25 (phenyl A CH-3 + CH-5), 116.40 (C-7), 117.75 (CN), 121.08 (phenyl A CH-2 + CH-6), 128.75 (phenyl B CH-2 + CH-6), 129.73 (C-6), 129.90 (phenyl B CH-3 + CH-5), 131.61 (phenyl A C-1), 132.79 (phenyl B C-1), 139.10 (C-5), 143.24 (C-7a), 148.04 (phenyl B C-4), 156.07 (phenyl A C-4), 158.32 (CO), 159.49 (N=CH). DEPT C^135^ (CDCl_3_-125 MHz, *δ* ppm): *δ* 21.78 (CH_3_), 24.50 (CH_2_-2), 25.41 (CH_2_-1), 50.08 (CH_2_-3), 55.53 (OCH_3_), 114.25 (phenyl A CH-3 + CH-5), 121.08 (phenyl A CH-2 + CH-6), 128.75 (phenyl B CH-2 + CH-6), 129.90 (phenyl B CH-3 + CH-5), 159.49 (N=CH). MS (EI): m/z (%) 399 ([M+1]^+^, 13), 393 ([M-5]^+^, 17), 382 ([M-16]^+^, 14), 377 (56), 364 (31), 336 (37), 318 (89), 287 (100), 275 (34), 240 (13), 212 (26), 198 (31), 171 (23), 154 (22), 92 (24). Anal. Calcd. for C_24_H_22_N_4_O_2_ (398.46): C, 72.34; H, 5.57; N, 14.06. Found: C, 72.81; H, 5.69; N, 13.84.

##### 7-Cyano-N-(4-fluorophenyl)-6-((4-methylbenzylidene)amino)-2,3-dihydro-1H-pyrrolizine-5-carboxamide (**16d**)

Compound **15b** (0.96 g, 3.4 mmol) was refluxed with 4-methylbenzaldehyde (0.53 g, 4.4 mmol) according to the general procedure A to afford compound **16d** as a yellow solid product, m.p. 241-3 °C, yield 59%. IRʋ_max_/cm^−1^ 3235 (NH), 3066 (aromatic C-H), 2955, 2924, 2858 (aliphatic C-H), 2214 (CN), 1664 (C=O), 1601 1551, 1506 (C=C, C=N), 1208 (C-F). ^1^H-NMR (CDCl_3_-500 MHz, *δ* ppm): *δ* 2.47 (s, 3H, CH_3_), 2.56 (m, 2H, CH_2_-2), 3.02 (t, 2H, *J* = 6.2 Hz, CH_2_-1), 4.51 (t, 2H, *J* = 6.9 Hz, CH_2_-3), 7.05 (d, 2H, *J* = 8.1 Hz, phenyl A CH-3′+CH-5′), 7.34 (d, 2H, *J* = 6.9 Hz, phenyl B CH-3″+CH-5″), 7.62 (d, 2H, *J* = 7.8 Hz, phenyl A CH-2′+CH-6′), 7.80 (d, 2H, *J* = 6.6 Hz, phenyl B CH-2″+CH-6″), 9.11 (s, 1H, N=CH), 10.71 (s, 1H, CONH). ^13^C-NMR (CDCl_3_-125 MHz, *δ* ppm): *δ* 21.79 (CH_3_), 24.53 (CH_2_-2), 25.40 (CH_2_-1), 50.11 (CH_2_-3), 115.71 (d, *J* = 22.4 Hz, phenyl A CH-3 + CH-5), 116.25 (C-7), 117.46 (CN), 121.09 (d, *J* = 7.9 Hz, phenyl A CH-2 + CH-6), 128.75 (phenyl B CH-2+ CH-6), 129.73 (C-6), 129.95 (phenyl B CH-3 + CH-5), 132.69 (phenyl B C-1), 134.46 (d, *J* = 2.8 Hz, phenyl A C-1), 139.37 (C-5), 143.45 (C-7a), 148.23 (phenyl B C-4), 158.10, 158.46 (CO), 159.07 (d, *J* = 243.0 Hz, phenyl A C-4), 159.77 (N=CH). DEPT C^135^ (CDCl_3_-125 MHz, *δ* ppm): *δ* 21.79 (CH_3_), 24.53 (CH_2_-2), 25.40 (CH_2_-1), 50.11 (CH_2_-3), 115.71 (d, *J* = 22.4 Hz, phenyl A CH-3 + CH-5), 121.09 (d, *J* = 7.9 Hz, phenyl A CH-2 + CH-6), 128.76 (phenyl B CH-2+ CH-6), 129.95 (phenyl B CH-3 + CH-5), 159.77 (N=CH). MS (EI): m/z (%) 386 (M^+^, 51), 383 ([M-3]^+^, 2), 370 ([M-16]^+^, 73), 356 ([M-30]^+^, 38), 341 (65), 326 (22), 308 (33), 285 (29), 247 (26), 216 (17), 193 (100), 165 (61), 133 (44), 110 (58). Anal. Calcd. for C_23_H_19_FN_4_O (386.42): C, 71.49; H, 4.96; N, 14.50. Found: C, 71.62; H, 4.69; N, 14.43.

##### 7-Cyano-6-((4-fluorobenzylidene)amino)-N-(4-methoxyphenyl)-2,3-dihydro-1H-pyrrolizine-5-carboxamide (**16e**)

Compound **15a** (1 g, 3.4 mmol) was refluxed with 4-flourobenzaldehyde (0.54 g, 4.4 mmol) according to the general procedure A to afford compound **16e** as a yellow solid product, m.p. 239-41 °C, yield 67%. IRʋ_max_/cm^−1^ 3233 (NH), 3070, 3007 (aromatic C-H), 2955, 2890 (aliphatic C-H), 2210 (CN), 1662 (C=O), 1600, 1584, 1509 (C=C, C=N), 1229 (C-F). ^1^H-NMR (CDCl_3_-500 MHz, *δ* ppm): *δ* 2.57 (m, 2H, CH_2_-2), 3.05 (t, 2H, *J* = 7.1 Hz, CH_2_-1), 3.84 (s, 3H, OCH_3_), 4.54 (t, 2H, *J* = 7.1 Hz, CH_2_-3), 6.93 (d, 2H, *J* = 8.9 Hz, phenyl A CH-3 + CH-5), 7.23 (d, 2H, *J* = 8.3 Hz, phenyl B CH-3 + CH-5), 7.56 (d, 2H, *J* = 8.8 Hz, phenyl A CH-2 + CH-6), 7.93 (d, 2H, *J* = 7.0 Hz, phenyl B CH-2+CH-6), 9.14 (s, 1H, N=CH), 10.46 (s, 1H, CONH). ^13^C-NMR (CDCl_3_-125 MHz, *δ* ppm): *δ* 24.54 (CH_2_-2), 25.45 (CH_2_-1), 50.15 (CH_2_-3), 55.54 (OCH_3_), 114.33 (phenyl A CH-3 + CH-5), 114.80 (C-7), 116.50 (d, *J* 22.2 Hz, phenyl B CH-3 + CH-5), 117.97 (CN), 121.19 (phenyl A CH-2 + CH-6), 129.59 (C-6), 130.73 (d, *J* = 8.9 Hz, phenyl B CH-2 + CH-6), 131.39 (phenyl C-1), 131.80 (d, *J* = 3.0 Hz, phenyl B C-1), 132.23 (C-5), 138.80 (C-7a), 148.10 (phenyl A C-4), 156.22 (CO), 158.16 (N=CH), 165.27 (d, *J* = 254.5 Hz, phenyl B C-4). DEPT C^135^ (CDCl_3_-125 MHz, *δ* ppm): *δ* 24.54 (CH_2_-2), 25.45 (CH_2_-1), 50.15 (CH_2_-3), 55.54 (OCH_3_), 114.34 (phenyl A CH-3 + CH-5), 116.51 (d, *J* = 22.2 Hz, phenyl B CH-3 + CH-5), 121.19 (phenyl A CH-2 + CH-6), 130.73 (d, *J* = 8.9 Hz, phenyl B CH-2 + CH-6), 158.16 (N=CH). MS (EI): m/z (%) 403 ([M+1]^+^, 52), 402 (M^+^, 28), 401 ([M-1]^+^, 12), 386 ([M-16]^+^, 100), 375 ([M-28]^+^, 19), 356 (68), 331 (23), 315 (64), 252 (58), 226 (17), 178 (23), 165 (23), 137 (49), 99 (24). Anal. Calcd. for C_23_H_19_FN_4_O_2_ (402.42): C, 68.65; H, 4.76; N, 13.92. Found: C, 68.31; H, 4.32; N, 13.84.

##### 7-Cyano-6-((4-fluorobenzylidene)amino)-N-(4-fluorophenyl)-2,3-dihydro-1H-pyrrolizine-5-carboxamide (**16f**)

Compound **15b** (0.96 g, 3.4 mmol) was refluxed with 4-flourobenzaldehyde (0.54 g, 4.4 mmol) according to the general procedure A to afford compound **16f** as a yellow solid product, m.p. 253-5°C, yield 66%. IRʋ_max_/cm^−1^ 3222 (NH), 3072, 3003 (aromatic C-H), 2966, 2905 (aliphatic C-H), 2212 (CN), 1664 (C=O), 1600, 1585, 1553, 1507 (C=C, C=N), 1230 (C-F). ^1^H-NMR (CDCl_3_-500 MHz, *δ* ppm): *δ* 2.59 (m, 2H, CH_2_-2), 3.08 (t, 2H, *J* = 6.8 Hz, CH_2_-1), 4.55 (t, 2H, *J* = 7.1 Hz, CH_2_-3), 7.07 (d, 2H, *J* = 7.9 Hz, phenyl A CH-3 + CH-5), 7.25 (d, 2H, *J* = 7.9 Hz, phenyl B CH-3 + CH-5), 7.61 (d, 2H, *J* = 8.4 Hz, phenyl A CH-2 + CH-6), 7.95 (d, 2H, *J* = 8.5 Hz, phenyl B CH-2 + CH-6), 9.16 (s, 1H, N=CH), 10.57 (s, 1H, CONH). ^13^C-NMR (CDCl_3_-125 MHz, *δ* ppm): *δ* 24.60 (CH_2_-2), 25.46 (CH_2_-3), 50.18 (CH_2_-3), 115.82 (d, *J* = 22.4 Hz, phenyl B CH-3 + CH-5), 116.15 (C-7), 116.31 (CN), 116.60 (d, *J* = 22.2 Hz, phenyl A CH-3 + CH-5), 117.69 (C-6), 121.27 (d, *J* = 7.6 Hz, phenyl A CH-2 + CH-6), 130.50 (d, *J* = 8.4 Hz, F-Ph (B) C-1), 130.83 (d, *J* = 8.3 Hz, phenyl B CH-2+CH-3), 131.65 (C-5), 132.27 (d, *J* = 9.5 Hz), 134.23 (C-7a), 158.39 (CO), 158.63 (N=CH), 159.21 (d, *J* = 243.6 Hz, F-Ph (A) C-4), 163.42 (d, *J* = 240.0 Hz, F-Ph (B) C-4). DEPT C^135^ (CDCl_3_-125 MHz, *δ* (ppm): *δ* 24.60 (CH_2_-2), 25.46 (CH_2_-3), 50.18 (CH_2_-3), 115.82 (d, *J* = 22.4 Hz, phenyl B CH-3 + CH-5), 116.60 (d, *J* = 22.2 Hz, phenyl A CH-3 + CH-5), 121.27 (d, *J* = 7.6 Hz, phenyl A CH-2 + CH-6), 130.83 (d, *J* = 8.3 Hz, phenyl B CH-2 + CH-3), 158.63 (N=CH). MS (EI): m/z (%) 391 ([M+1]^+^, 27), 388 ([M-2]^+^, 12), 352 (12), 341 (100), 328 (15), 300 (34), 287 (24), 257 (35), 208 (26), 192 (32), 164 (48), 149 (38), 110 (20), 83 (27). Anal. Calcd. for C_22_H_16_F2N_4_O (390.39): C, 67.69; H, 4.13; N, 14.35. Found: C, 68.16; H, 3.67; N, 14.76.

##### 6-((4-Chlorobenzylidene)amino)-7-cyano-N-(4-methoxyphenyl)-2,3-dihydro-1H-pyrrolizine-5-carboxamide (**16g**)

Compound **15a** (1 g, 3.4 mmol) was refluxed with 4-chlorobenzaldehyde (0.62 g, 4.4 mmol) according to the general procedure A to afford compound **16g** as a yellow solid product, m.p. 241-3 °C, yield 72%. IRʋ_max_/cm^−1^ 3281, 3238, 3133 (NH), 3057, 3000 (aromatic C-H), 2970, 2907, 2830 (aliphatic C-H), 2209 (CN), 1663 (C=O), 1592, 1550, 1509 (C=C, C=N), 825 (C-Cl). ^1^H-NMR (CDCl_3_-500 MHz, *δ* ppm): *δ* 2.56 (m, 2H, CH_2_-2), 3.02 (t, 2H, *J* = 7.2 Hz, CH_2_-1), 3.84 (s, 3H, OCH_3_), 4.52 (t, 2H, *J* = 7.1 Hz, CH_2_-3), 6.92 (d, 2H, *J* = 7.2 Hz, phenyl A CH-3 + CH-5), 7.51 (d, 2H, *J* = 8.0 Hz, phenyl A CH-2 + CH-6), 7.55 (d, 2H, *J* = 7.9 Hz, phenyl B CH-3 + CH-5), 7.83 (d, 2H, *J* = 7.8 Hz, phenyl B CH-2 + CH-6), 9.11 (s, 1H, N=CH), 10.42 (s, 1H, CONH). ^13^C-NMR (CDCl_3_-125 MHz, *δ* ppm): *δ* 24.50 (CH_2_-2), 25.41 (CH_2_-1), 50.17 (CH_2_-3), 55.53 (OCH_3_), 114.33 (phenyl A CH-3 + CH-5), 116.30 (C-7), 118.17 (CN), 121.07 (phenyl A CH-2 + CH-6), 129.51 (phenyl B CH-3 + CH-5), 129.69 (phenyl B CH-2 + CH-6), 130.93 (C-6), 131.37 (phenyl A C-1), 133.88 (C-5), 138.47 (phenyl B C-1), 138.48 (C-7a), 148.26 (phenyl B C-4), 156.20 (phenyl A C-4), 157.89 (N=CH), 158.10 (CO). DEPT^135^ (CDCl_3_-125 MHz, *δ* (ppm): *δ* (ppm): 24.49 (CH_2_-2), 25.41 (CH_2_-1), 50.17 (CH_2_-3), 55.53 (OCH_3_), 114.33 (phenyl A CH-3 + CH-5), 121.06 (phenyl A CH-2 + CH-6), 129.51 (phenyl B CH-3 + CH-5), 129.69 (phenyl B CH-2 + CH-6), 157.89 (N=CH). MS (EI): m/z (%) 418 (M^+^, 21), 417 ([M-1]^+^, 20), 413 ([M-5]^+^, 10), 412 ([M-6]^+^, 16), 368 (88), 354 (100), 328 (49), 317 (26), 292 (19), 278 (13), 229 (11), 179 (10), 165 (11), 113 (10), 97 (9). Anal. Calcd. for C_23_H_19_ClN_4_O_2_ (418.88): C, 65.95; H, 4.57; N, 13.38. Found: C, 66.34; H, 4.93; N, 13.24.

##### 6-((4-Chlorobenzylidene)amino)-7-cyano-N-(4-fluorophenyl)-2,3-dihydro-1H-pyrrolizine-5-carboxamide (**16h**)

Compound **15b** (0.96 g, 3.4 mmol) was refluxed with 4-chlorobenzaldehyde (0.62 g, 4.4 mmol) according to the general procedure A to afford compound **16h** as a yellow solid product, m.p. 262-4 °C, yield 63%. IRʋ_max_/cm^−1^ 3274 (NH), 3073, 3005 (aromatic C-H), 2955 (aliphatic C-H), 2209 (CN), 1669 (C=O), 1612, 1554, 1504 (C=C, C=N), 1205 (C-F), 835 (C-Cl). ^1^H-NMR (CDCl_3_-500 MHz, *δ* ppm): *δ* 2.56 (m, 2H, CH_2_-2), 3.06 (t, 2H, *J* = 6.8 Hz, CH_2_-1), 4.54 (t, 2H, *J* = 6.9 Hz, CH_2_-3), 7.09 (d, 2H, *J* = 8.2 Hz, phenyl A CH-3 + CH-5), 7.53 (d, 2H, *J* = 8.0 Hz, phenyl B CH-3 + CH-5), 7.60 (d, 2H, *J* = 7.1 Hz, phenyl A CH-2 + CH-6), 7.85 (d, 2H, *J* = 8.2 Hz, phenyl B CH-2 + CH-6), 9.15 (s, 1H, N=CH), 10.54 (s, 1H, CONH). ^13^C-NMR (CDCl_3_-125 MHz, *δ* ppm): *δ* 24.57 (CH_2_-2), 25.45 (CH_2_-1), 50.20 (CH_2_-3), 115.84 (d, *J* = 22.4 Hz, phenyl A CH-3 + CH-5), 116.15 (C-7), 117.88 (CN), 121.20 (d, *J* = 7.0 Hz, phenyl CH-2 + CH-6), 129.49 (C-6), 129.59 (phenyl B CH-3 + CH-5), 129.72 (phenyl B CH-2 + CH-6), 130.94 (C-5), 133.83 (C-7a), 143.22 (d, *J* = 2.8 Hz, phenyl A C-1), 138.86 (phenyl B C-1), 148.42 (phenyl B C-4), 158.32 (CO), 158.33 (N=CH), 159.20 (d, *J* = 243.2 Hz, phenyl A C-4). DEPT C^135^ (CDCl_3_-125 MHz, *δ* ppm): *δ* 24.57 (CH_2_-2), 25.45 (CH_2_-1), 50.20 (CH_2_-3), 115.84 (d, *J* = 22.4 Hz, phenyl A CH-3 + CH-5), 121.20 (d, *J* = 7.0 Hz, phenyl A CH-2 + CH-6), 129.60 (phenyl B CH-3 + CH-5), 129.72 (phenyl B CH-2 + CH-6), 158.33 (N=CH). MS (EI): m/z (%) 406 (M^+^, 8), 403 ([M-3]^+^, 41), 387 (14), 365 (16), 341 (100), 317 (24), 273 (68), 260 (36), 202 (28), 136 (35), 121 (52), 95 (44). Anal. Calcd. for C_22_H_16_ClFN_4_O (406.84): C, 64.95; H, 3.96; N, 13.77. Found: C, 64.79; H, 4.28; N, 13.92.

### 3.3. Biological Evaluation

#### 3.3.1. Antiproliferative Activity

##### Cell Culture

In the current study, the three cancer cell lines (MCF7, A2780, and HT29) used were obtained from the ATCC. The cell lines were cultured following our previous report [20]. On the other hand, the normal MRC5 cells used in the current study were maintained in Eagle’s minimum essential medium following the previous report [33].

##### Antiproliferative Activity Assay

The antiproliferative activity was measured using the MTT assay according to the previous report [35]. Briefly, the cancer/normal cell lines were cultured in 96-well (3 × 10^3^/well) separately. The cells were treated by tested compounds **16a**–**h** in final concentrations of 0, 0.1, 1, 10, 25, and 50 μM and incubated at 37 °C for 72 h. The MTT was added and the antiproliferative activities (IC_50_ values) of the new compounds were calculated.

#### 3.3.2. Cell Cycle Analysis

The effect of compound **16g** on the cell cycle distribution of MCF7 was performed following the previous report [36]. Briefly, the cancer cells were cultured for 72 h with 0.00, 0.01, 0.05, and 0.10 μM final concentration of compound **16g**. Next, the cells were washed with PBS x1 and trypsinized. The collected cells were spinned and fixed with 70% ethanol. Ribonuclease A was added (15 min) after suspending the cells in cold PBS x1. Following the addition of propidium iodide, analysis of the ice-cold cells was done by flow cytometry (BC, FC500, Brea, CA, USA).

#### 3.3.3. Annexin V FITC/PI Assay

MCF7 cells were treated by compound **16g** at 0.00, 0.01, 0.05 and 0.10 μM final concentrations for 72 h. Briefly, the superanent of the cells was collected in ice-cold tubes, trypsinized and incubated (37 °C). The procedures were completed following the previous report [37]. The samples were analyzed using flow cytometry (BC, FC500, Brea, CA, USA).

### 3.4. Target Prediction

The SwissTargetPrediction (http://www.swisstargetprediction.ch) [39] was used to predict the molecular targets of the new compounds **16a**–**h**. The test compounds were submitted one by one to the server. After running the prediction process, the results were obtained in the form of a pie chart for each compound including the major classes of the potential targets ranging with their percent. In addition, a detailed report for the first 100 entry of these targets was also obtained.

### 3.5. Molecular Docking

The molecular docking study of compound **16g** into COX2 (pdb: 3LN1) [26], MAP p38α (pdb: 3GCP) [41], EGFR (pdb: 1M17) [42], CDK2 (pdb: 2VTP) [43], BRAF (pdb: 4RZV) [44], and VEGFR1 (pdb: 3HNG) was performed using AutoDock 4.2. [46]. Preparation of ligands/protein files [49], grid, and docking parameters files [49,50] was done following the previous reports. The crystal structures of the six proteins were obtained from the protein data bank. A 3D grid box of 60 × 60 × 60 Å size (x, y, z) with the spacing of 0.375 Å centered at 30.9, −22.3, and −16.5 Å for docking into COX-2, at 22.5, 0.3, and −19.2 Å for docking into MAP p38α, at 22.0, 0.25, and 52.8 Å for docking into EGFR, at 27.7, 6.8, and 63.4 Å for docking into CDK2, at 77.9, 11.5, and 12.0 Å for docking into BRAF and centered at 4.7, 17.8, and 33.4 Å for docking into VEGFR1. DSV [28] was used in the analysis and visualization of the docking results.

### 3.6. Molecular Dynamic Simulation

#### 3.6.1. RMSD Analysis and Hydrogen Bond Interaction Estimation

In this section, GROMACS 5.1 software was used to conduct all the molecular dynamics simulations [51]. Six MDS experiments were conducted on the six complexes retrieved from the docking step; each complex contains **16g** bound to a different target (CDK2, COX2, EGFR, VEGFR1, BRAF, or MAP p38α). The Automated Topology Builder (ATB) and Repository version 3 [52] were implemented to generate a topology file for 16g under the GROMOS96 force field. The generated ligand topology was joined with each of the six enzymes’ topology using the standard published protocol [53]. Solvation of the six complexes was done using single point charge (SPC) water model. A proper number of ions was added to the processed systems using the gmx genion script. The neutralized solvated systems were energy minimized using the steepest descent minimization algorithm with a maximum of 50,000 steps and <10.0 kJ/mol under GROMOS96 43a1 force field [54]. After that, two equilibration ensembles were conducted to ensure proper equilibrations for all the processed systems. At the beginning, an NVT ensemble with a constant number of particles, volume, and temperature (310 K) was done for 1 ns then followed by an NPT ensemble with a constant number of particles, pressure, and temperature for 4 ns. The Particle Mesh Ewald (PME) method with a 12 Å cut-off and 12 Å Fourier spacing were used to get the long range electrostatic [53]. The six equilibrated systems entered the production stage without any restraints for 50 ns with a time step of 2 fs, and the structural coordinates were saved every 5 ps. Both the temperature (310k) and the pressure (1atm) were regulated throughout the simulation V-rescale weak coupling method (modified Berendsen thermostat) and the Parrinello–Rahman method [55,56]. The RMSD of the whole system was calculated from the generated trajectories from the production step as well as the distances of the formed HBs.

#### 3.6.2. MM-PBSA Calculation

Binding free energy calculations were performed using the *MM-PBSA* which applies the following equation:

ΔG_(Binding)_ = G_(Complex)_ − G_(Receptor)_ − G_(Ligand)_

G_(Complex)_ is the total free energy of the ligand–protein complex. G_(Receptor)_ and G_(Ligand)_ are total free energies of the isolated protein and ligand in solvent, respectively. The total free energy was calculated for all MD trajectories from its molecular mechanics potential energy plus the energy of solvation using the g_mmpbsa package implemented in GROMACS software [57]. Individual energies and the corresponding SD were calculated and then summed together to yield the average total free energy of each.

### 3.7. ADME Study

#### 3.7.1. Physicochemical Properties and Drug-Likeness

The physicochemical properties of compounds **16a**–**h** were calculated by the SwissADME webserver (http://www.swissadme.ch/) [47]. Each compound was submitted to the server followed by running the calculations. On the other hand, the Molsoft webserver (http://molsoft.com/mprop/) was used in the calculation of drug-likeness scores (DLSs) of the final compounds.

#### 3.7.2. Metabolic Study

Biotransformer (http://biotransformer.ca) [48] was used to predict the metabolic pathways and the metabolites of compound **16g**. The test compound was submitted in the server. Thereafter, the task of metabolic transformation was selected (phase I/II), and the number of reaction steps was set to one. The output results in the form of metabolic pathways/expected metabolites were obtained.

## 4. Conclusions

In the current study, an in silico approach based on free software was used to optimize the antiproliferative activity and investigate the potential mechanism of action of a series of pyrrolizine-based Schiff bases. A compound library of 288 Schiff base derivatives was designed based on compound **10**. A pharmacophore of the compound library search was performed. Structural analysis of the top-scoring hits was conducted, and a preliminary docking study into COX-2 was performed to select the promising hits for the synthesis. The chemical synthesis and structural elucidation of the new compounds **16a**–**h** were discussed. The MTT assay was used to evaluate the antiproliferative activity of compounds **16a**–**h** against MCF-7, A2780, and HT29 cancer lines (IC_50_ = 0.01–40.50 μM). Amongst the new compounds, compound **16g** exhibited the highest antiproliferative activity against MCF7 cells (IC_50_ = 0.01 μM). To assess the toxicity and selectivity of the new compounds, their growth inhibitory activity was evaluated against normal MRC5 cells (IC_50_ = 1.27–24.06 μM). Compound **16c** showed the highest selectivity index against MCF7 and A2780 cells, while **16g** was the most selective for the HT29 cell line. To investigate the potential mechanism of action of the new compounds, **16g**, the most active in the MTT assay was evaluated for its effects on the cell cycle distribution of MCF7 cells. The results revealed cell cycle arrest at the G_2_/M phase. Compound **16g** also induced a dose-dependent increase in the apoptotic events in MCF7 cells compared to the control (7.9–17.8%). SwissTargetPrediction was used to predict the potential molecular targets which could mediate the anticancer potential of **16g**. The results revealed six potential targets including COX-2, MAP P38α, EGFR, CDK2, BRAF, and VEGFR1. A comparative molecular docking study of compound **16g** was performed into the six targets, where the results revealed high binding affinities for **16g** toward four of these targets (COX-2, MAP P38α, EGFR, CDK2). The molecular dynamic simulation also revealed favored stability and binding energy for **16g** in complex with CDK2 and EGFR, while COX-2 was in the third order. These findings suggested that compound **16g** could serve as a potential anticancer agent.

## Figures and Tables

**Table 1:** The top fifteen hits **1**–**15** of the compound library ranked based on RMSD, MWs, and RBs.

Hits	Code	RMSD *^a^*	MW *^b^*	RBs *^c^*
**1**	4och3pyr	0.753	427	7
**2**	4och3pyr	0.765	414	7
**3**	4och3pyr	0.769	419	6
**4**	4och3pyr	0.769	510	6
**5**	4och3pyr	0.769	398	6
**6**	4och3pyr	0.769	402	6
**7**	4och3pyr	0.769	463	6
**8**	8chpyr	0.777	419	6
**9**	10ipyr	0.777	510	6
**10**	3ch3pyr	0.777	398	6
**11**	9brpyr	0.777	463	6
**12**	7fpyr	0.777	402	6
**13**	10ipyr	0.780	510	6
**14**	9brpyr	0.780	463	6
**15**	7fpyr	0.780	402	6

*^a^* RMSD, root-mean square deviation; *^b^* MW, molecular weight; *^c^* RBs, number of rotatable bonds.

**Table 2:** Antiproliferative activity of compounds **10**, **16a**–**h**, and lapatinib against MCF-7, A2780, and HT29 cancer cell lines.

Comp.	IC_50_ (µM) *^a,b^*		
	MCF7	A2780	HT29
**16a**	40.50 ± 10.64	13.94 ± 1.92	0.19 ± 0.02
**16b**	0.08 ± 0.01	0.90 ± 0.08	10.22 ± 0.12
**16c**	0.03 ± 0.01	0.14 ± 0.02	2.27 ± 0.61
**16d**	7.05 ± 0.28	21.16 ± 2.43	0.17 ± 0.01
**16e**	0.15 ± 0.02	1.40 ± 0.21	0.34 ± 0.03
**16f**	40.45 ± 7.70	0.49 ± 0.12	0.21 ± 0.12
**16g**	0.01 ± 0.00	0.56 ± 0.01	0.37 ± 0.18
**16h**	26.95 ± 1.67	42.57 ± 3.47	0.71 ± 0.29
**10** *^c^*	0.33 ± 0.12	0.44 ± 0.01	0.41 ± 0.02
**Lapatinib**	5.98 ± 1.31	9.86 ± 1.72	13.22 ± 1.82

*^a^* IC_50_ is the concentration of test compounds which reduce cellular growth to 50% after treatment of cells with the test compounds for 72 h. *^b^* Results represent mean IC_50_ value ± S.D. (n = 3). *^c^* CI_50_ value of compound 10 quoted from our previous publication [20].

**Table 3:** Antiproliferative activity of compounds **10**, **16a**–**h**, and lapatinib against MRC5 cells.

Comp.	MRC5 (IC_50_ (µM) *^a,b^*)	Selectivity Index *^b^*		
		MCF7	A2780	HT29
**16a**	1.27 ± 0.48	0.03	0.09	6.68
**16b**	1.27 ± 0.32	15.88	1.41	0.12
**16c**	24.06 ± 1.31	802.00	171.86	10.60
**16d**	2.42 ± 0.56	0.34	0.11	14.24
**16e**	2.77 ± 0.09	18.47	1.98	8.15
**16f**	1.34 ± 0.45	0.03	2.73	6.38
**16g**	5.78 ± 0.63	578.00	10.32	15.62
**16h**	1.60 ± 0.12	0.06	0.04	2.25
**Lapatinib**	14.89 ± 2.45	2.49	1.51	1.13

*^a^* IC_50_ against MRC5 cells after 72 h treatment with the test compounds, results represent mean IC_50_ ± SD (*n* = 3). *^b^* Selectively index (SI) = IC_50_ value against the normal MRC5 cells divided by the IC_50_ value against the corresponding cancer cell line.

**Table 4:** Potential targets of compounds 16**a**–**h** based on the results of SwissTargetPrediction.

Comp.	Molecular Targets					
	COX-2	P38α	EGFR	CDK2	BRAF	VEGFR1
**16a**	+	+	+	+	−	-
**16b**	+	−	+	+	+	+
**16c**	+	+	+	+	−	+
**16d**	+	−	+	+	−	+
**16e**	+	+	+	+	+	+
**16f**	+	−	−	+	+	+
**16g**	+	+	+	+	+	+
**16h**	+	+	+	+	−	+
**10**	+	+	+	+	−	+

(+) indicated that the enzyme/kinase could be a potential target; (−) indicated that the target is not a potential target for the new compounds.

**Table 5:** The average distances of the formed hydrogen bond interactions in the six complexes.

Complex	Amino Acids Involved	Average Distance (Å) ± SD
**16g-COX2**	Arg106	3.52 ± 0.66
	Arg106	3.46 ± 0.51
	Tyr341	2.99 ± 0.72
	Ser516	2.83 ± 0.37
**16g-P38**	Lys53	2.71 ± 0.81
	Lys53	2.68 ± 0.56
	Met109	2.00 ± 0.39
	Leu171	3.01± 0.78
**16g-BRAF**	Cys532	3.1 ± 0.29
	Asn580	2.49 ± 0.5
**16g-VEGFR1**	Val892	3.02 ± 0.33
	Arg1021	2.41 ± 0.53
	Asp1040	2.15 ± 0.55
	Asp1040	3.22 ± 0.6
**16g-CDK2**	Leu83	1.98 ± 0.04
	Lys129	2.03 ± 0.11
	Asp145	2.28 ± 0.06
**16g-EGFR**	Cyc773	2.87 ± 0.20
	Asp831	1.88 ± 0.09

**Table 6:** MM-PBSA calculations of the binding free energy for the 16g in complex with the six potential targets.

Complex	ΔE_binding (kj/mol)_	ΔE_Electrostatic (kj/mol)_	ΔE*_Van der Waals’_* _(kj/mol)_	ΔE_polar solvation (kj/mol)_	SASA _(kJ/mol)_
**16g-CDK2**	−401 ± 20	−160 ± 17	−320 ± 28	107 ± 15	−28 ± 2
**16g-EGFR**	−387 ± 18	−155 ± 17	−306 ± 24	103 ± 14	−29 ± 2
**16g-COX-2**	−360 ± 14	−139 ± 13	−291 ± 20	95 ± 12	−25 ± 1
**16g-p38α**	−354 ± 17	−128 ± 14	−294 ± 24	92 ± 14	−26 ± 3
**16g-BRAF**	−337 ± 17	−126 ± 13	−280 ± 22	92 ± 15	−23 ± 1
**16g-VEGFR1**	−328 ± 15	−106 ± 12	−285 ± 22	85 ± 10	−22 ± 1

**Table 7:** Physicochemical properties and DLSs of compounds **16a**–**h**, and celecoxib.

Comp.	Physicochemical Properties					Lipinski’s Rule	%Abs *^d^*	BS	DLS
	MW *^a^*	TPSA *^b^*	ilogP *^c^*	H_A_	H_D_				
**16a**	427.50	82.65	3.58	4	1	Yes	80.49	0.55	0.25
**16b**	415.46	73.42	3.97	4	1	Yes	83.67	0.55	0.46
**16c**	398.46	79.41	3.62	4	1	Yes	81.60	0.55	0.44
**16d**	386.42	70.18	3.37	4	1	Yes	84.79	0.55	0.63
**16e**	402.42	79.41	3.94	5	1	Yes	81.60	0.55	0.77
**16f**	390.39	70.18	3.27	5	1	Yes	84.79	0.55	0.65
**16g**	418.88	79.41	3.82	4	1	Yes	81.60	0.55	0.95
**16h**	406.84	70.18	3.46	4	1	Yes	84.79	0.55	0.76
**10**	402.88	70.18	3.48	3	1	Yes	84.79	0.55	0.80

*^a^* MW, Molecular weight (Da); *^b^* TPSA, topological polar surface area; *^c^* ilogP, in-house physics based logP; *^d^* %Abs, % absorbed orally, *^e^* %Abs = 109 − (0.345 × TPSA).

## Data Availability

The supplementary materials were provided with the manuscript “were uploaded with the first and revised submission”. You can use the supplementary material file uploaded to the system with the revised manuscript.

## Supplementary Materials

Supplementary material including spectral data (IR, Mass, ^1^H-NMR, ^13^C-NMR, and DEPT C^135^ spectra) of compounds **16a**–**h** are provided with this manuscript (Figures S1–S61).

## Sample Availability

Samples of the compounds are available from the authors.
