# Supplementary material for: In Silico Approach Using Free Software to Optimize the Antiproliferative Activity and Predict the Potential Mechanism of Action of Pyrrolizine-Based Schiff Bases
Source: Molecules. 2021 Jun 30;26(13):4002. doi: 10.3390/molecules26134002 (PMC8271847; doi:10.3390/molecules26134002)

In silico approach using free software to optimize the antiproliferative activity and predict the potential mechanism of action of pyrrolizine-based Schiff bases

Faisal A. Almalki[1], Ashraf N. Abdalla[2,3], Ahmed M. Shawky[4], Mahmoud A. El Hassab[5], Ahmed M. Gouda[1,6,*]

[1]Department of Pharmaceutical Chemistry, Faculty of Pharmacy, Umm Al-Qura University, Makkah 21955, Saudi Arabia

[2]Department of Pharmacology and Toxicology, Faculty of Pharmacy, Umm Al-Qura University, Makkah 21955, Saudi Arabia

[3]Departmentof Pharmacology and Toxicology, Medicinal and Aromatic plants research institute, National center for research, Khartoum 2404, Sudan

[4]Science and Technology Unit (STU), Umm Al-Qura University, Makkah 21955, Saudi Arabia

[5] Department of Pharmaceutical Chemistry, School of Pharmacy, Badr University in Cairo (BUC), Cairo 11829, Egypt

[6]Medicinal Chemistry Department, Faculty of pharmacy, Beni-Suef University, Beni-Suef 62514, Egypt

***Correspondence: Ahmed M. Gouda:** Department of Medicinal Chemistry, Faculty of Pharmacy, Beni-Suef University, Beni-Suef 62514, Egypt.

Scopus ID: 26321547200

ORCID: 0000-0003-4527-8885

Tel.: (002)-01126897483

Fax: (002)-082-2162133

E-mail address: ahmed.gouda@pharm.bsu.edu.eg or amsaid@uqu.edu.sa

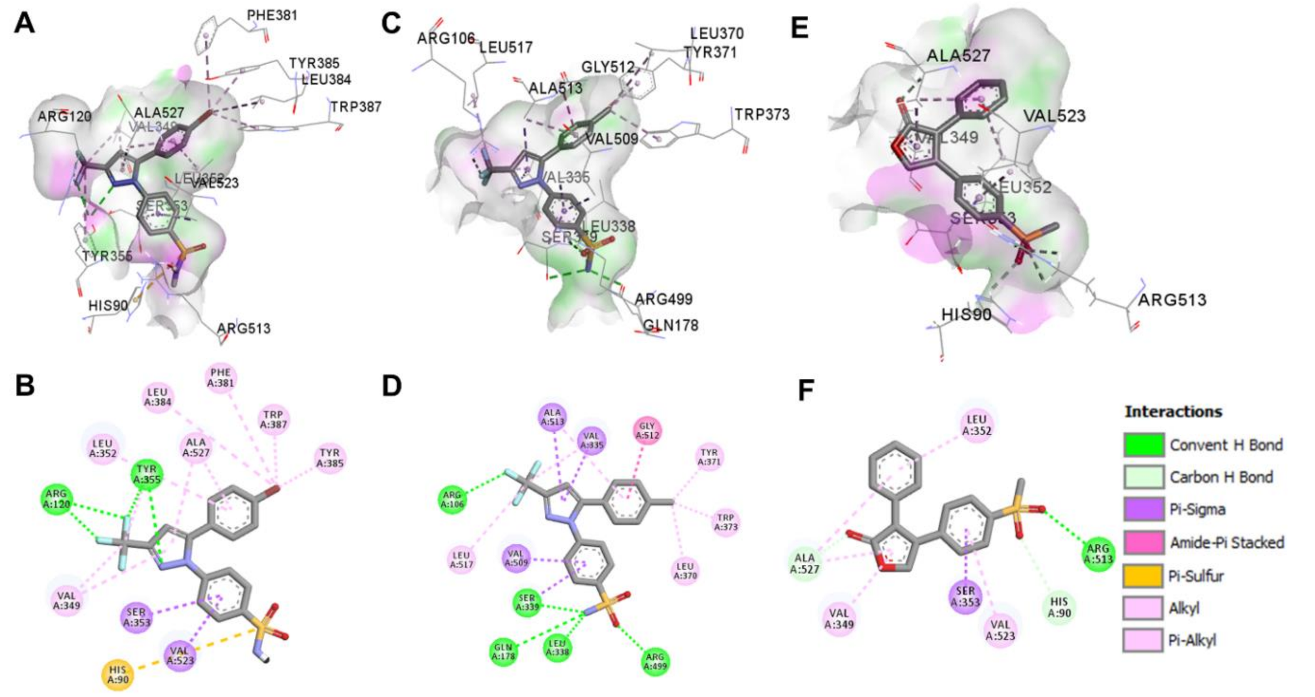

Figure S1. Binding mode/interactions of SC-558 **1** (pdb: 1CX2), celecoxib **2** (pdb: 3LN1), and rofecoxib **3** (pdb: 5KIR): A) 3D binding mode of SC-558 **1** (showed as sticks) colored by element; B) 2D binding mode of SC-558 **1** showing different types of interaction with amino acids in COX-2; C) 3D binding mode of celecoxib (showed as sticks) colored by element; D) 2D binding mode of celecoxib showing different types of interaction with amino acids in COX-2; E) 3D binding mode of rofecoxib (showed as sticks) colored by element; F) 2D binding mode of rofecoxib showing different types of interaction with amino acids in COX-2.

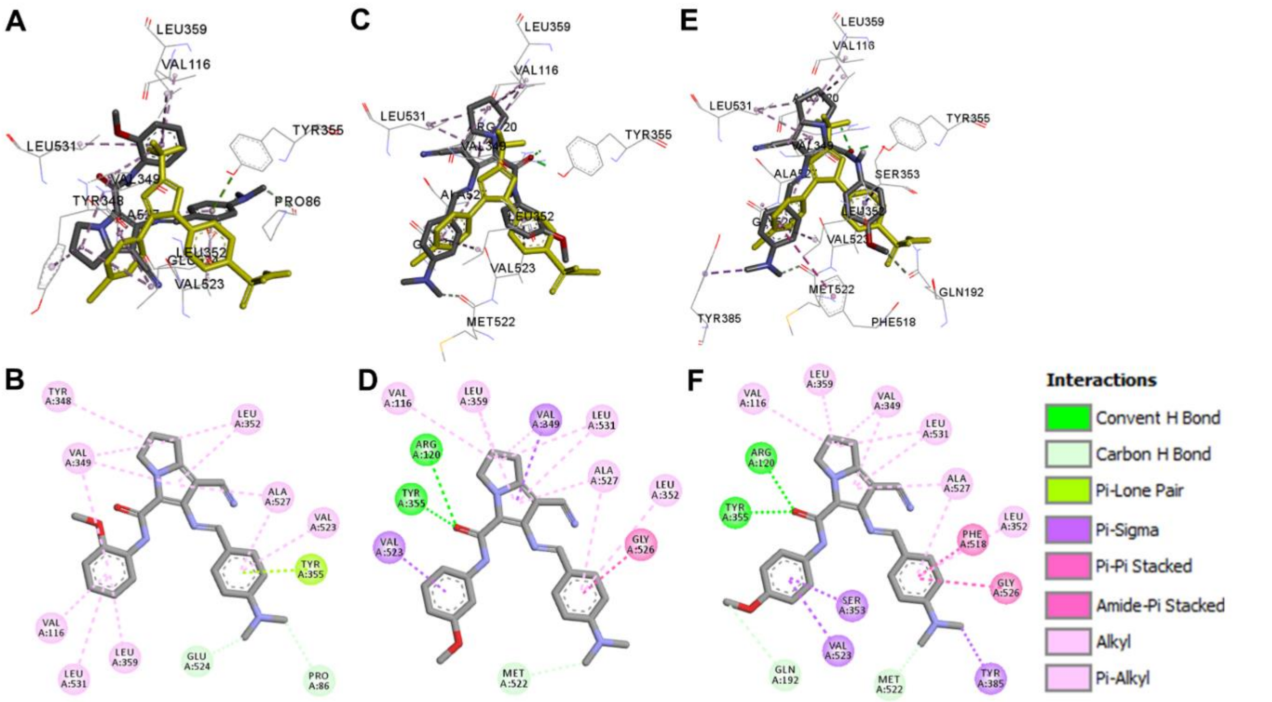

Figure S2. Binding mode/interactions of hit **1*o,m,p*** (shown as sticks colored by element) into COX-2 (pdb: 1CX2): A) 3D binding mode of **1*o*** into COX-2 overlaid with SC-558 (yellow sticks); B) 2D binding mode of **1*o*** into COX-2 showing different interactions; C) 3D binding mode of **1*m*** into COX-2 overlaid with SC-558 (yellow sticks); D) 2D binding mode of **1*m*** into COX-2 showing different interactions; E) 3D binding mode of **1*p*** into COX-2 overlaid with SC-558 (yellow sticks); F) 2D binding mode of **1*p*** into COX-2 showing different interactions.

IR Spectra of the new compounds

Infrared spectra (IR) were done using BRUKER TENSOR 37 spectrophotometer and absorption were expressed in wave number (cm^{-1}) using KBr Disk.

Figure S3. **IR spectrum of compound 16a**

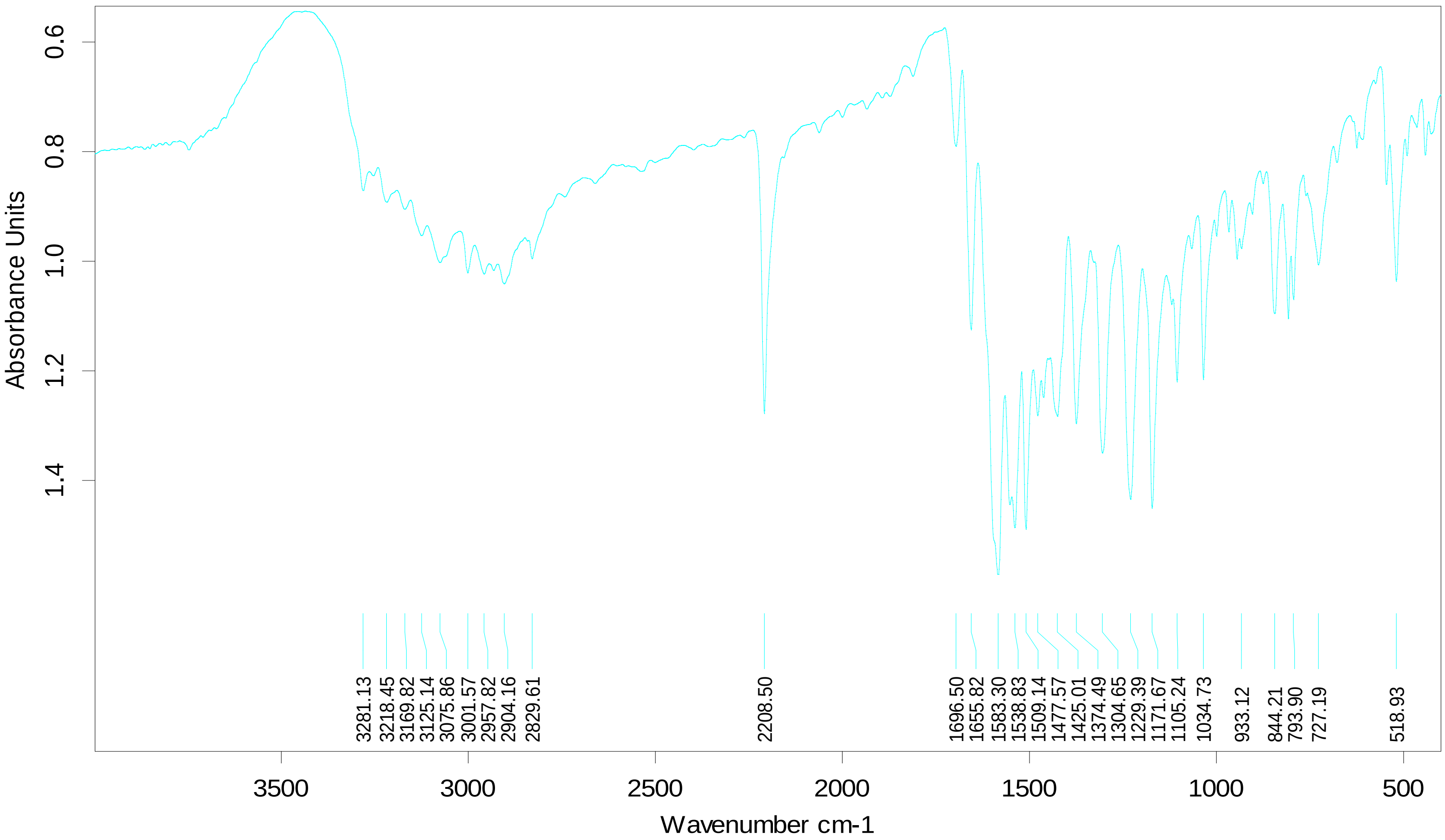

C:\Program Files\OPUS_65\MEAS\Protein.242 Protein AquaSpec	31/01/2018

Page 1/1

Figure S4. IR spectrum of compound **16b**

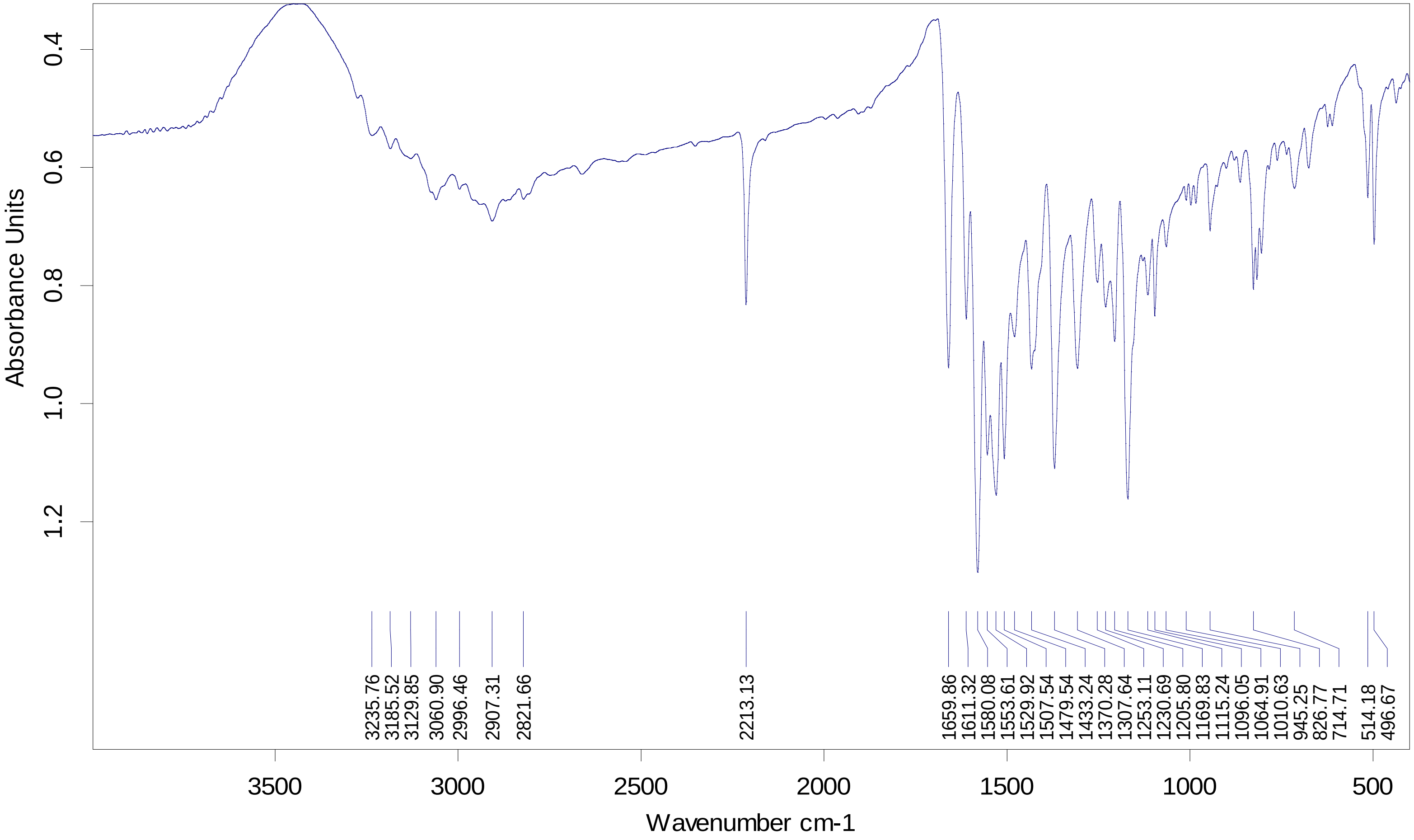

C:\Program Files\OPUS_65\MEAS\Protein.246	Protein	AquaSpec		31/01/2018

Page 1/1

Figure S5. **IR spectrum of compound 16c**

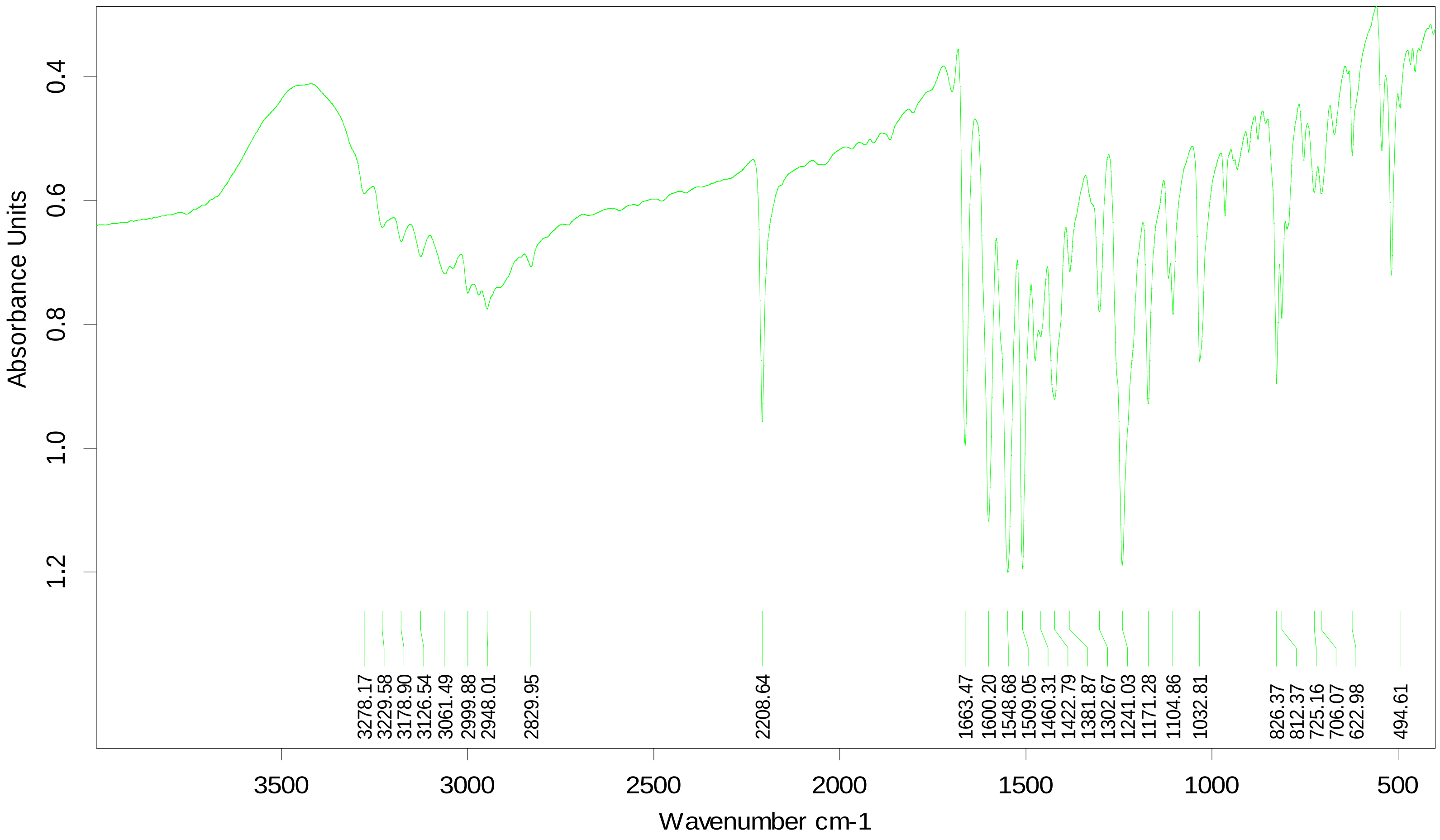

C:\Program Files\OPUS_65\MEAS\Protein.241 Protein AquaSpec	31/01/2018

Page 1/1

Figure S6. IR spectrum of compound **16d**

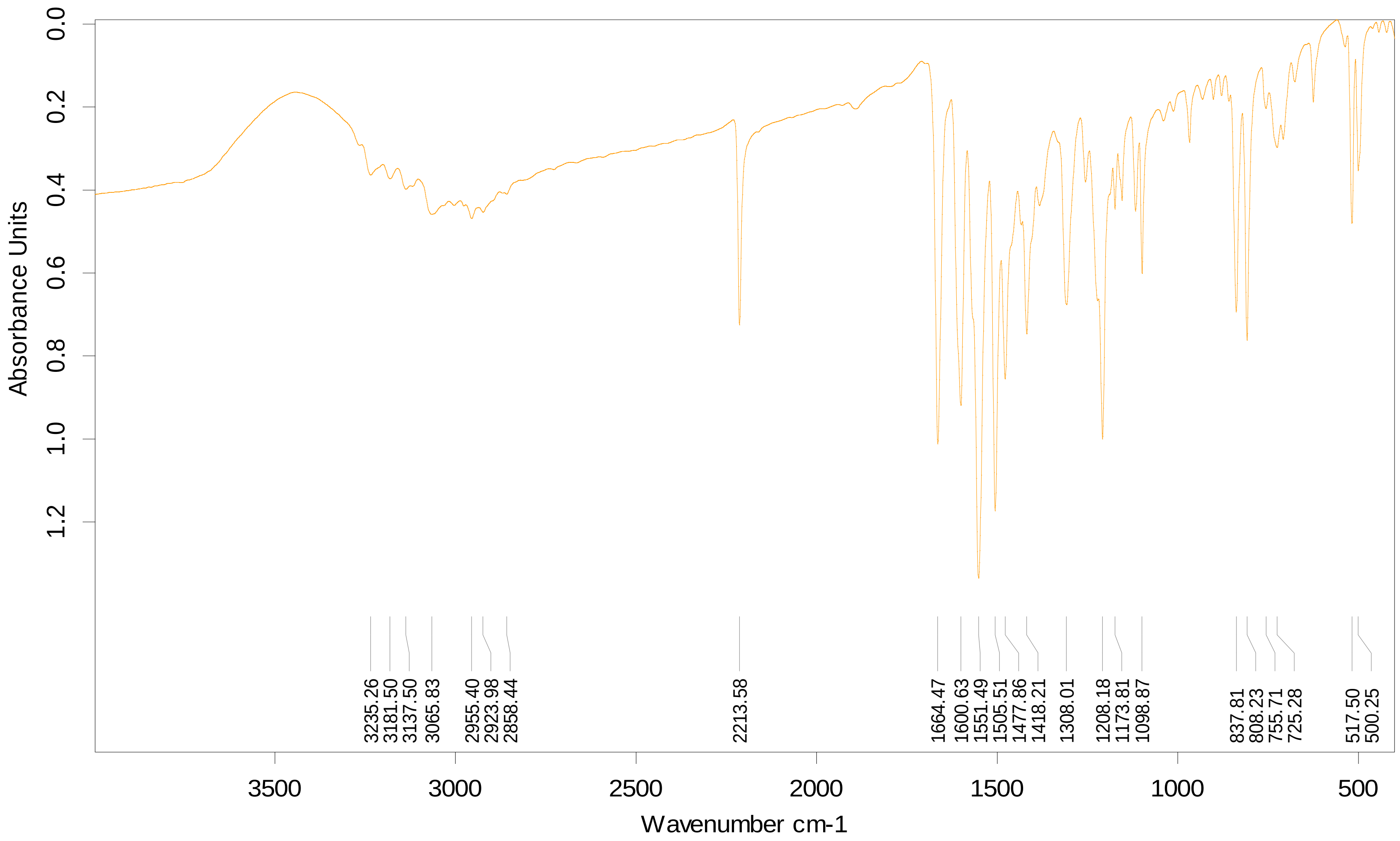

C:\Program Files\OPUS_65\MEAS\Protein.245	Protein	AquaSpec	31/01/2018

Page 1/1

Figure S7. IR spectrum of compound **16e**

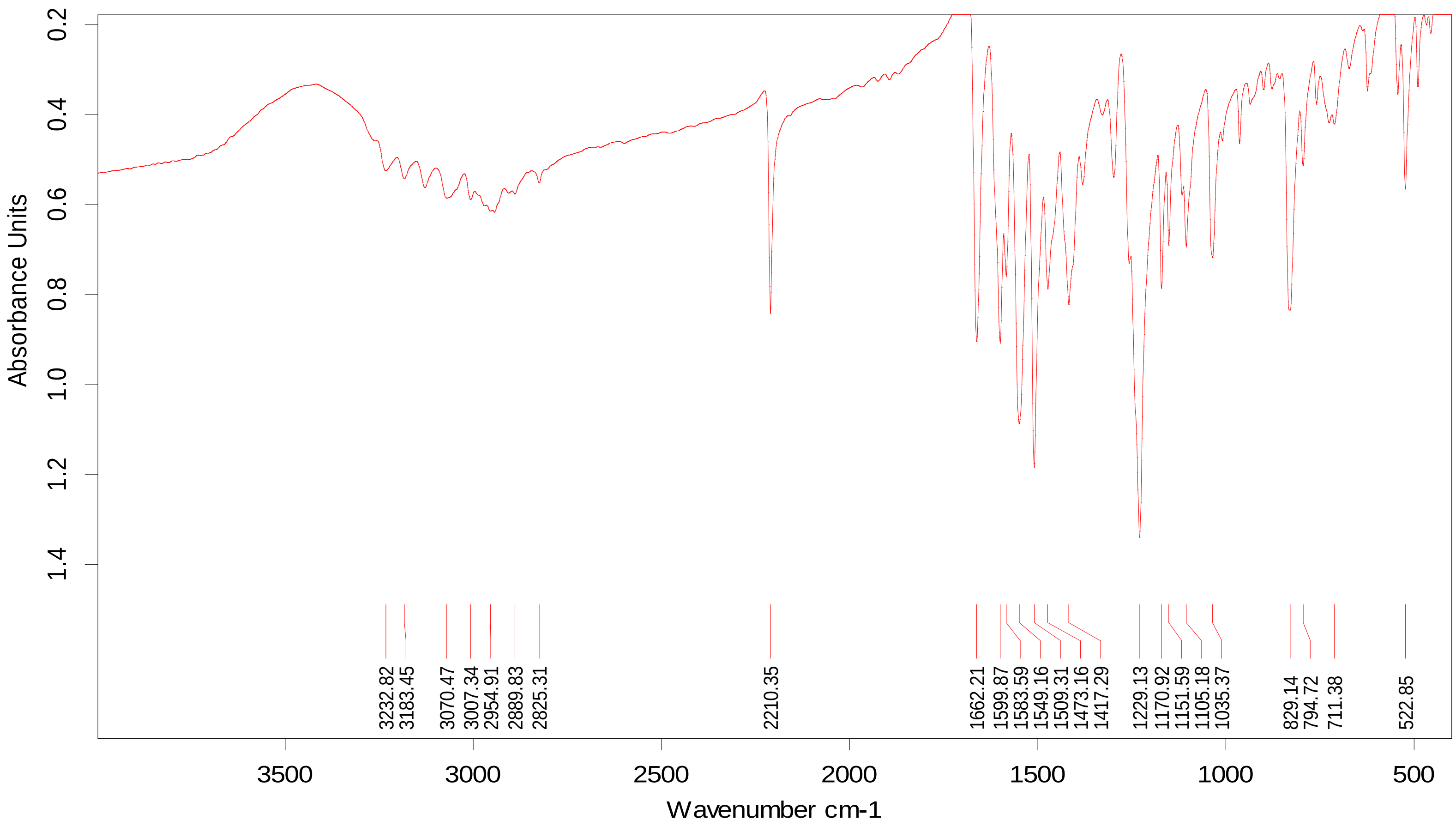

C:\Program Files\OPUS_65\MEAS\Protein.239 Protein AquaSpec	31/01/2018

Page 1/1

Figure S8. IR spectrum of compound **16f**

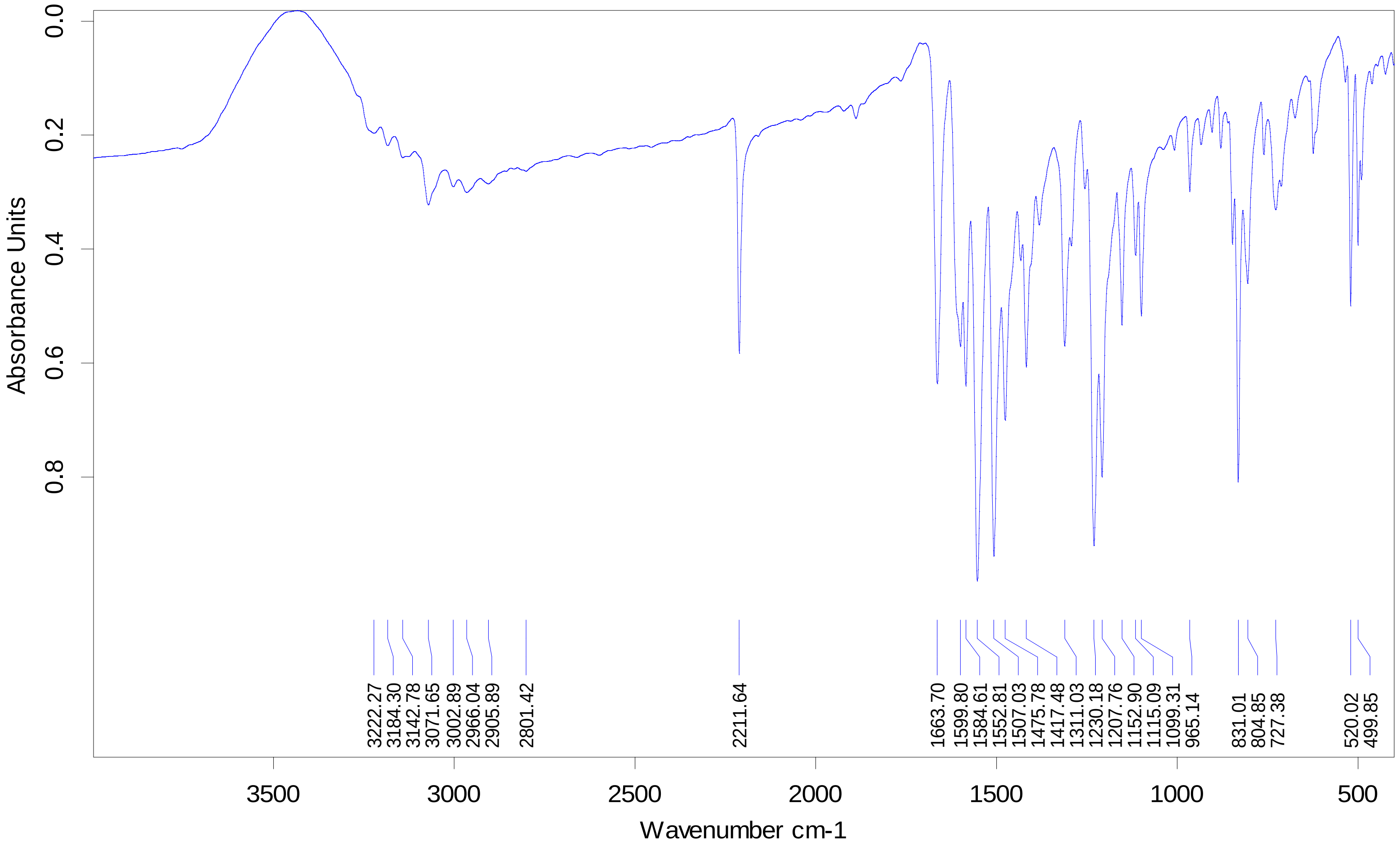

C:\Program Files\OPUS_65\MEAS\Protein.243	Protein	AquaSpec		31/01/2018

Page 1/1

Figure S9. IR spectrum of compound **16g**

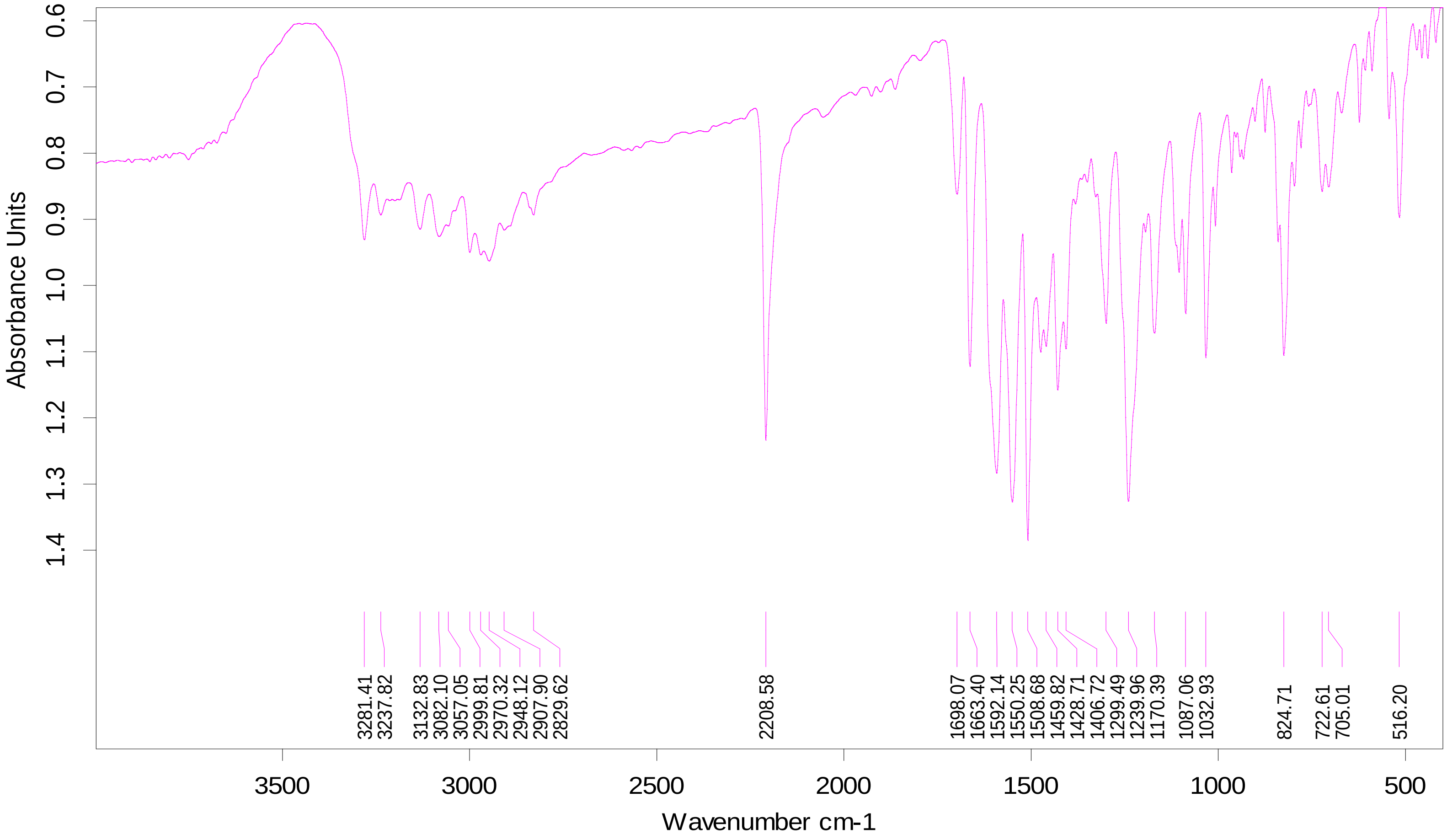

C:\Program Files\OPUS_65\MEAS\Protein.240	Protein	AquaSpec	31/01/2018

Page 1/1

Figure S10. **IR spectrum of compound 16h**

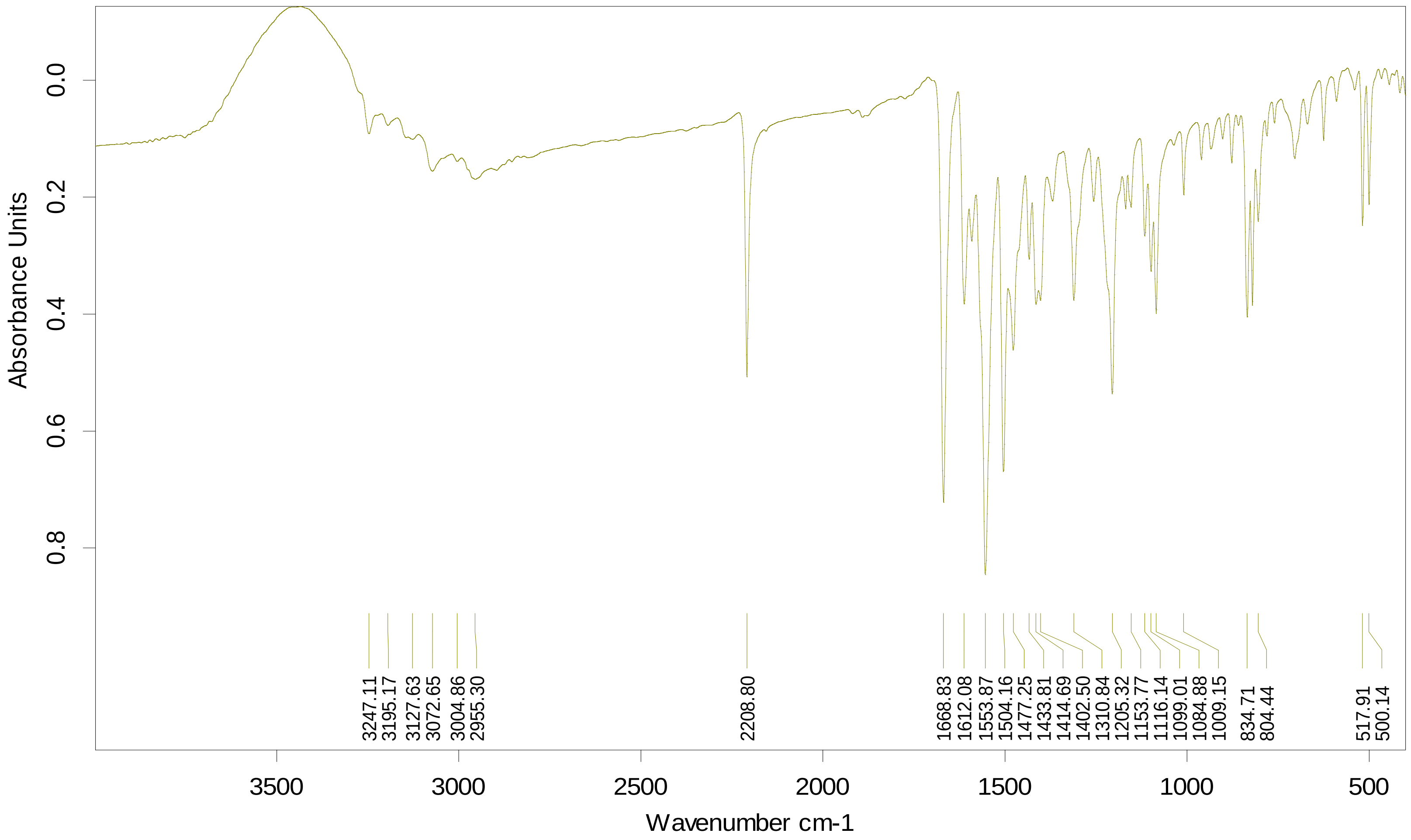

C:\Program Files\OPUS_65\MEAS\Protein.244 Protein AquaSpec	31/01/2018

Page 1/1

^{1}H-NMR, ^{13}C-NMR and DEPT C^{135} Spectra

^{1}H-NMR spectra were recorded on a BRUKER AVANCE III spectrometer (at the faculty of pharmacy, Umm Al-Qura University) at 500 MHz in the specified solvent, chemical shifts were reported on the δ (ppm) scale and were related to that of the solvent and J values are given in Hz. ^{13}C NMR and DEPT C^{135} spectra were obtained on a BRUKER AVANCE III at 125 MHz (at the faculty of pharmacy, Umm Al-Qura University).

Figure S11. ^{1}H-NMR ($CDCl_3$, 500 MHz, δ ppm) spectrum of compound **16a**

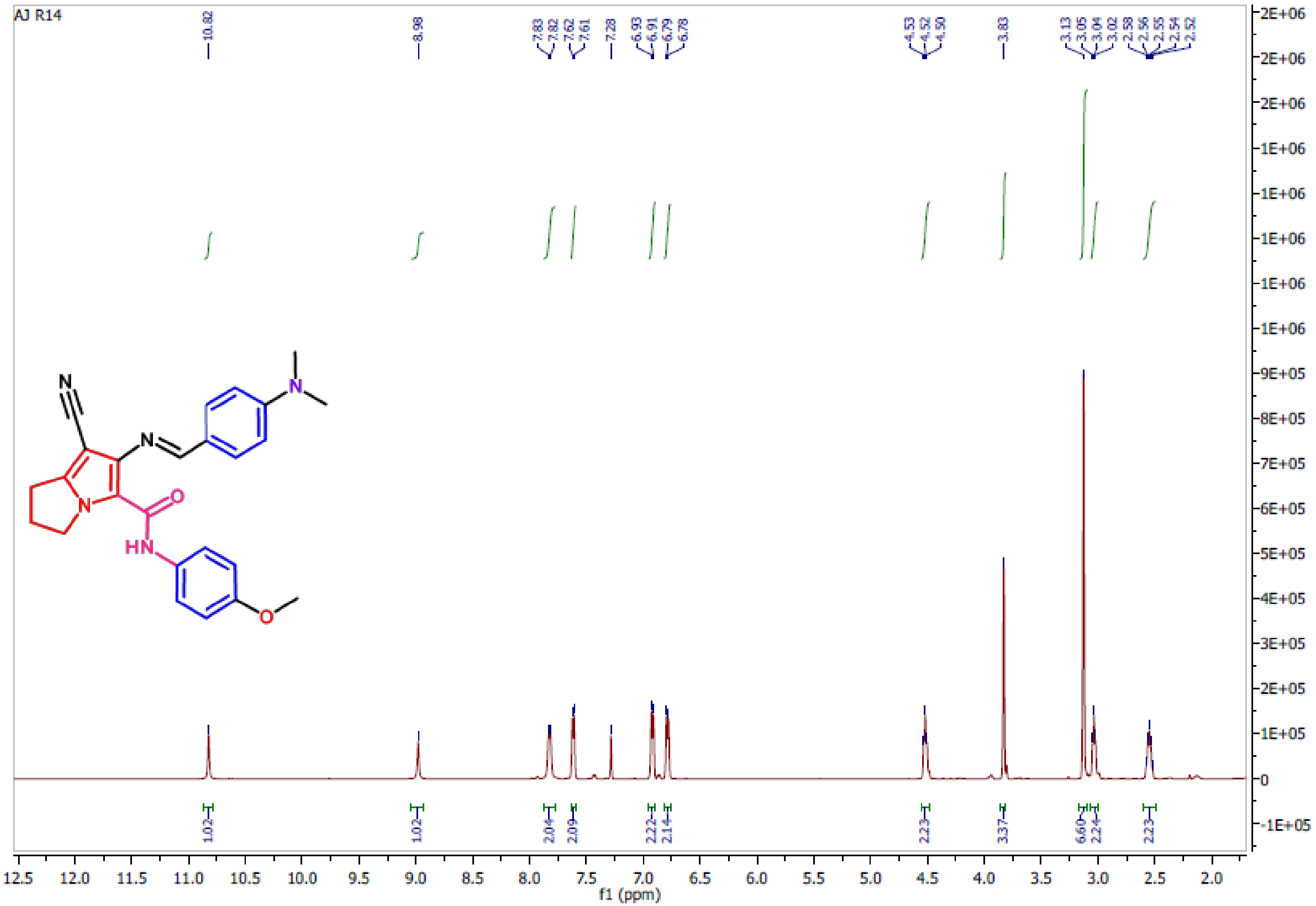

Figure S12. ^{13}C-NMR ($CDCl_3$, 125 MHz, δ ppm) spectrum of compound **16a**

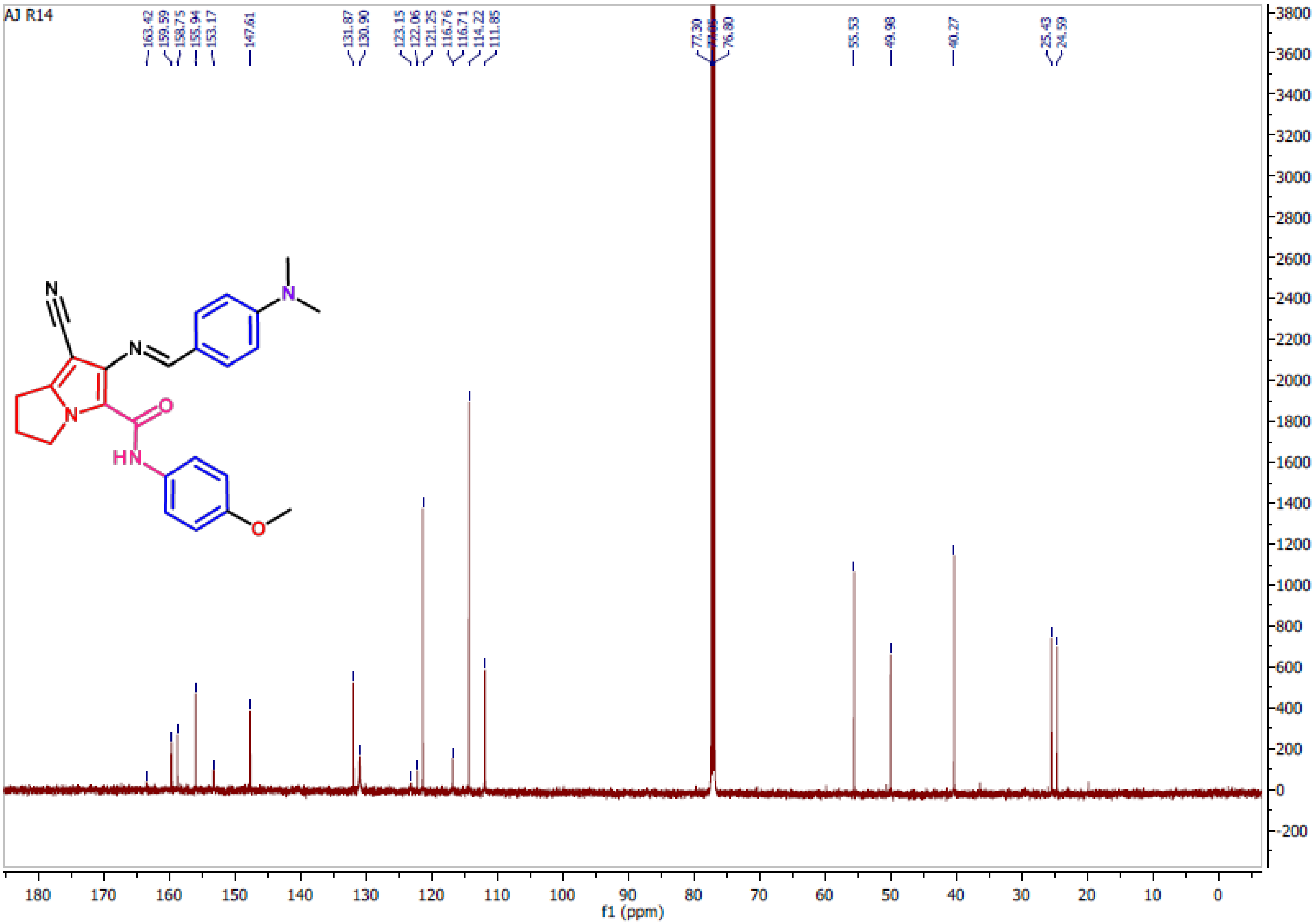

Figure S13. **DEPT C[135] (CDCl$_3$, 125 MHz, δ ppm) spectrum of compound 16a (CDCl$_3$)**

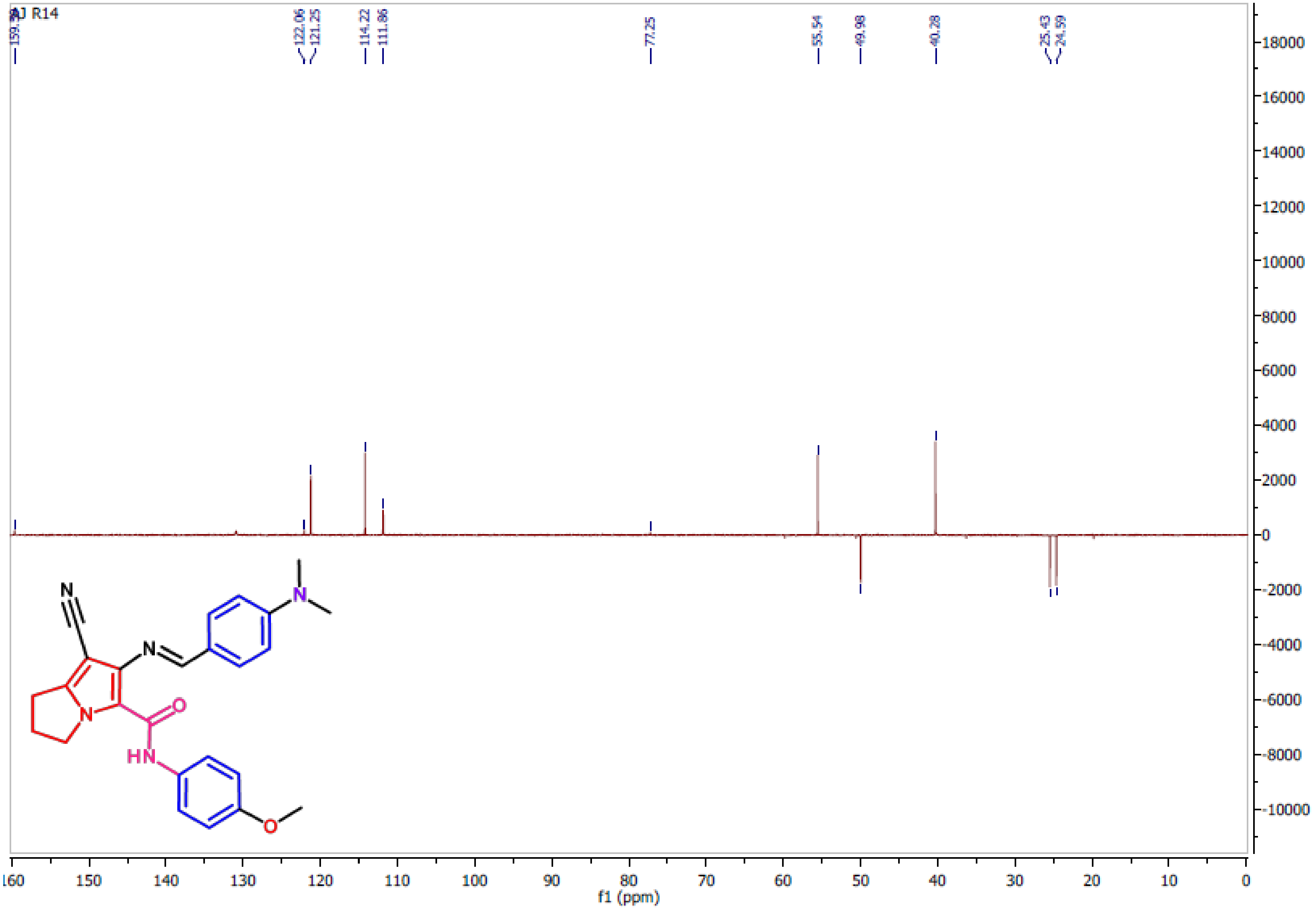

Figure S14. ^{1}H-NMR ($CDCl_3$, 500 MHz, δ ppm) spectrum of compound **16b**

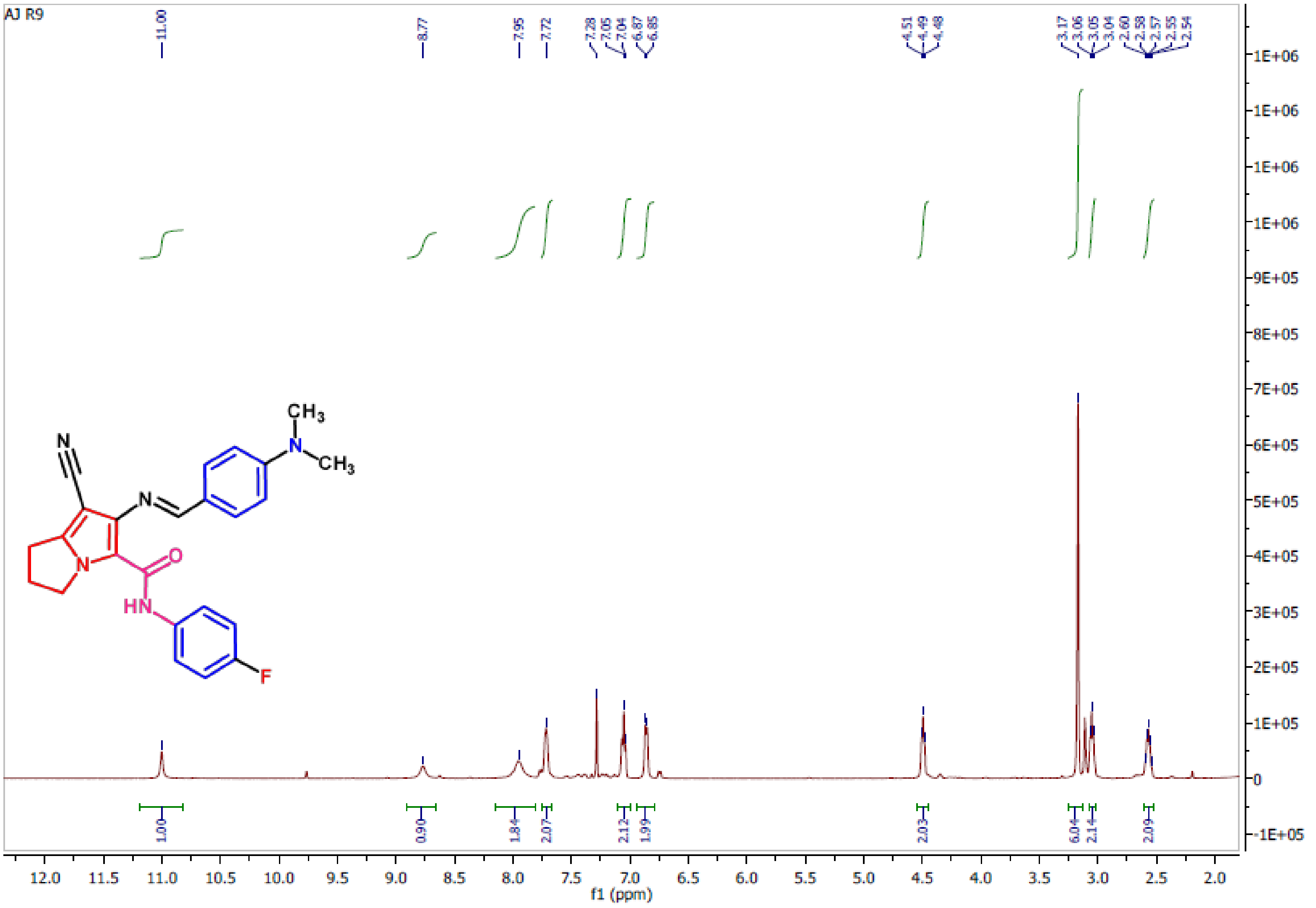

Figure S15. ^{13}C-NMR ($CDCl_3$, 125 MHz, δ ppm) spectrum of compound **16b**

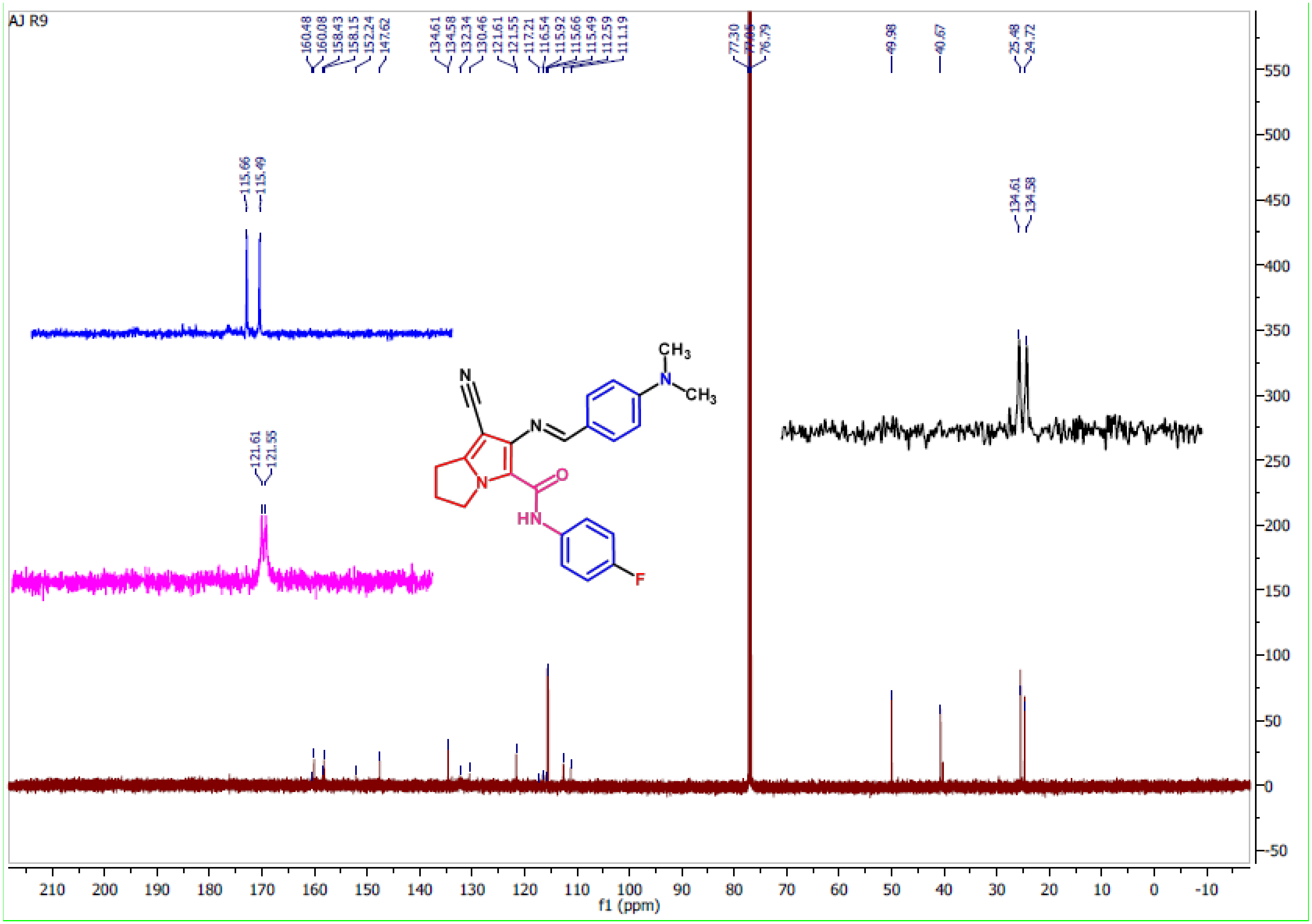

Figure S16. **DEPT C[135] (CDCl₃, 125 MHz, *δ* ppm) spectrum of compound 16b**

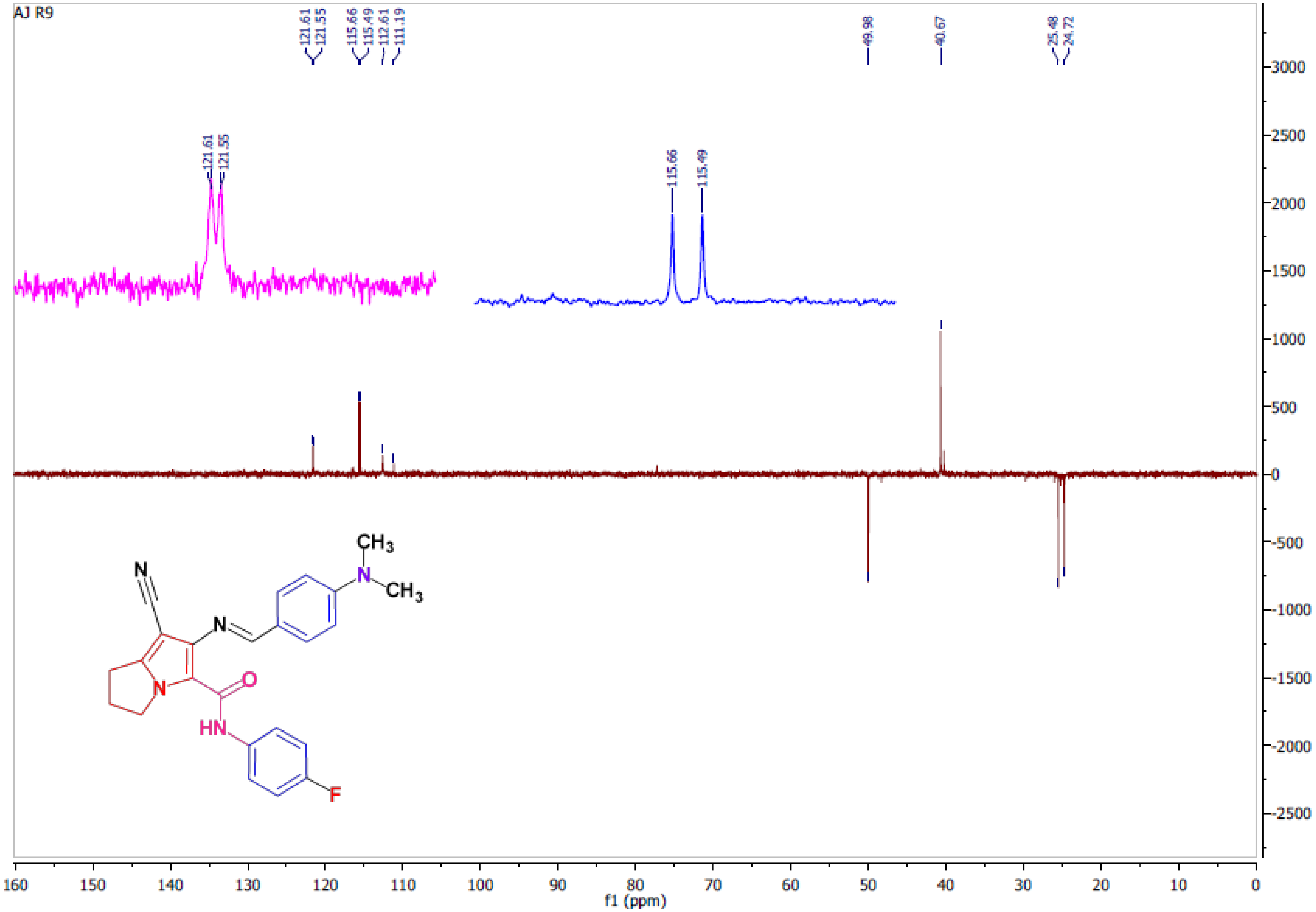

Figure S17. ^{1}H-NMR ($CDCl_3$, 500 MHz, *δ* ppm) spectrum of compound **16c**

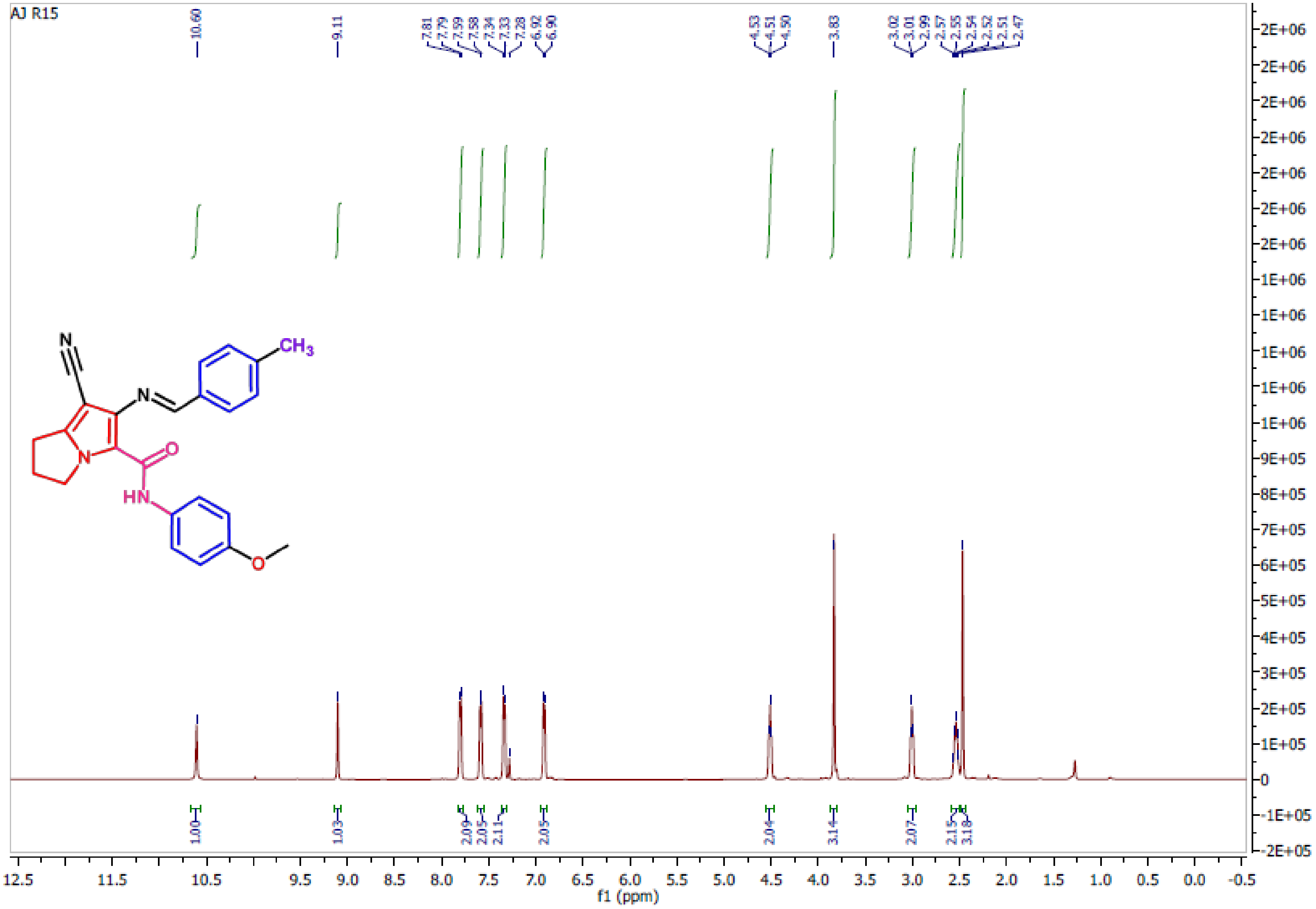

Figure S18. ^{13}C-NMR ($CDCl_3$, 125 MHz, δ ppm) spectrum of compound 16c

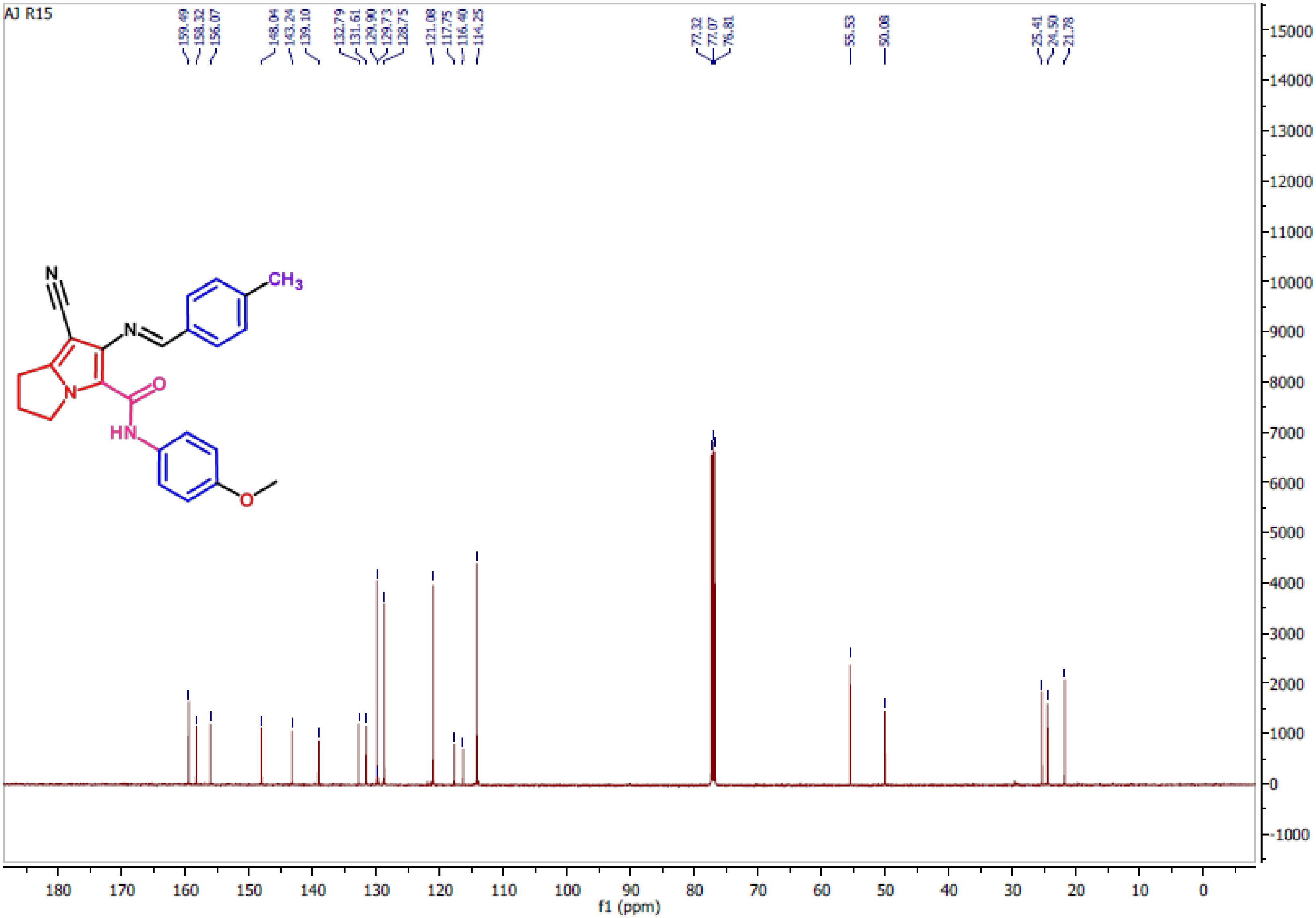

Figure S19. **DEPT C^{135} ($CDCl_3$, 125 MHz, δ ppm) spectrum of compound 16c**

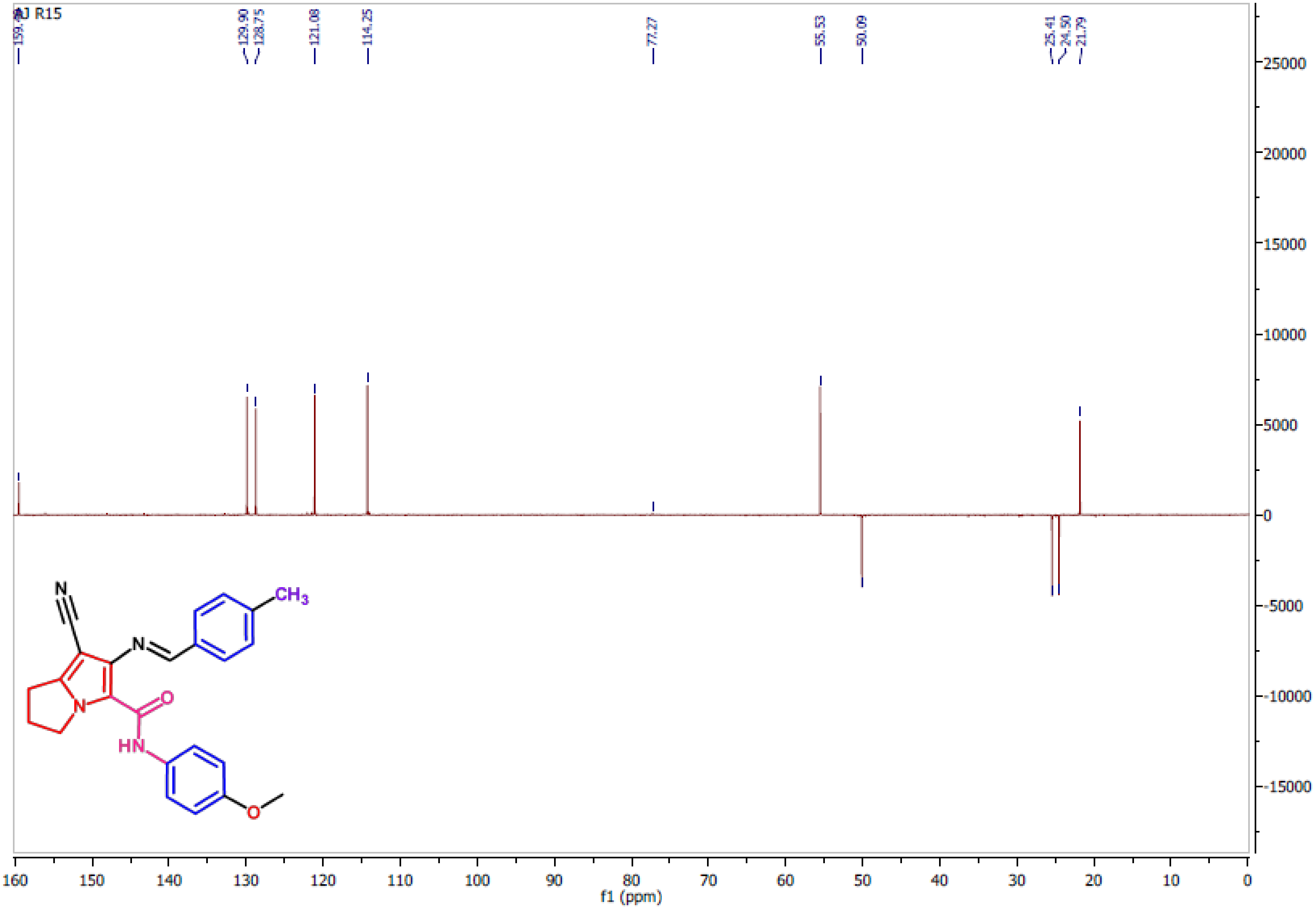

Figure S20. ^{1}H-NMR ($CDCl_3$, 500 MHz, δ ppm) spectrum of compound **16d**

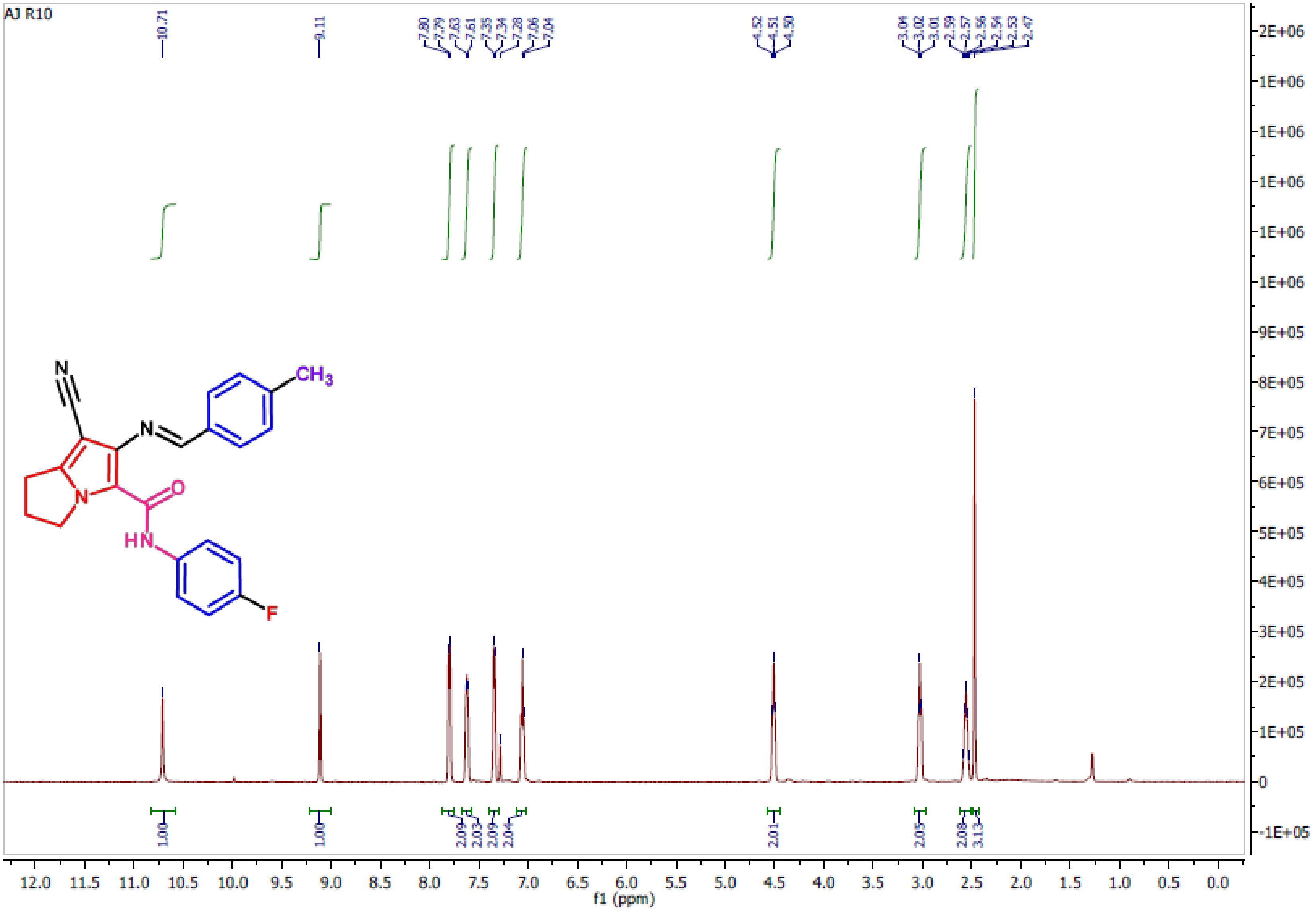

Figure S21. ^{13}C-NMR ($CDCl_3$, 125 MHz, *δ* ppm) spectrum of compound **16d**

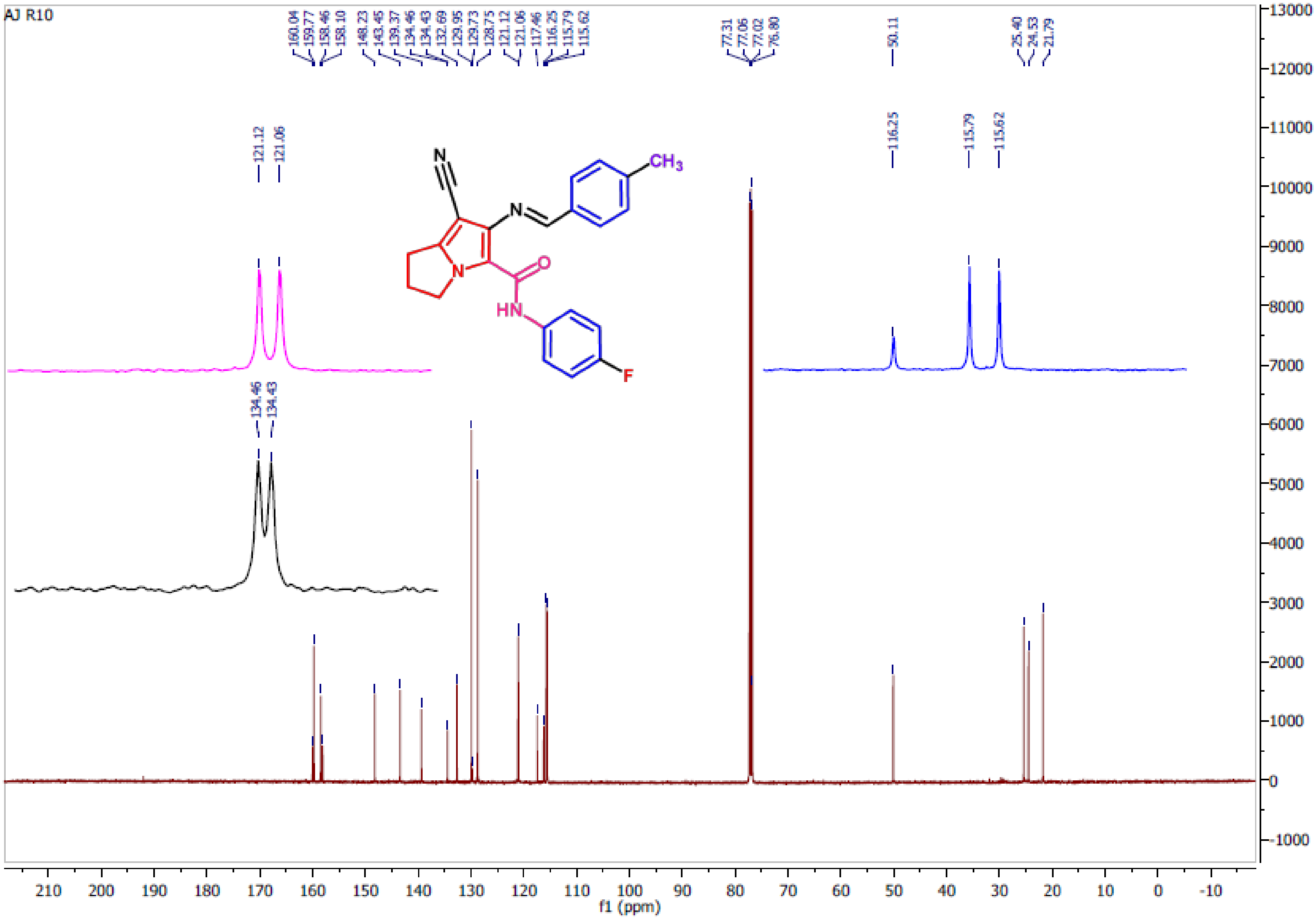

Figure S22. DEPT C^{135} ($CDCl_3$, 125 MHz, δ ppm) spectrum of compound **16d**

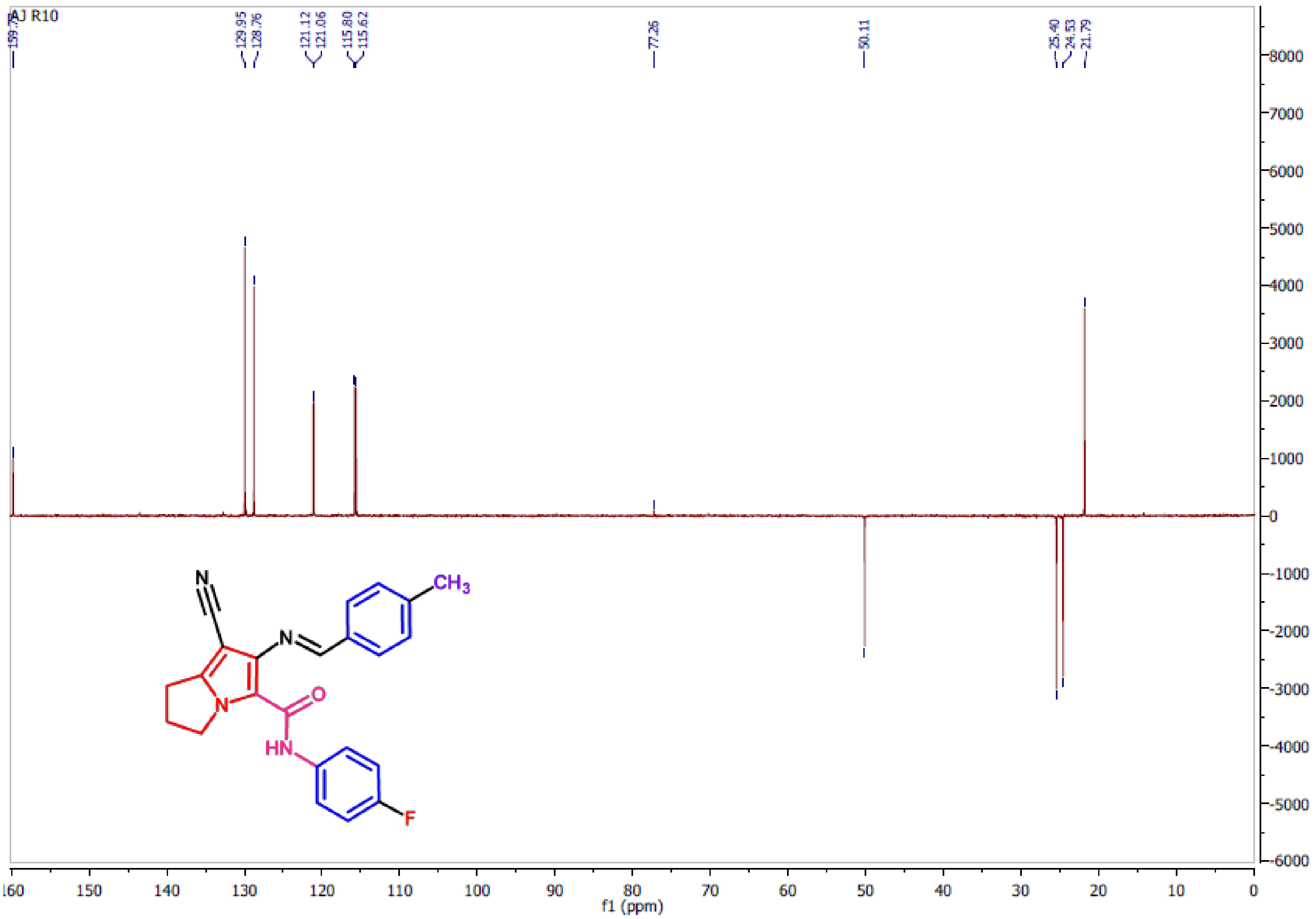

Figure S23. **DEPT C^{135} ($CDCl_3$, 125 MHz, δ ppm) spectrum of compound 16d**

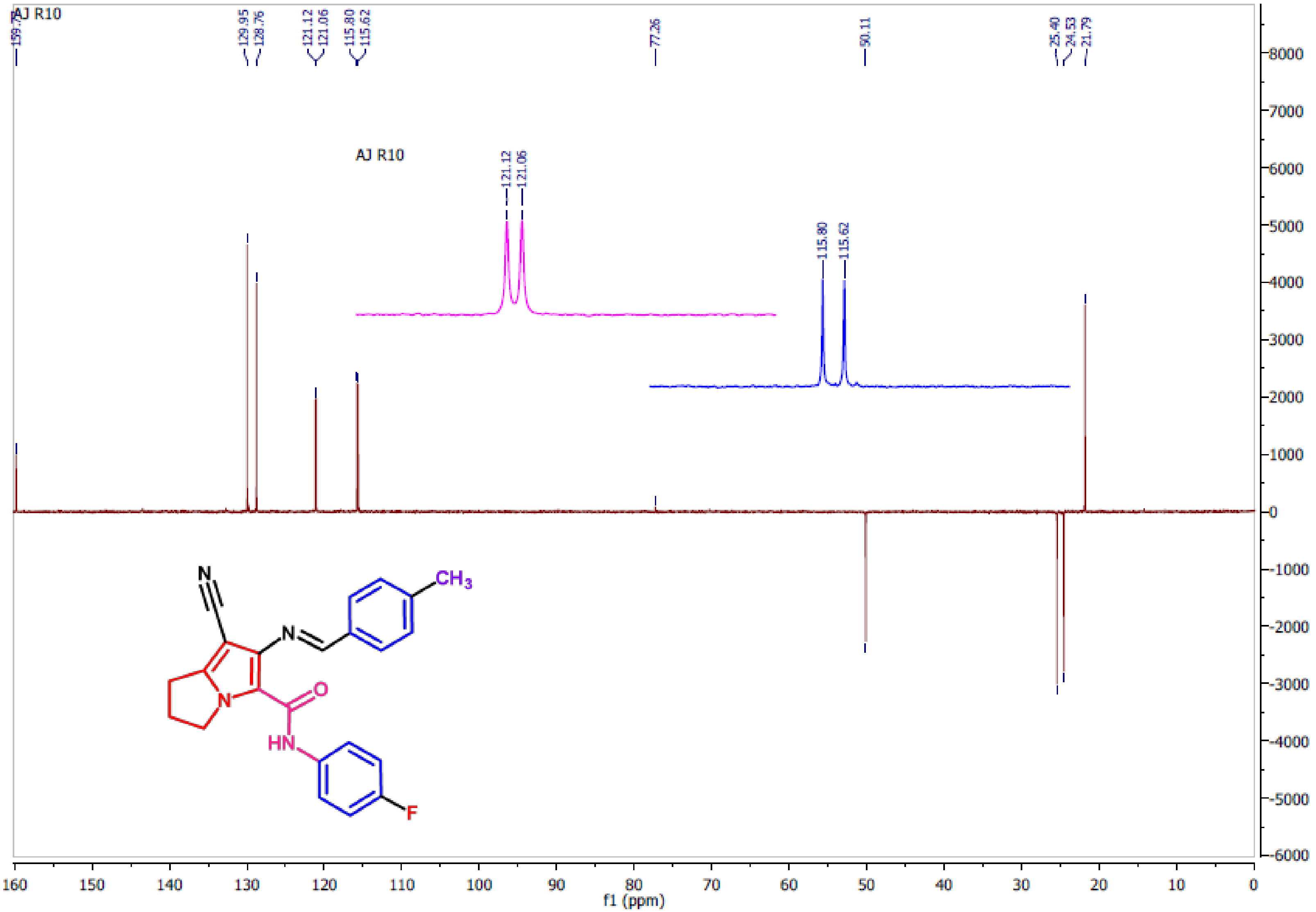

Figure S24. ^{1}H-NMR ($CDCl_3$, 500 MHz, δ ppm) spectrum of compound **16e**

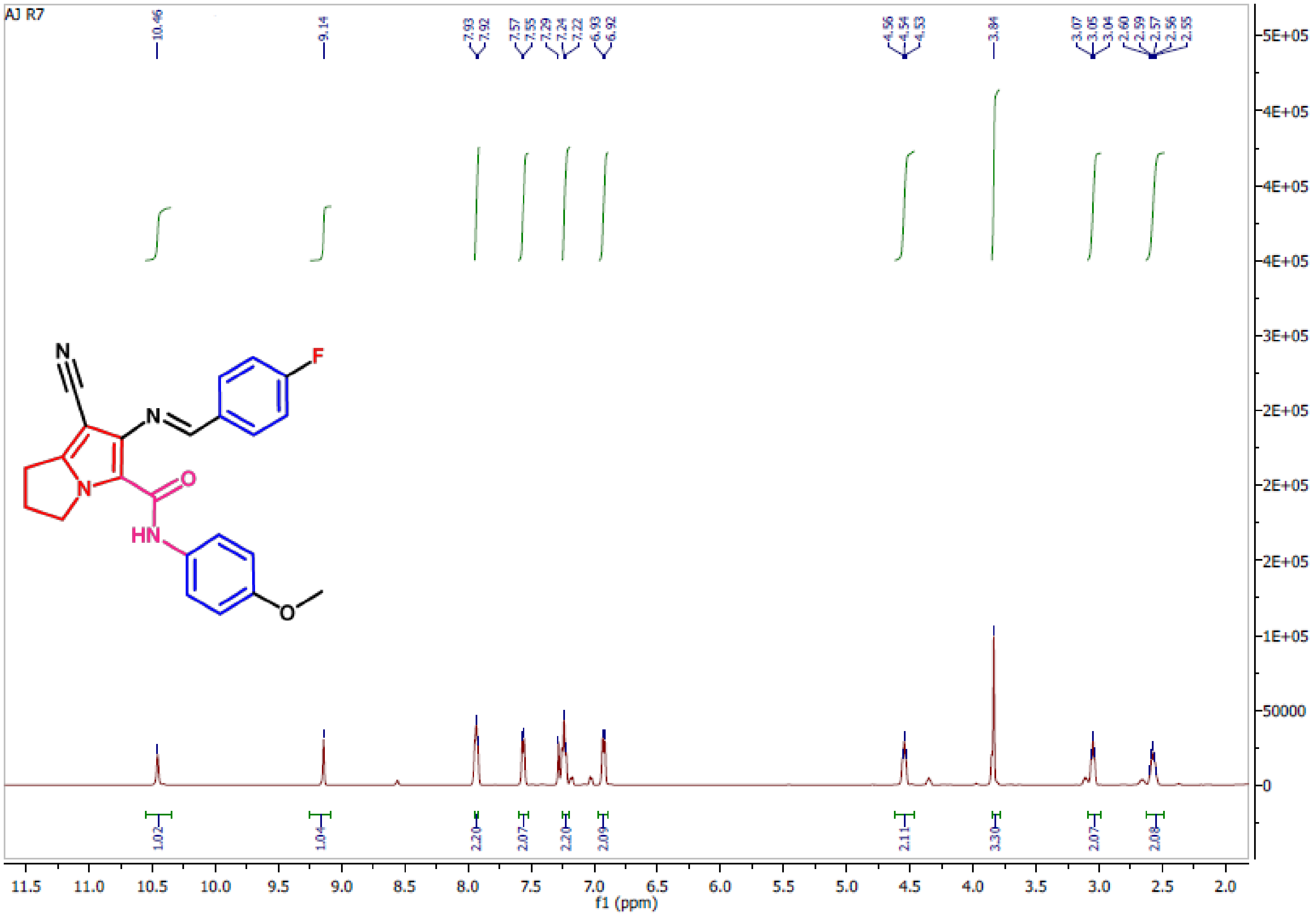

Figure S25. ^{13}C-NMR (CDCl$_3$, 125 MHz, δ ppm) spectrum of compound **16e**

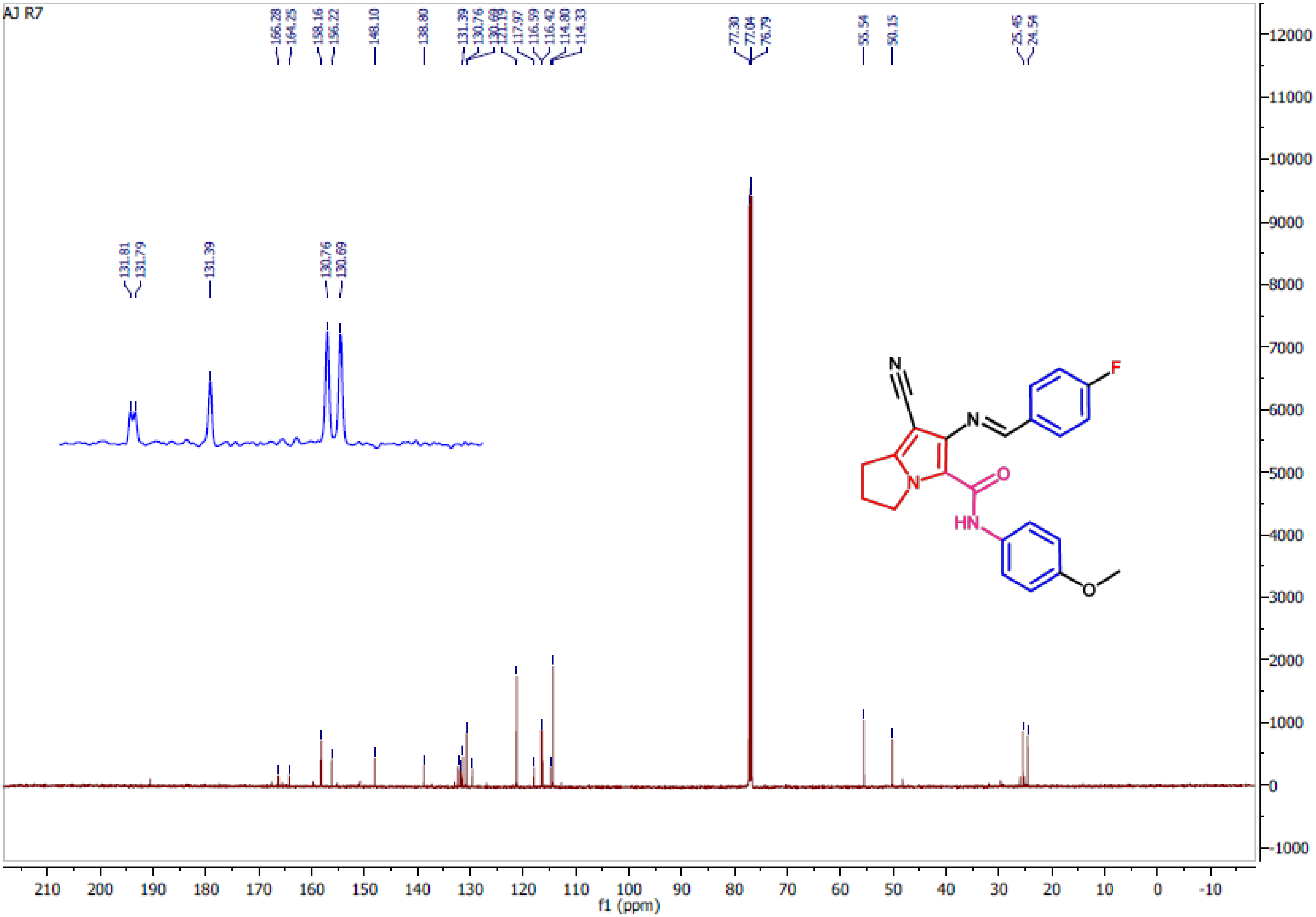

Figure S26. **DEPT C^{135} ($CDCl_3$, 125 MHz, δ ppm) spectrum of compound 16e**

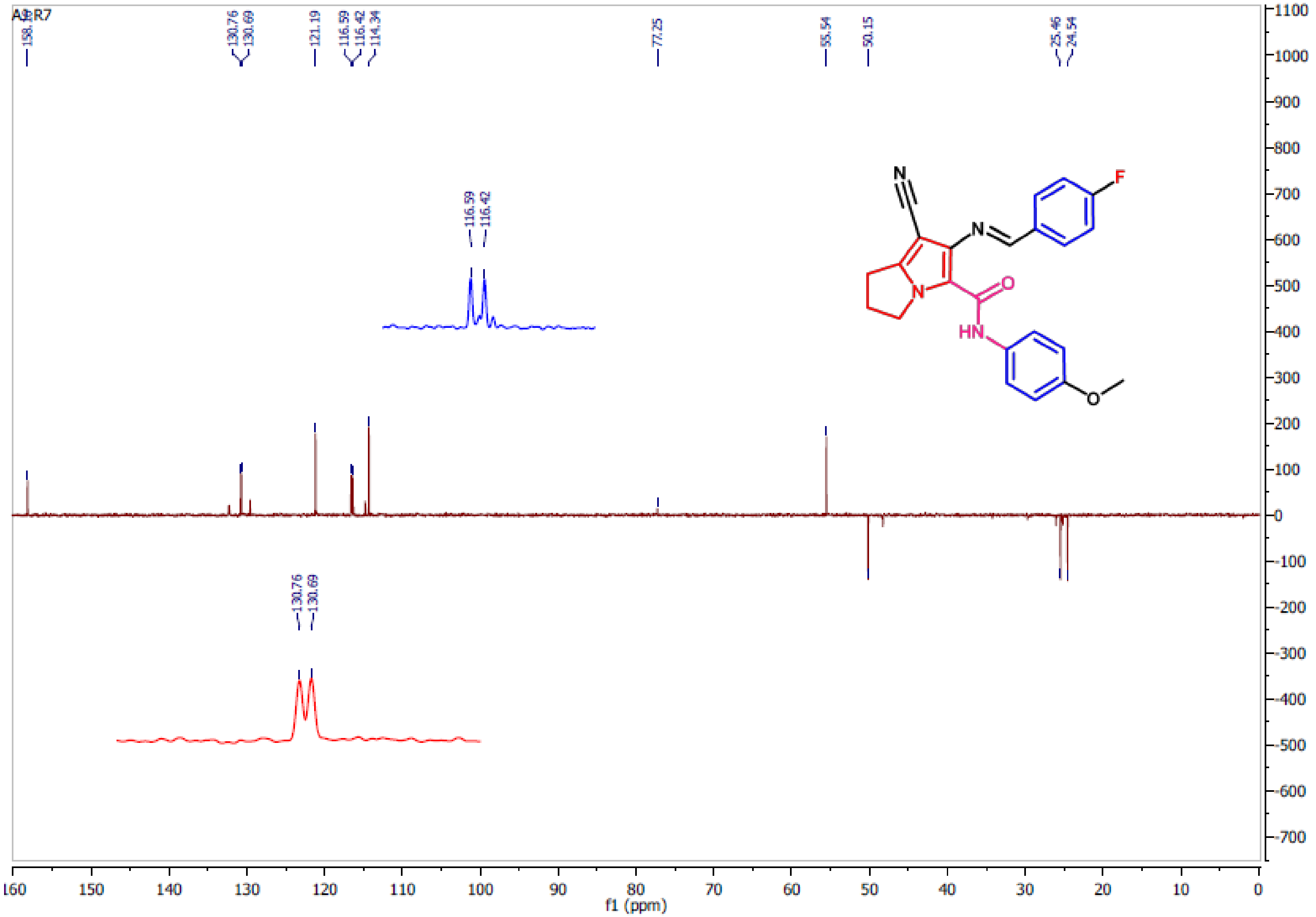

Figure S27. ^{1}H-NMR ($CDCl_3$, 500 MHz, δ ppm) spectrum of compound **16f**

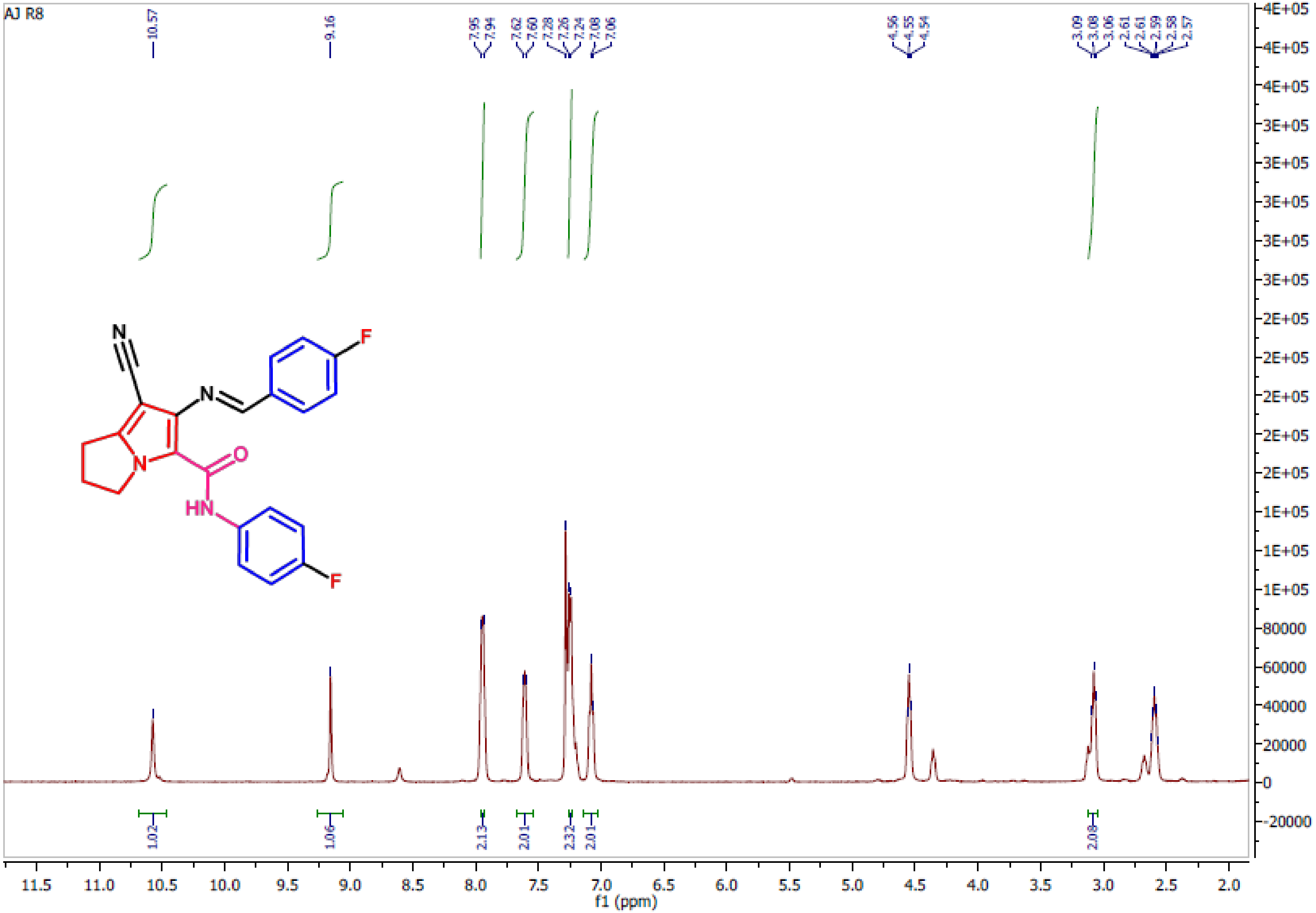

Figure S28. ^{13}C-NMR ($CDCl_3$, 125 MHz, δ ppm) spectrum of compound **16f**

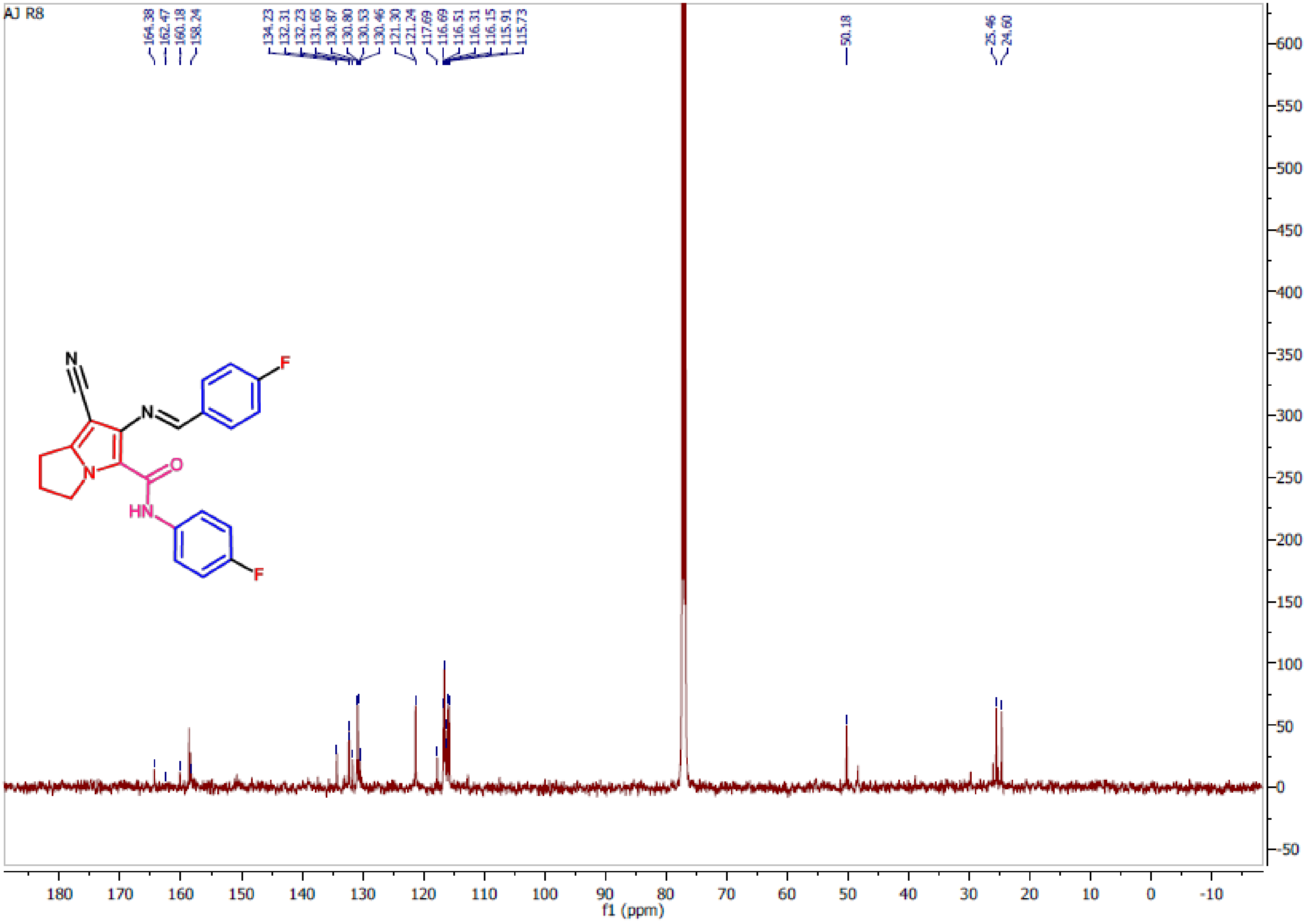

Figure S29. ^{13}C-NMR (CDCl$_3$, 125 MHz, δ ppm) spectrum of compound **16f**

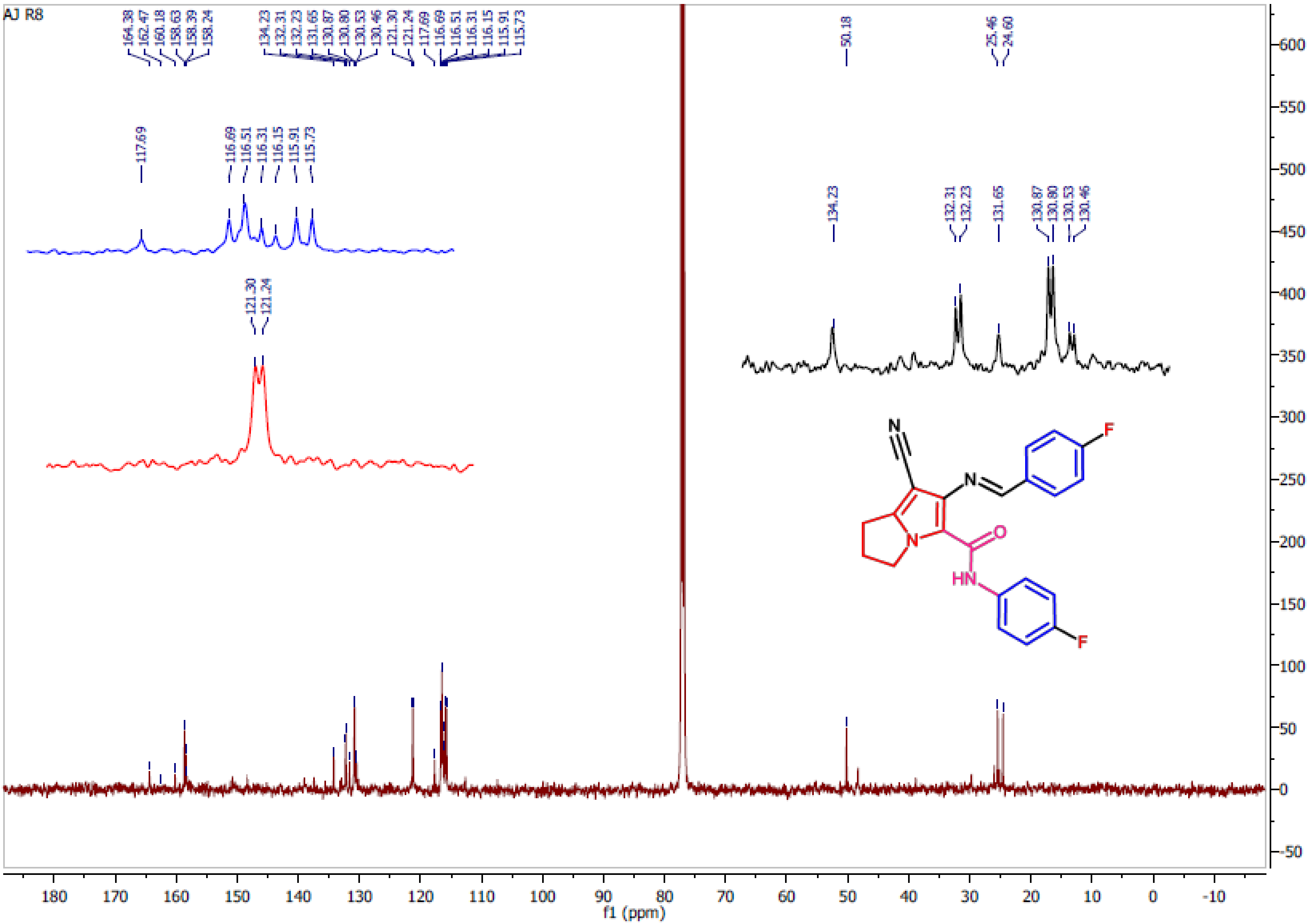

Figure S30. **DEPT C135 ($CDCl_3$, 125 MHz, δ ppm) spectrum of compound 16f**

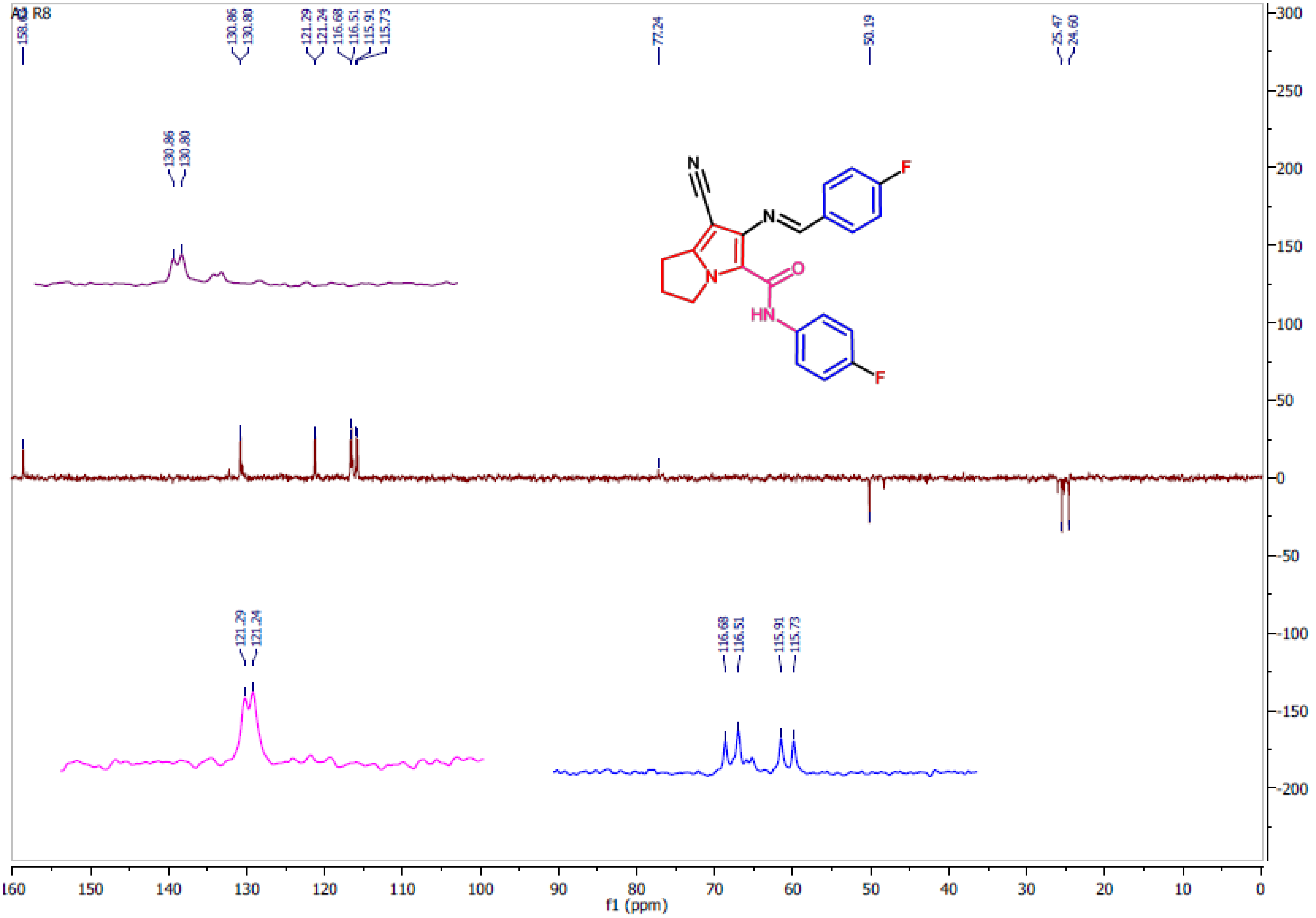

Figure S31. ^{1}H-NMR ($CDCl_3$, 500 MHz, δ ppm) spectrum of compound **16g**

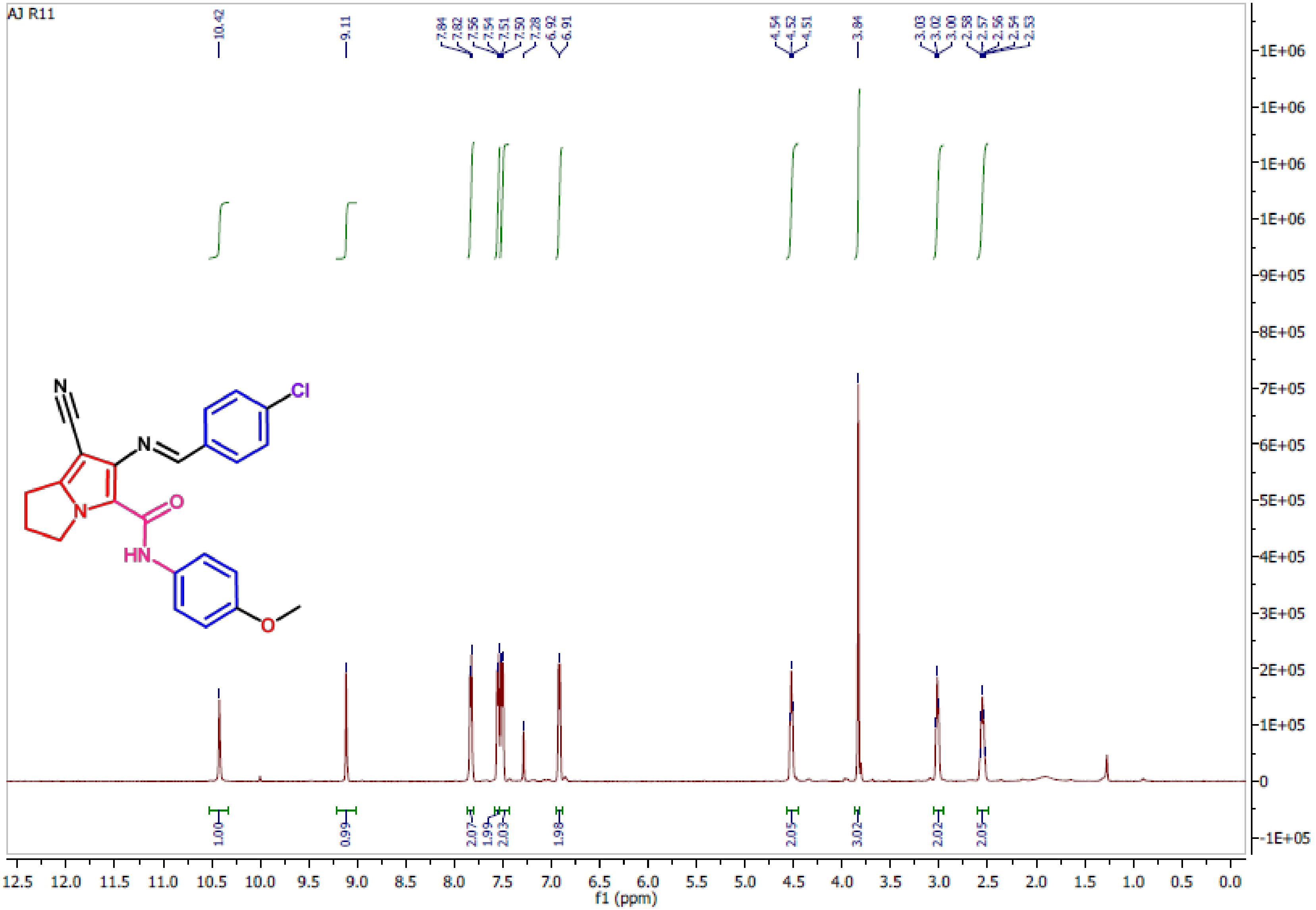

Figure S32. ^{13}C-NMR ($CDCl_3$, 125 MHz, δ ppm) spectrum of compound **16g**

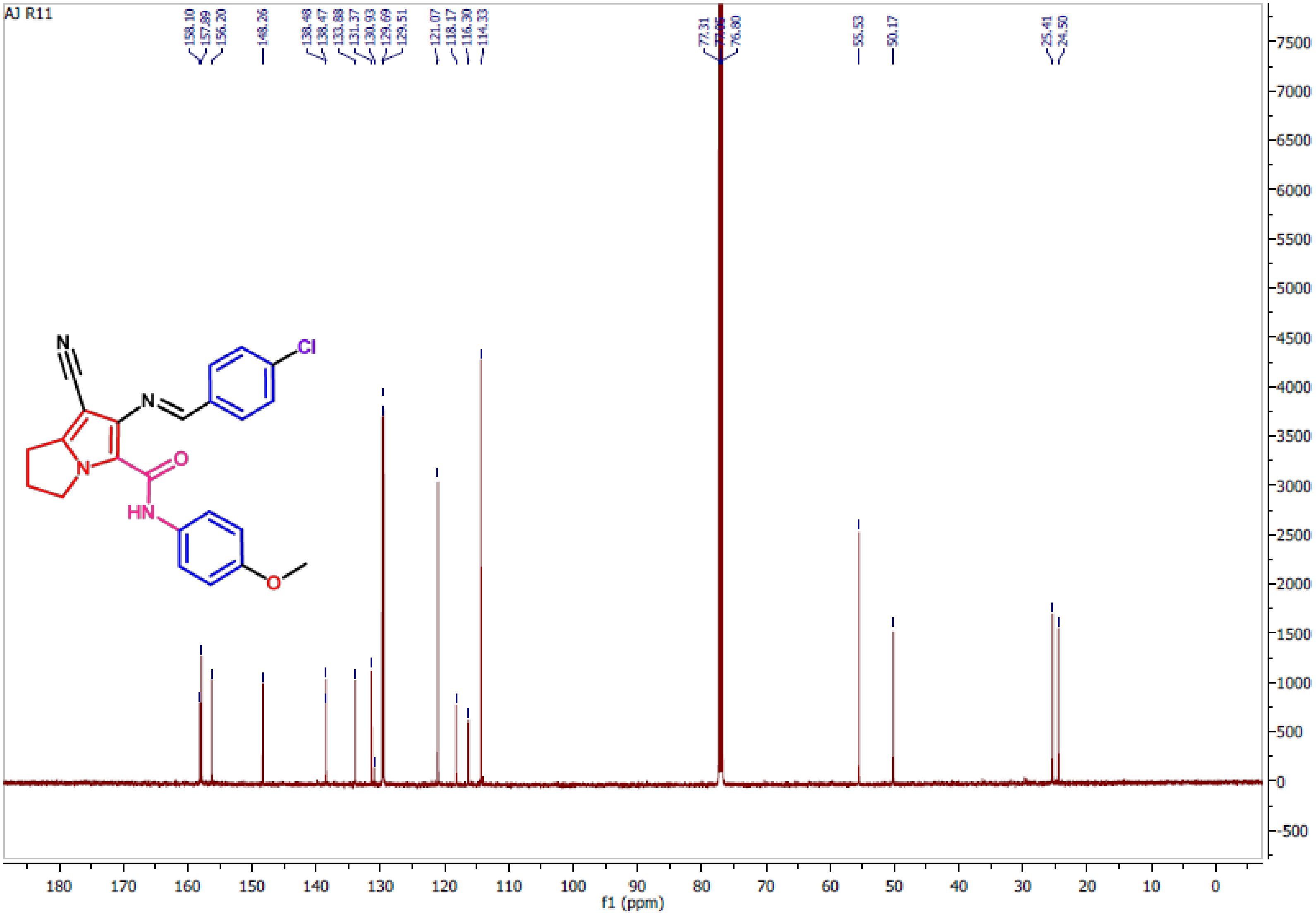

Figure S33. **DEPT C[135] ($CDCl_3$, 125 MHz, δ ppm) spectrum of compound 16g**

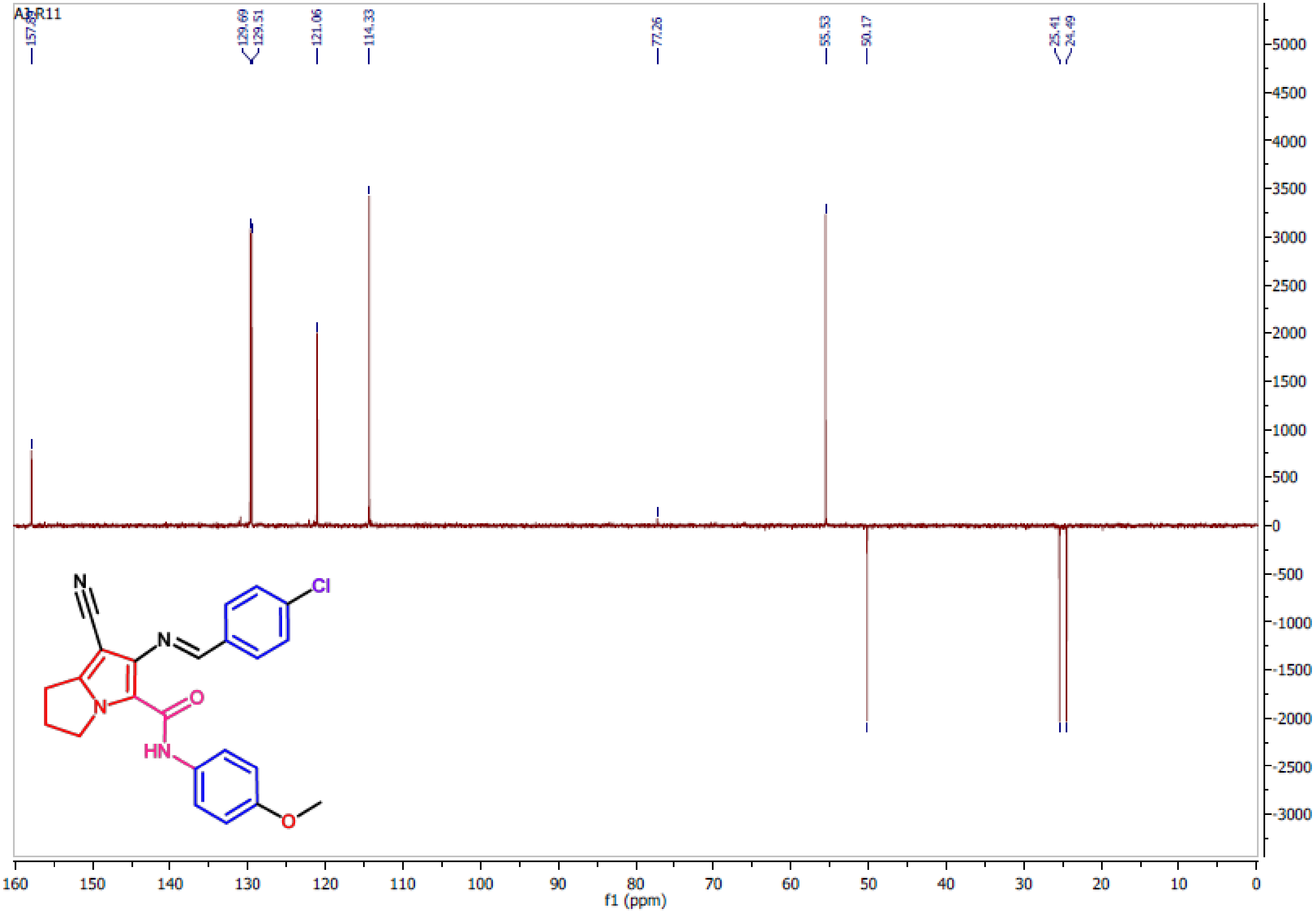

Figure S34. **DEPT C^{135} ($CDCl_3$, 125 MHz, δ ppm) spectrum of compound 16g**

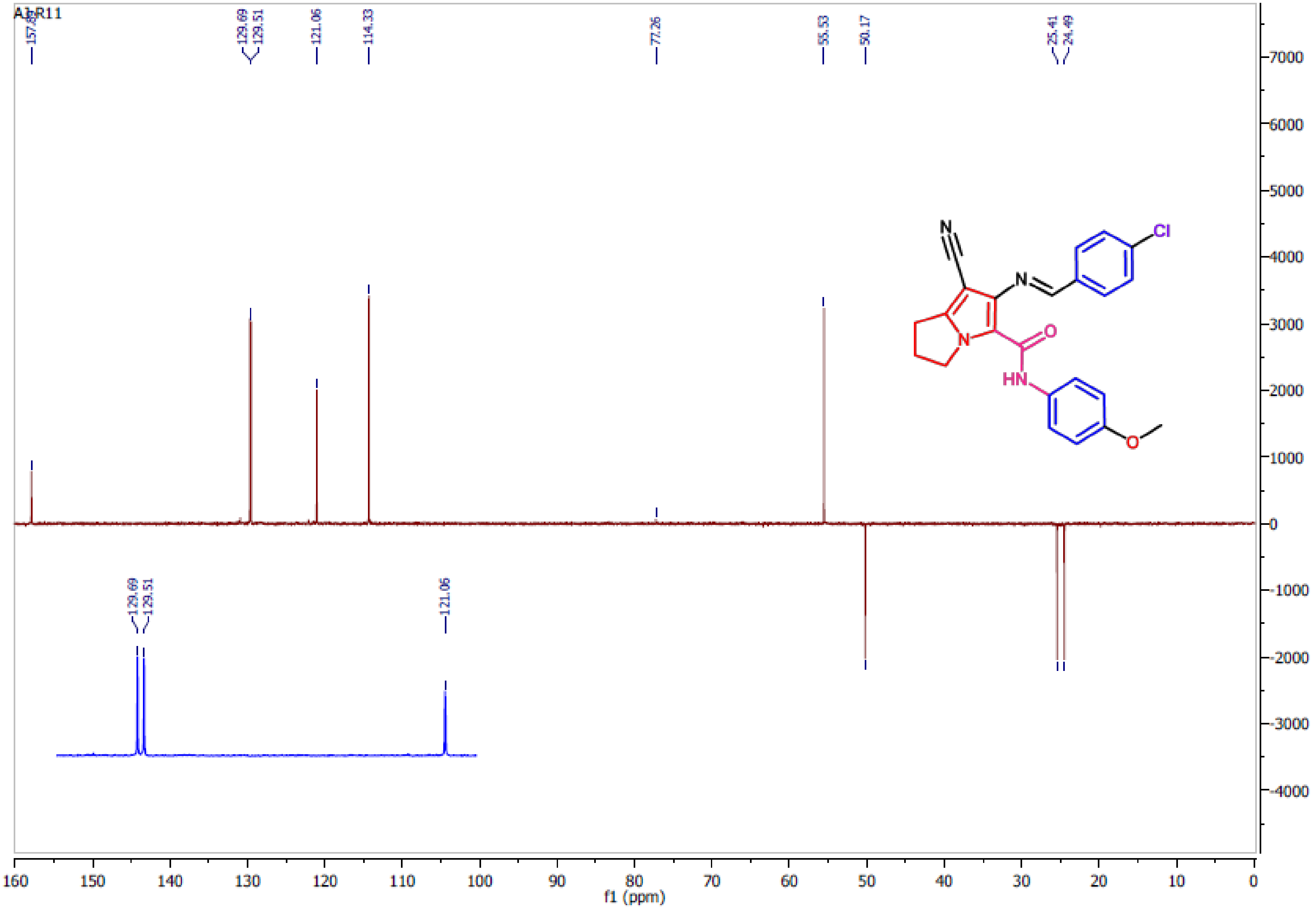

Figure S35. ^{1}H-NMR ($CDCl_3$, 500 MHz, δ ppm) spectrum of compound **16h**

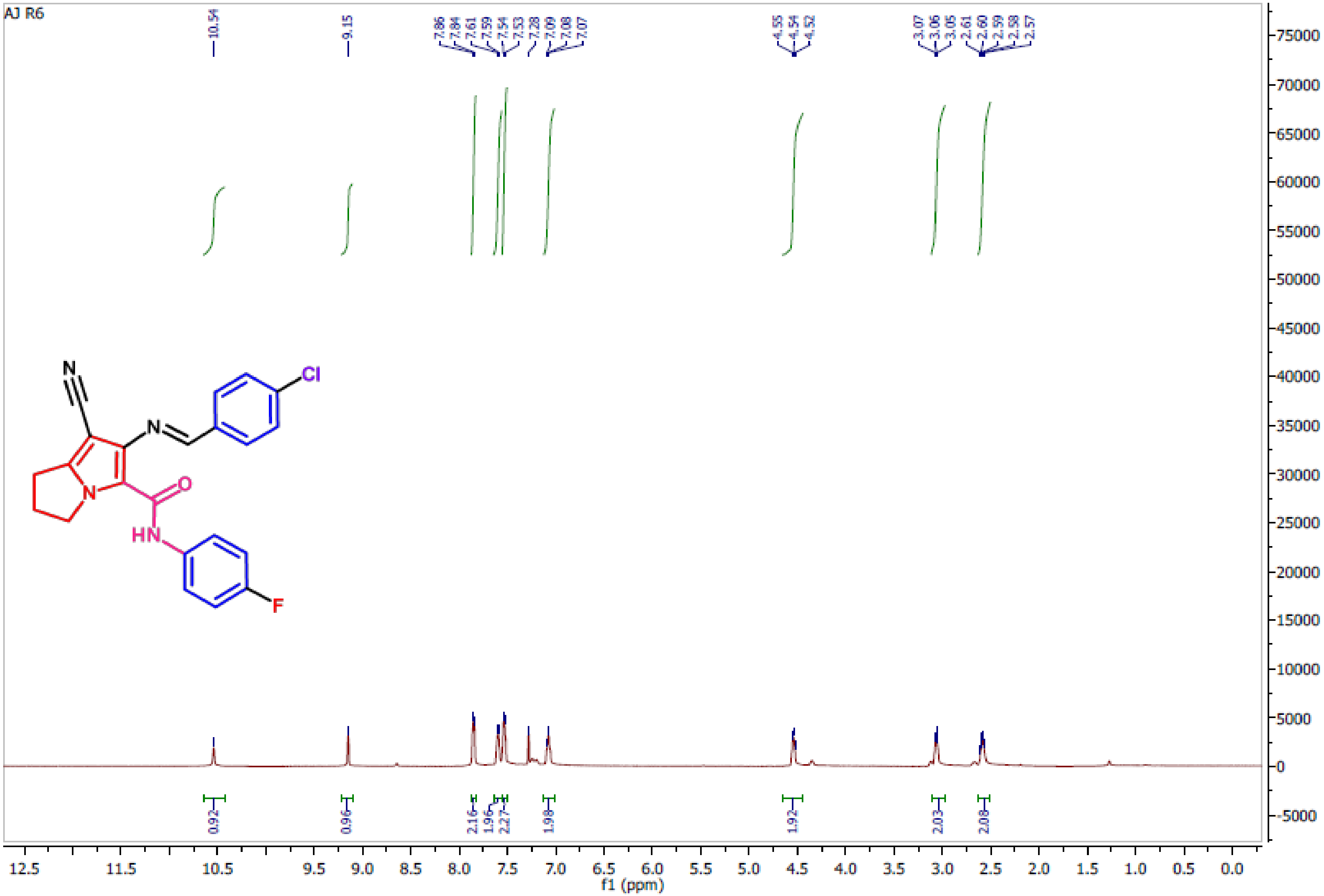

Figure S36. ^{13}C-NMR ($CDCl_3$, 500 MHz, δ ppm) spectrum of compound **16h**

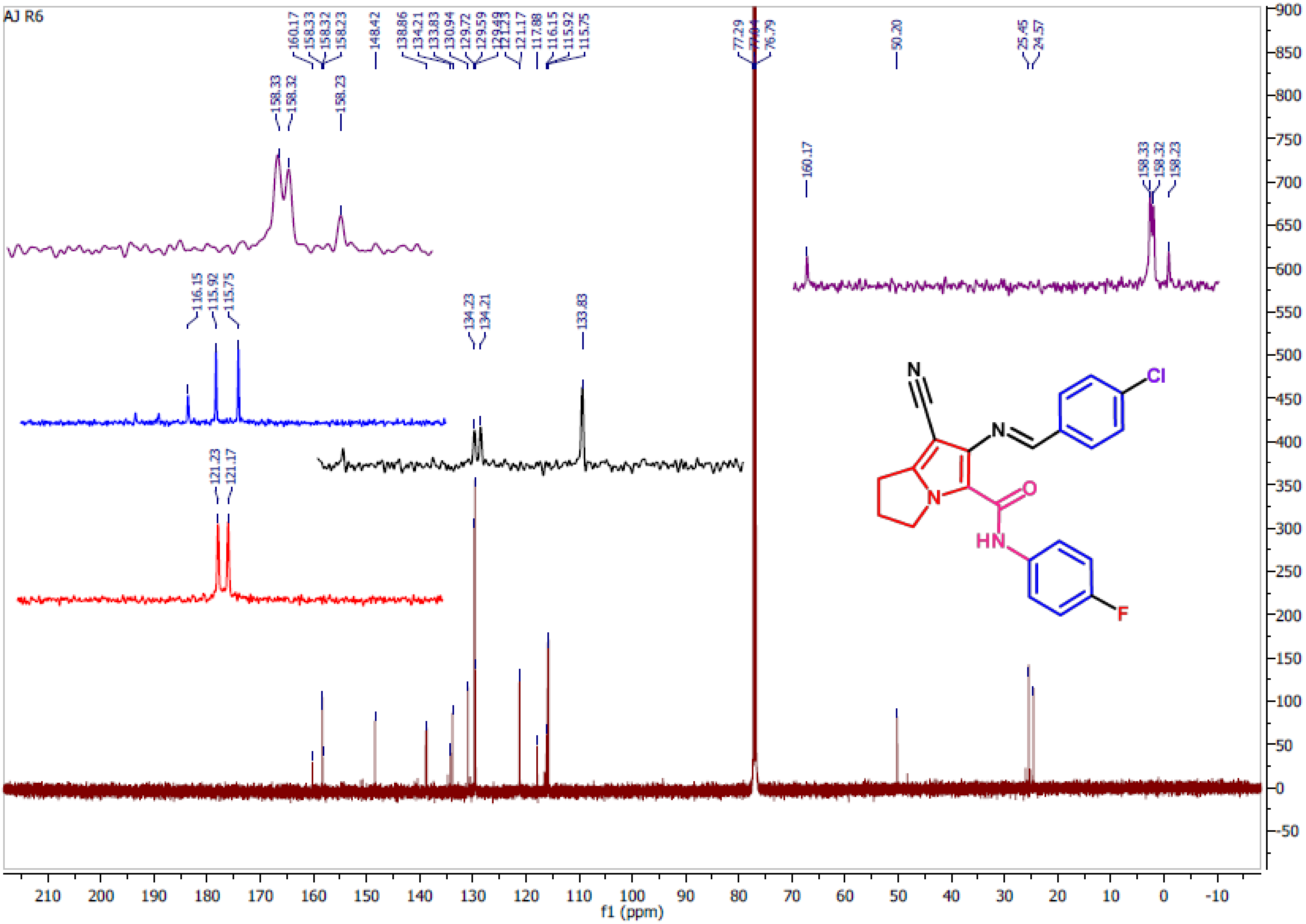

Figure S37. DEPT C^{135} ($CDCl_3$, 125 MHz, δ ppm) spectrum of compound **16h**

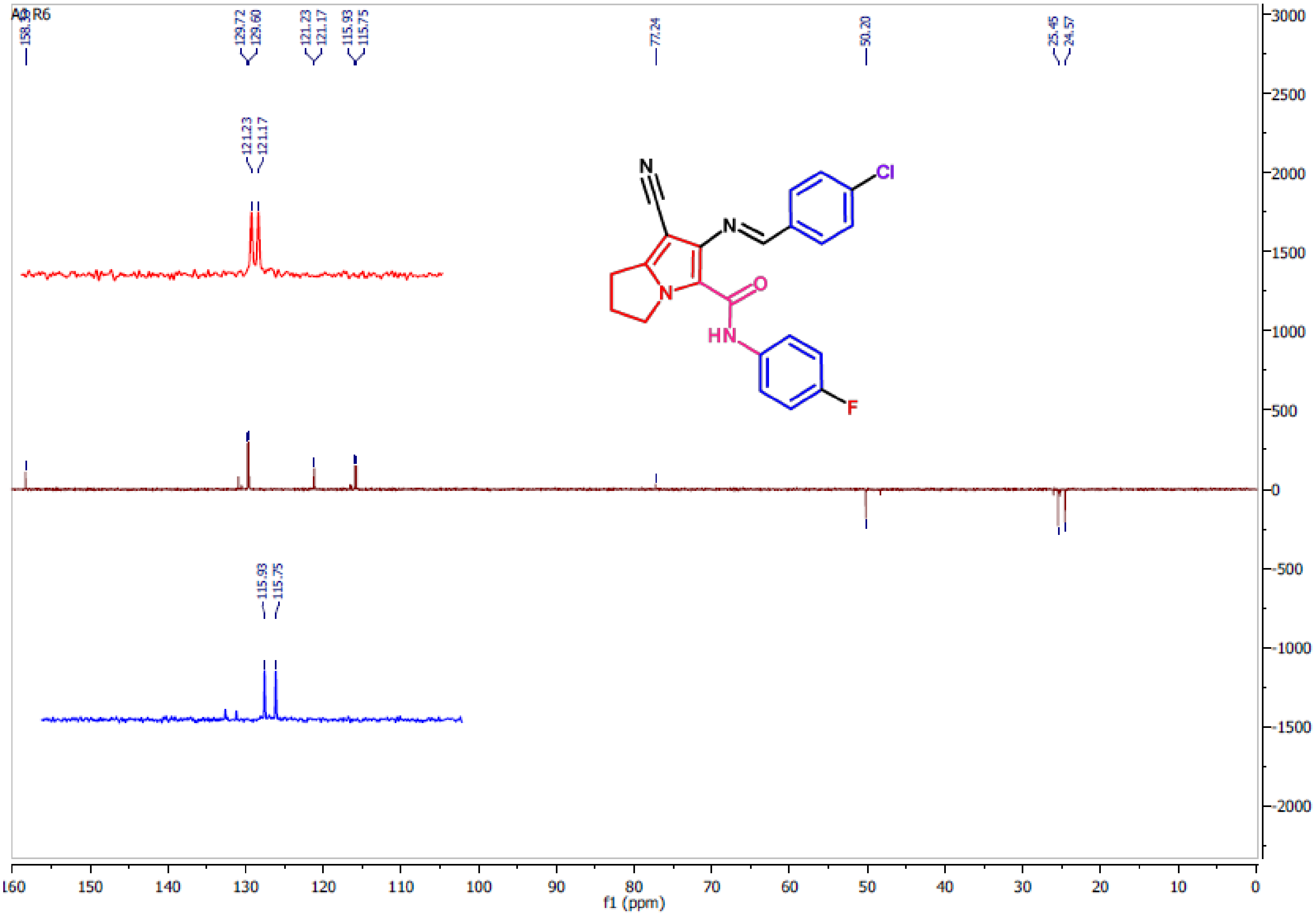

Mass Spectra

Mass spectra were recorded on Shimadzu GCMS QP5050A spectrometer, at 70 eV (EI) at the regional center for mycology and biotechnology, Al-Azhar University.

Figure S38. Mass spectrum of compound 16a

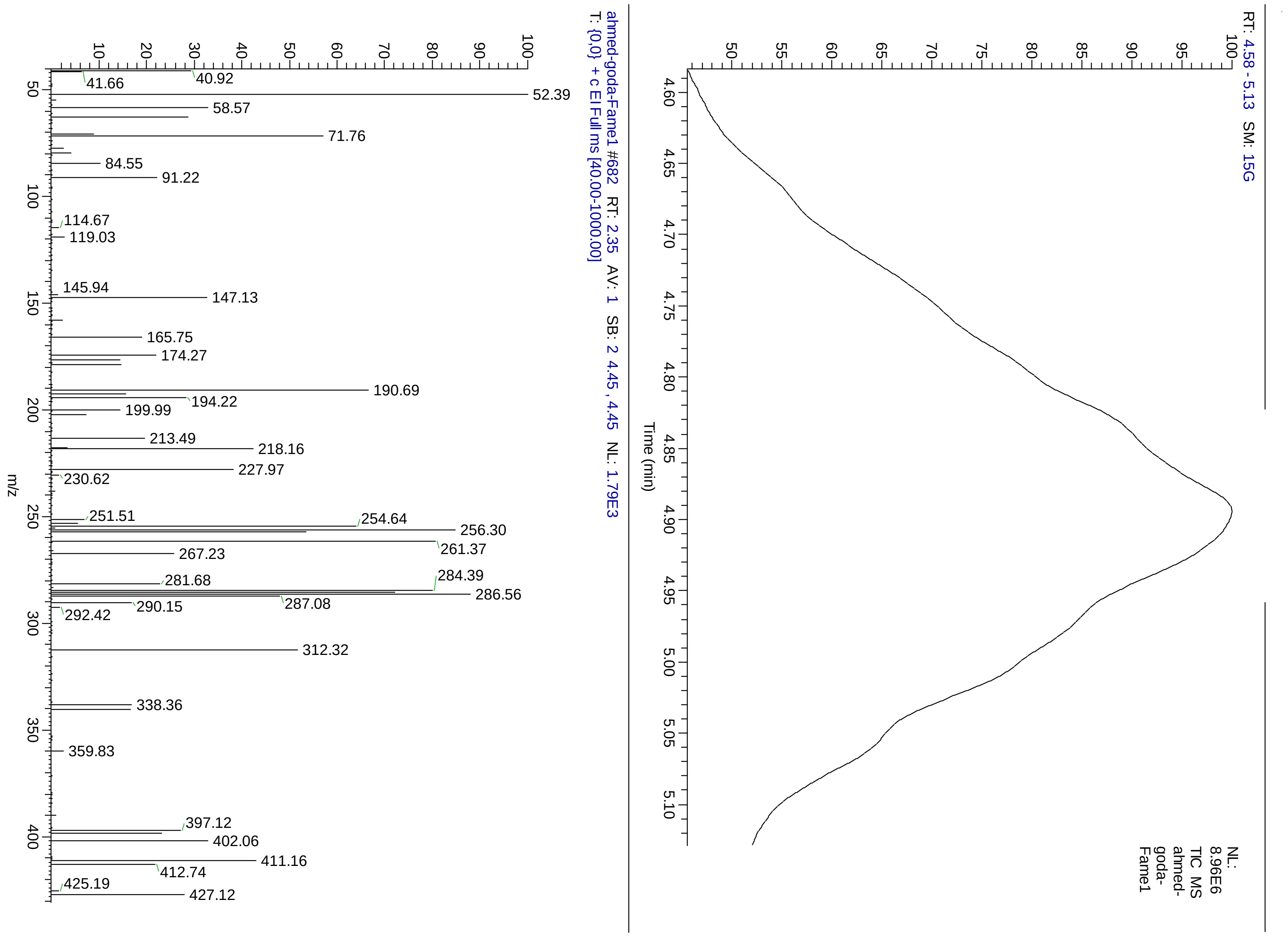

Figure S39. Mass spectrum of compound **16b**

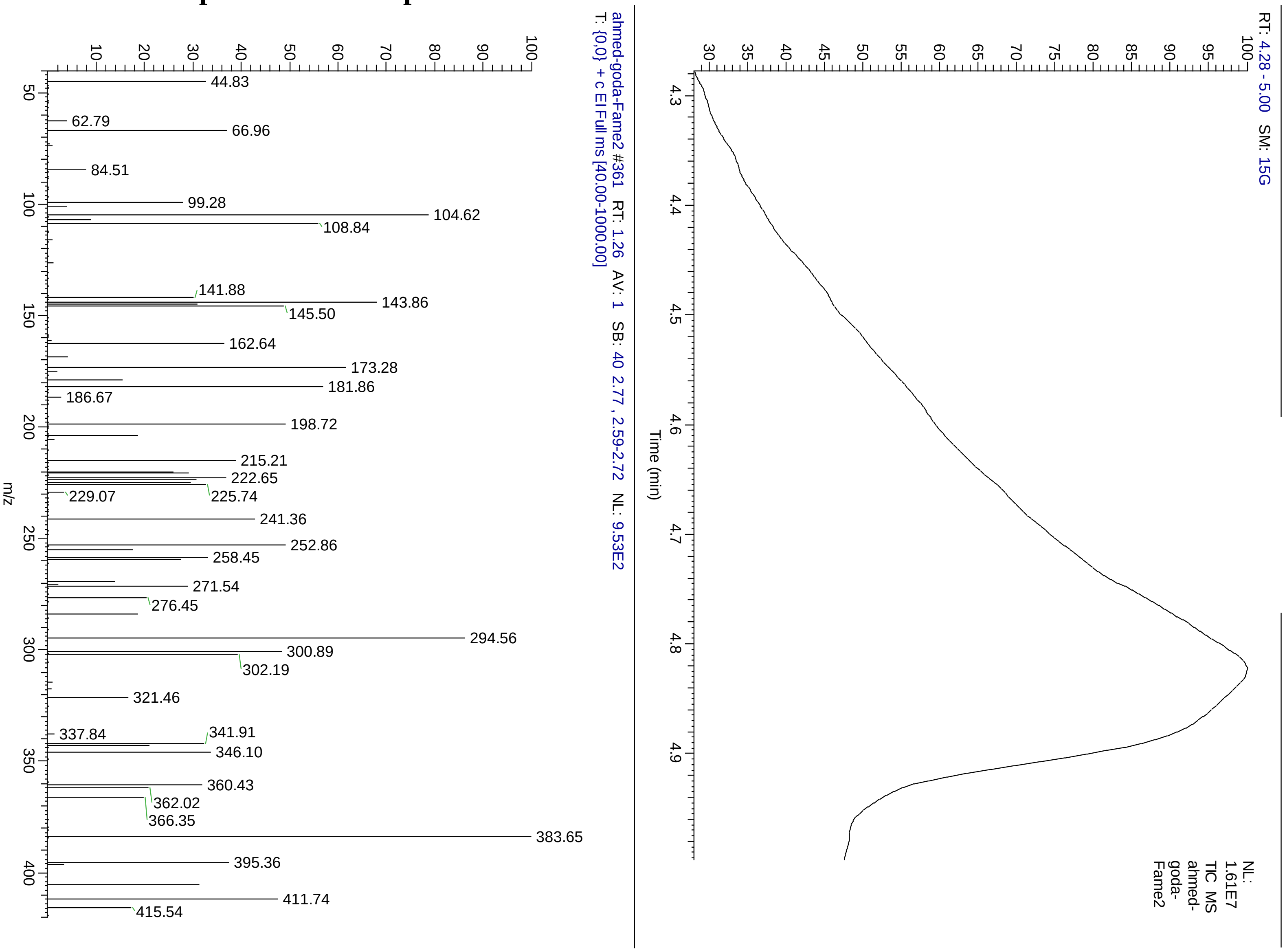

Figure S40. **Mass spectrum of compound 16c**

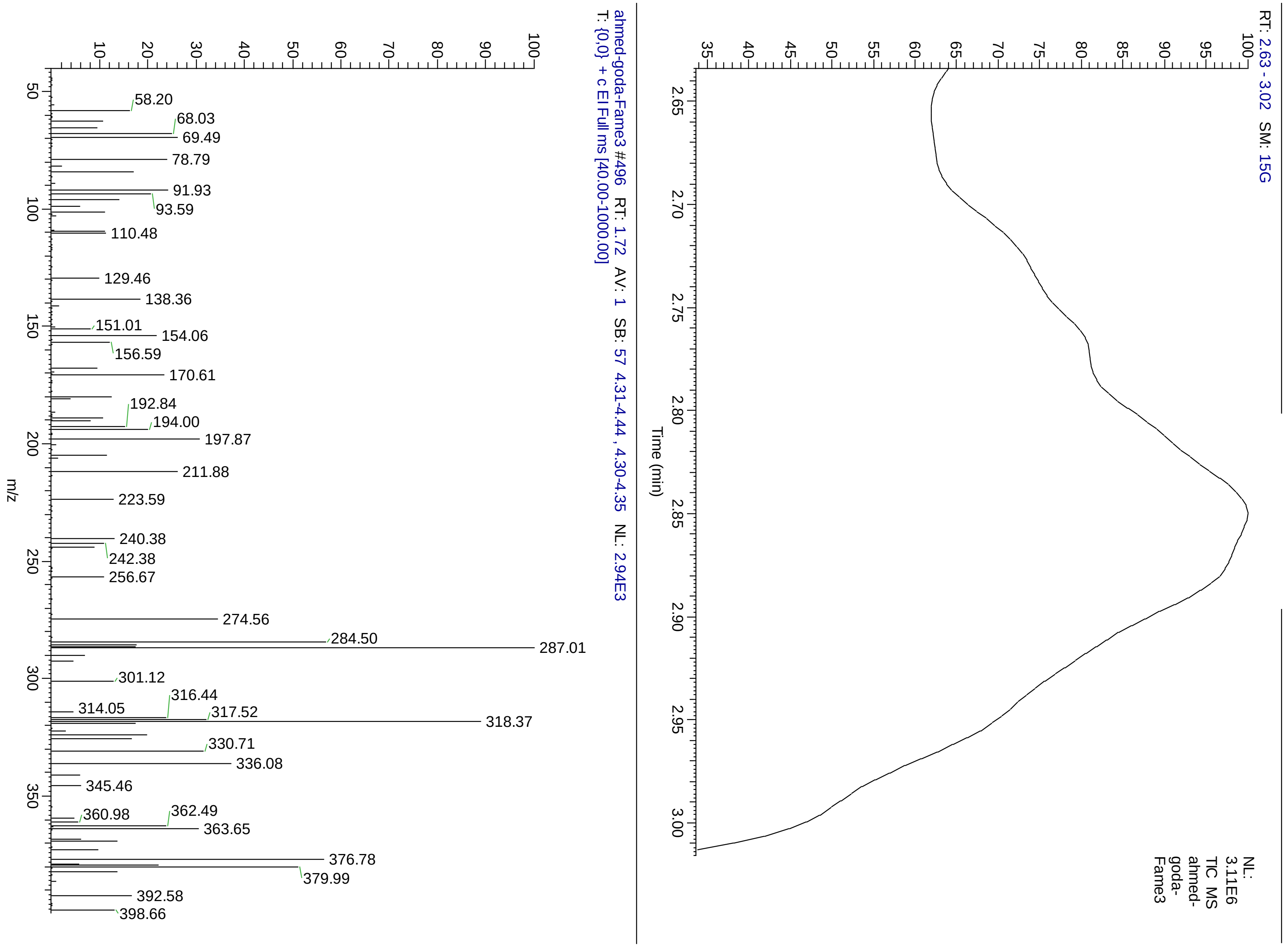

Figure S41. **Mass spectrum of compound 16d**

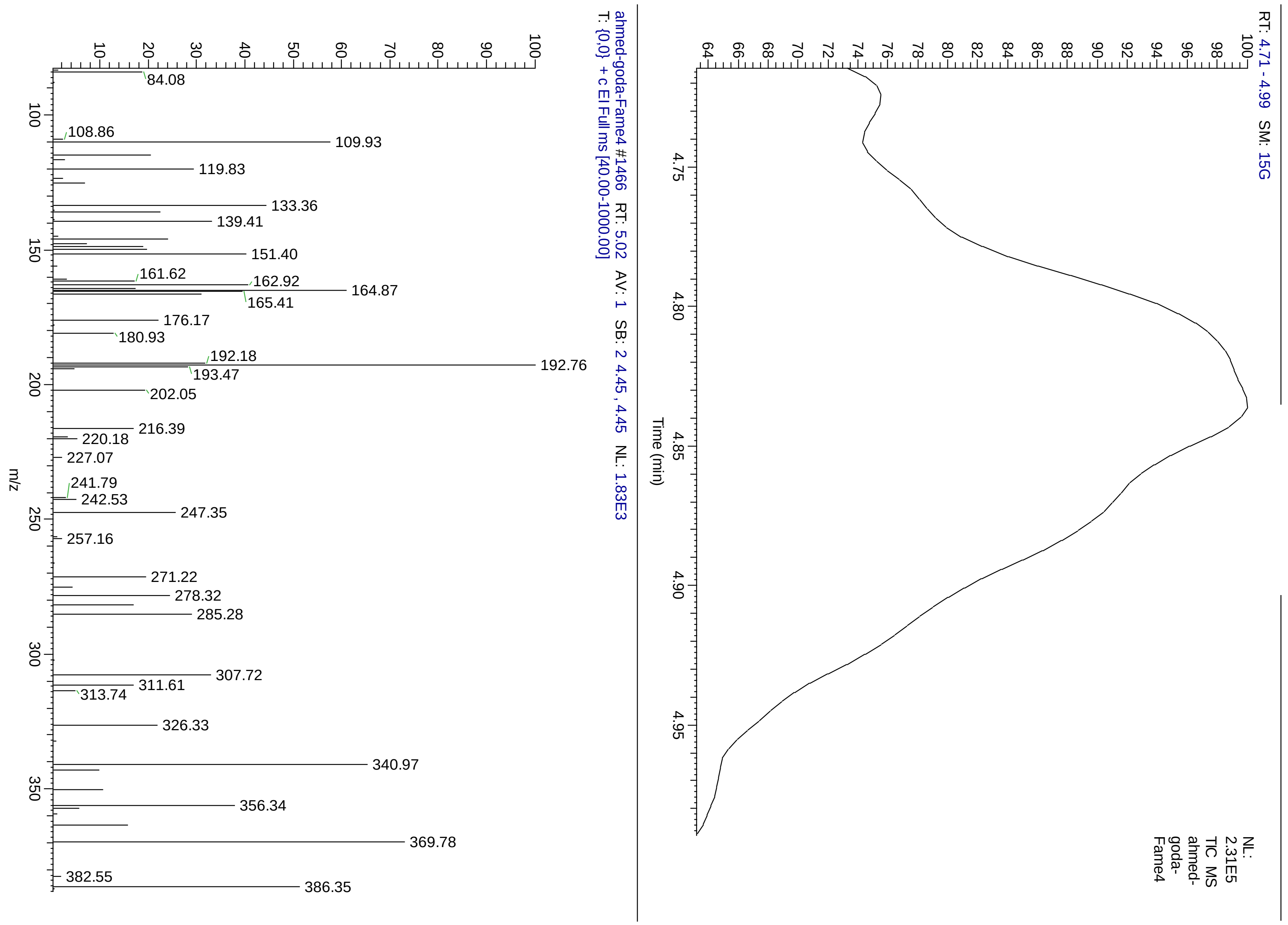

Figure S42. **Mass spectrum of compound 16e**

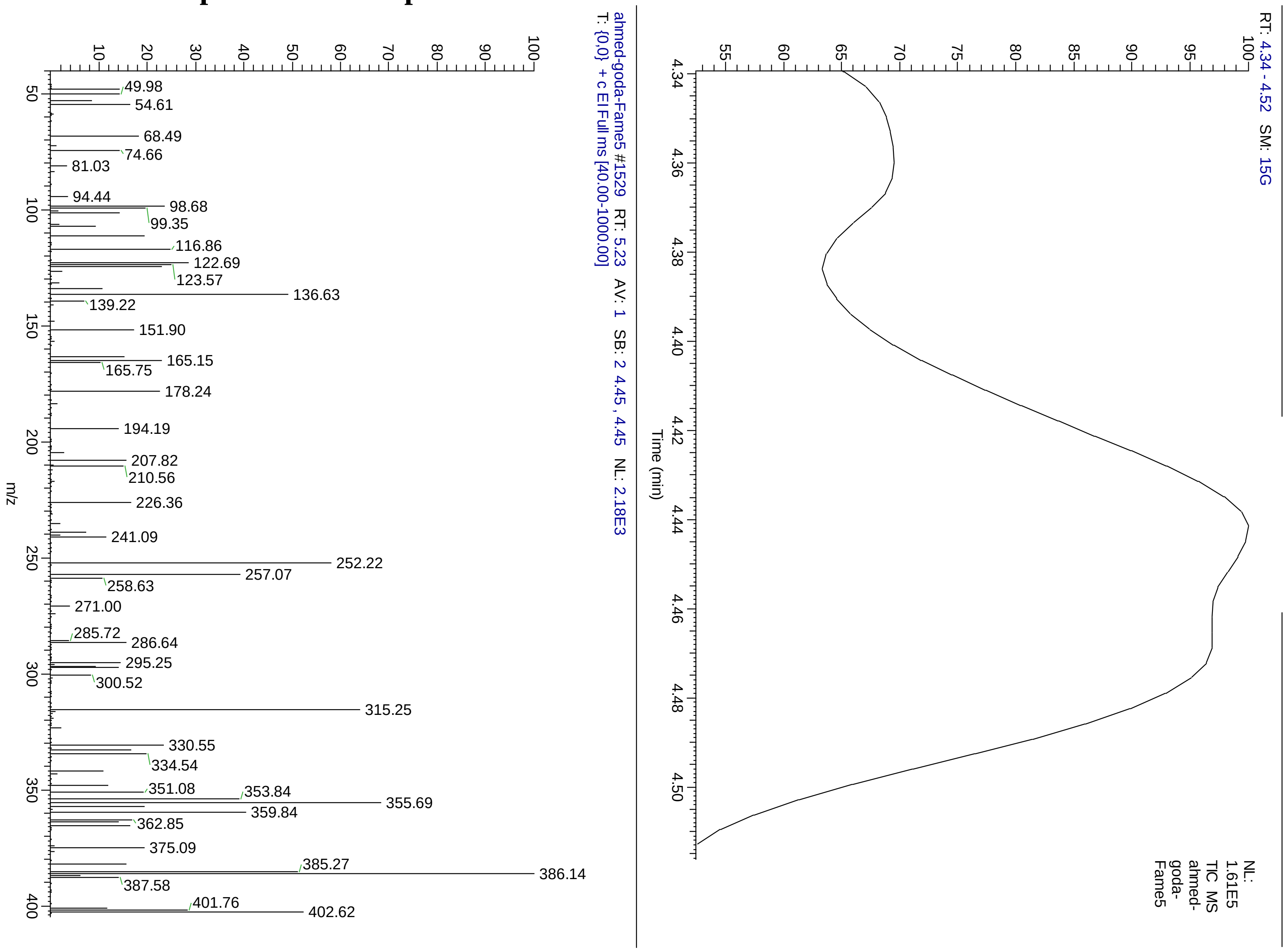

Figure S43. **Mass spectrum of compound 16f**

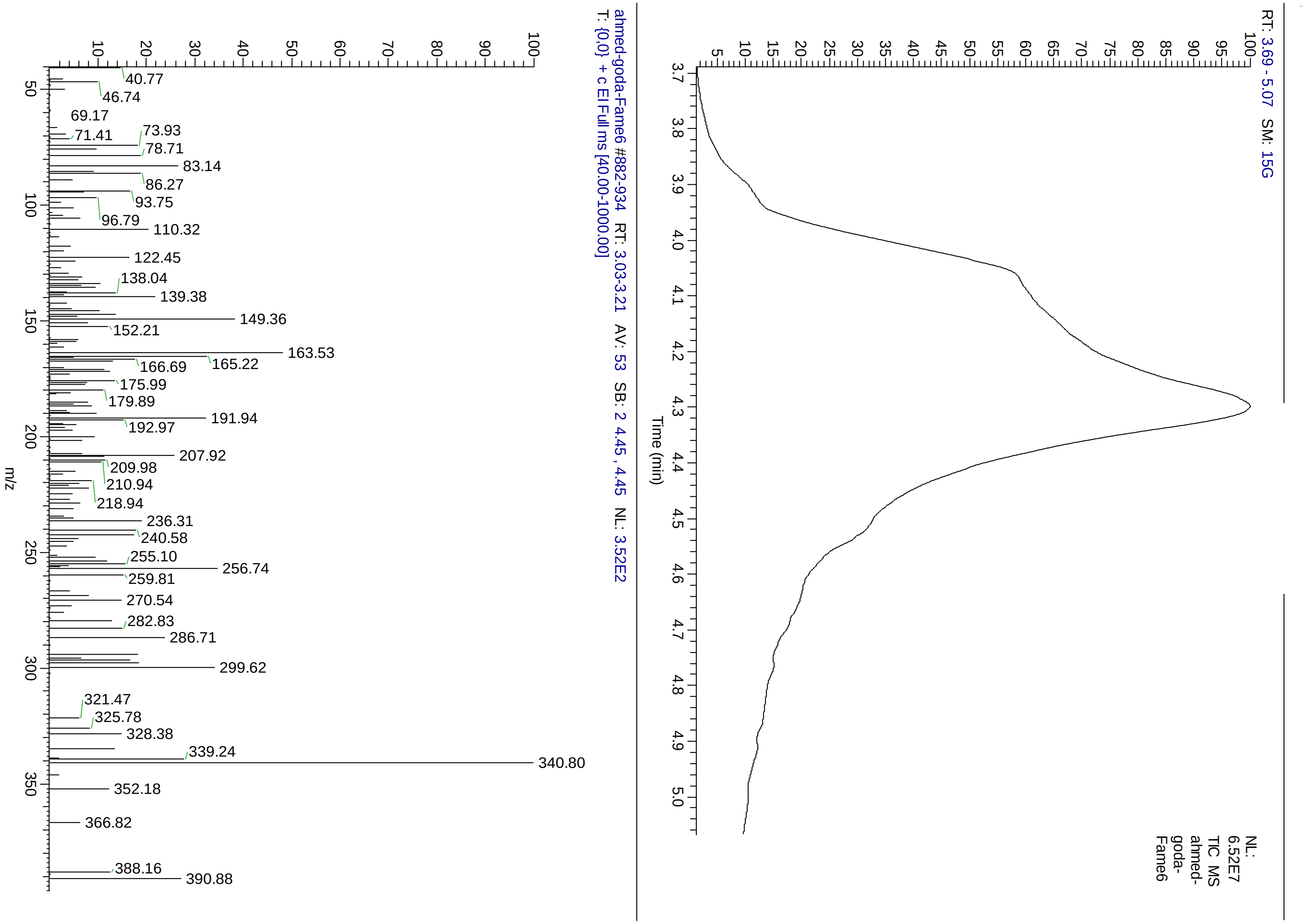

Figure S44. **Mass spectrum of compound 16g**

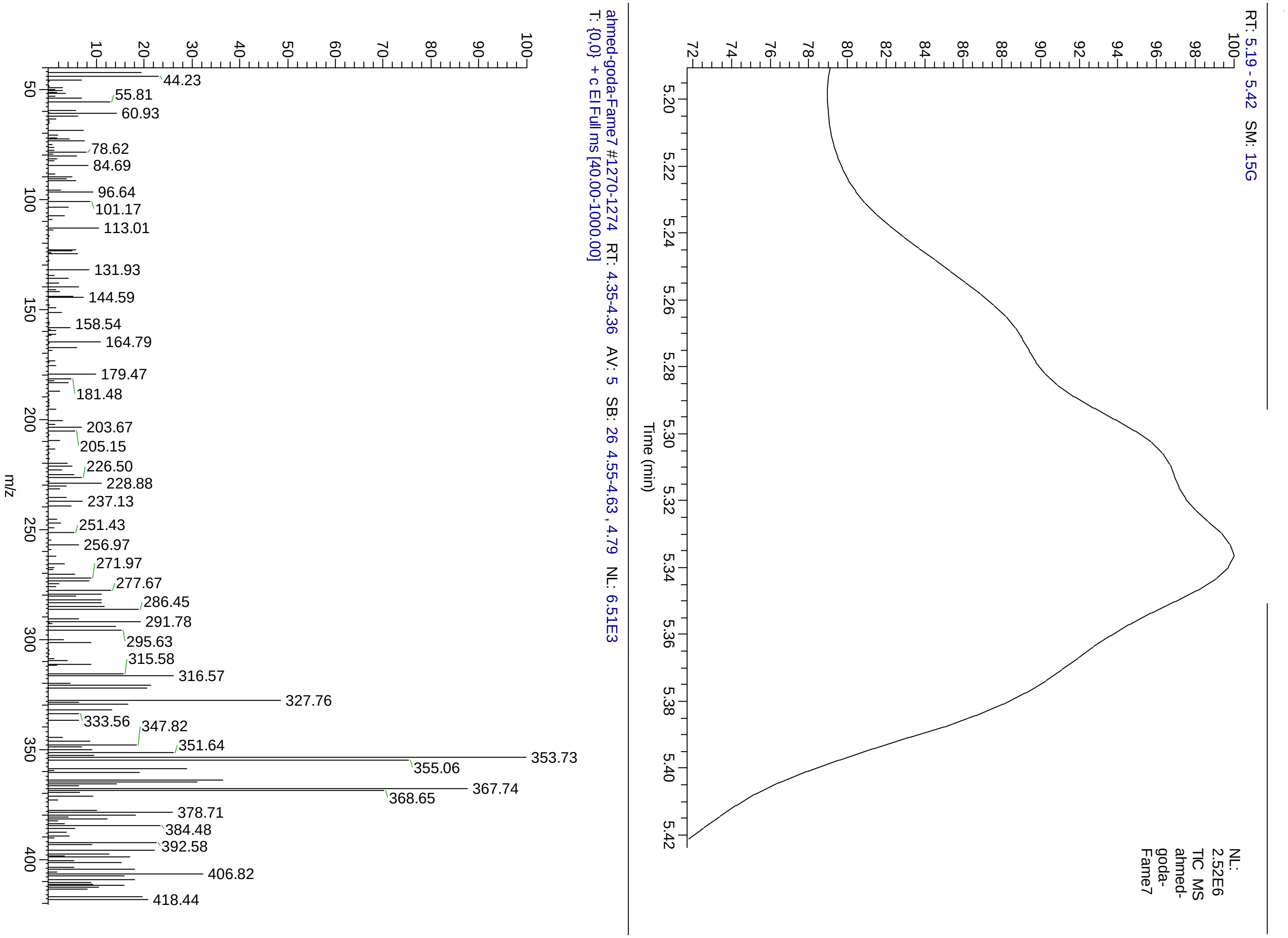

Figure S45. **Mass spectrum of compound 16h**

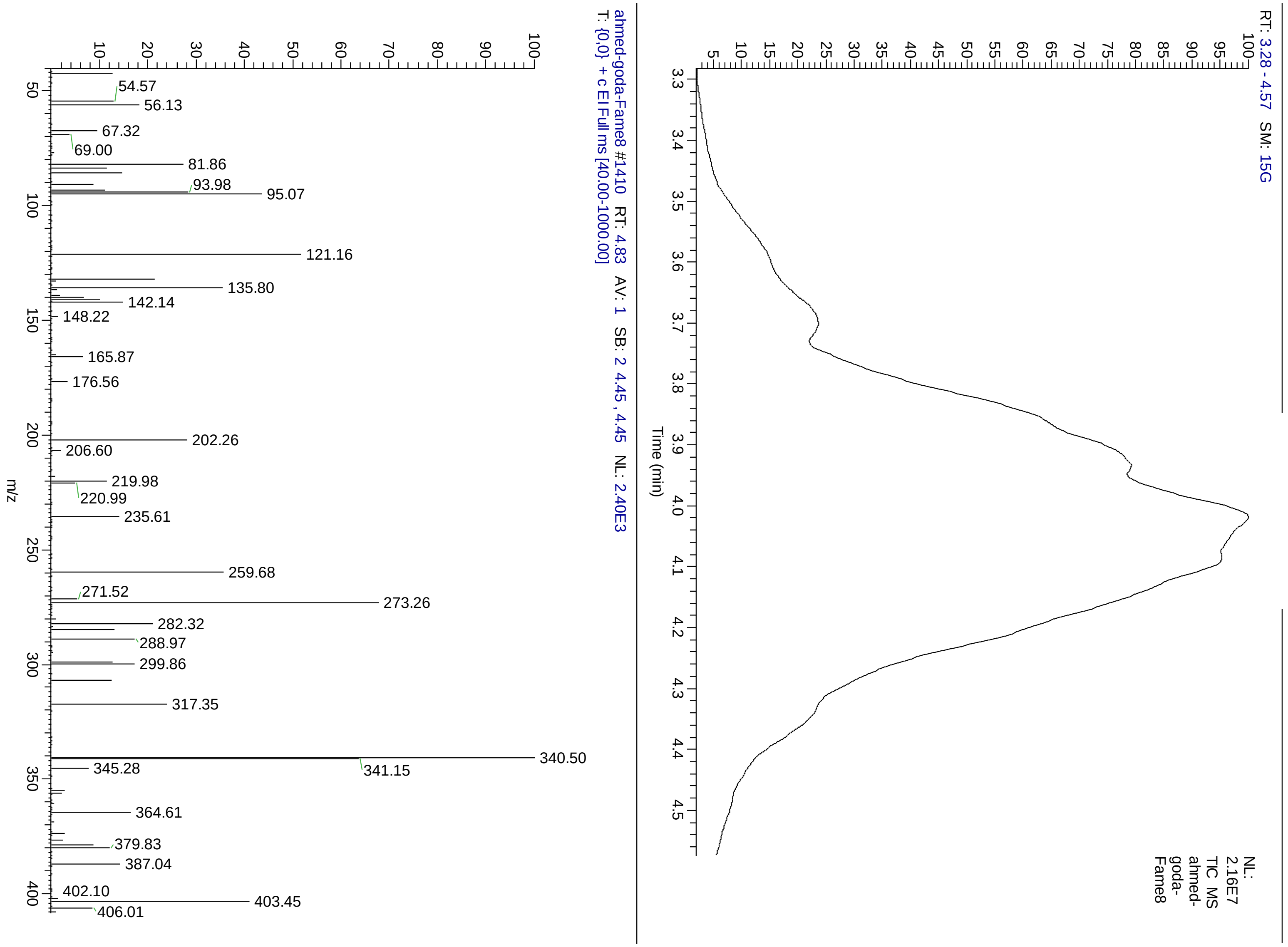

Table 1. Results of target prediction of compound **16h** using SwissTargetPrediction.

Target	Common name	Uniprot ID	ChEMBL ID	Target Class	Probability*	Known actives (3D/2D)
Glycine transporter 1	SLC6A9	P48067	CHEMBL2337	Electrochemical transporter	0.106165761464	173 / 0
Adenosine A3 receptor	ADORA3	P0DMS8	CHEMBL256	Family A G protein-coupled receptor	0.106165761464	799 / 0
Nitric-oxide synthase, brain	NOS1	P29475	CHEMBL3568	Enzyme	0.106165761464	85 / 0
Nitric oxide synthase, inducible	NOS2	P35228	CHEMBL4481	Enzyme	0.106165761464	166 / 0
Adenosine A1 receptor	ADORA1	P30542	CHEMBL226	Family A G protein-coupled receptor	0.106165761464	1274 / 0
Adenosine A2a receptor	ADORA2A	P29274	CHEMBL251	Family A G protein-coupled receptor	0.106165761464	1028 / 0
Cannabinoid receptor 1	CNR1	P21554	CHEMBL218	Family A G protein-coupled receptor	0.106165761464	1677 / 0
Cannabinoid receptor 2	CNR2	P34972	CHEMBL253	Family A G protein-coupled receptor	0.106165761464	1449 / 0
Nicotinamide phosphoribosyltransferase	NAMPT	P43490	CHEMBL1744525	Enzyme	0.106165761464	192 / 0
Carnitine O-palmitoyltransferase 1, liver isoform	CPT1A	P50416	CHEMBL1293194	Enzyme	0.106165761464	191 / 0
Translocator protein (by homology)	TSPO	P30536	CHEMBL5742	Membrane receptor	0.106165761464	541 / 0
Polyadenylate-binding protein 1	PABPC1	P11940	CHEMBL1293286	Unclassified protein	0.106165761464	23 / 0
Monoamine oxidase A	MAOA	P21397	CHEMBL1951	Oxidoreductase	0.106165761464	282 / 0
Serotonin 6 (5-HT6) receptor	HTR6	P50406	CHEMBL3371	Family A G protein-coupled receptor	0.106165761464	320 / 0
Complement factor D	CFD	P00746	CHEMBL2176771	Protease	0.106165761464	296 / 0
Bcl-2-related protein A1	BCL2A1	Q16548	CHEMBL6044	Unclassified protein	0.106165761464	18 / 0
Mineralocorticoid receptor	NR3C2	P08235	CHEMBL1994	Nuclear receptor	0.106165761464	161 / 0
Glucocorticoid receptor	NR3C1	P04150	CHEMBL2034	Nuclear receptor	0.106165761464	426 / 0
Cyclooxygenase-2	PTGS2	P35354	CHEMBL230	Oxidoreductase	0.106165761464	729 / 0
Orexin receptor 2	HCRTR2	O43614	CHEMBL4792	Family A G protein-coupled receptor	0.106165761464	1190 / 0
Orexin receptor 1	HCRTR1	O43613	CHEMBL5113	Family A G protein-coupled receptor	0.106165761464	1016 / 0
G-protein coupled bile acid receptor 1	GPBAR1	Q8TDU6	CHEMBL5409	Family A G protein-coupled receptor	0.106165761464	74 / 0
Leukocyte elastase	ELANE	P08246	CHEMBL248	Protease	0.106165761464	252 / 0
Matrix metalloproteinase 9	MMP9	P14780	CHEMBL321	Protease	0.106165761464	526 / 0
Phosphodiesterase 5A	PDE5A	O76074	CHEMBL1827	Phosphodiesterase	0.106165761464	564 / 0
ATP-binding cassette sub-family G member 2	ABCG2	Q9UNQ0	CHEMBL5393	Primary active transporter	0.106165761464	139 / 0
Elongation of very long chain fatty acids protein 6	ELOVL6	Q9H5J4	CHEMBL5704	Enzyme	0.106165761464	29 / 0
Epidermal growth factor	EGFR	P00533	CHEMBL203	Kinase	0.106165761464	1127 /

Table 1. (continued)

Target	Common name	Uniprot ID	ChEMBL ID	Target Class	Probability*	Known actives (3D/2D)
receptor erbB1						0
Matrix metalloproteinase 13	MMP13	P45452	CHEMBL280	Protease	0.106165761464	418 / 0
Tyrosine-protein kinase ITK/TSK	ITK	Q08881	CHEMBL2959	Kinase	0.106165761464	262 / 0
Sodium channel protein type X alpha subunit (by homology)	SCN10A	Q9Y5Y9	CHEMBL5451	Voltage-gated ion channel	0.106165761464	68 / 0
Isocitrate dehydrogenase [NADP] cytoplasmic	IDH1	O75874	CHEMBL2007625	Enzyme	0.106165761464	247 / 0
Gamma-secretase	PSEN2<br>PSENEN<br>NCSTN<br>APH1A<br>PSEN1<br>APH1B	P49810<br>Q9NZ42<br>Q92542<br>Q96BI3<br>P49768<br>Q8WW43	CHEMBL2094135	Protease	0.106165761464	461 / 0
Epoxide hydratase	EPHX2	P34913	CHEMBL2409	Protease	0.106165761464	443 / 0
MAP kinase p38 alpha	MAPK14	Q16539	CHEMBL260	Kinase	0.106165761464	1543 / 0
Bradykinin B1 receptor	BDKRB1	P46663	CHEMBL4308	Family A G protein-coupled receptor	0.106165761464	61 / 0
Tankyrase-2	TNKS2	Q9H2K2	CHEMBL6154	Enzyme	0.106165761464	81 / 0
Sodium/calcium exchanger 1	SLC8A1	P32418	CHEMBL4076	Electrochemical transporter	0.106165761464	111 / 0
NAD-dependent deacetylase sirtuin 2	SIRT2	Q8IXJ6	CHEMBL4462	Eraser	0.106165761464	160 / 0
Glucokinase regulatory protein	GCKR	Q14397	CHEMBL1075152	Enzyme	0.106165761464	14 / 0
Monoamine oxidase B	MAOB	P27338	CHEMBL2039	Oxidoreductase	0.106165761464	589 / 0
SUMO-activating enzyme	SAE1<br>UBA2	Q9UBE0<br>Q9UBT2	CHEMBL2095174	Enzyme	0.106165761464	27 / 0
Neurokinin 1 receptor	TACR1	P25103	CHEMBL249	Family A G protein-coupled receptor	0.106165761464	270 / 0
Matrix metalloproteinase 2	MMP2	P08253	CHEMBL333	Protease	0.106165761464	553 / 0
Prostanoid EP3 receptor	PTGER3	P43115	CHEMBL3710	Family A G protein-coupled receptor	0.106165761464	66 / 0
Carnitine O-palmitoyltransferase 1, muscle isoform	CPT1B	Q92523	CHEMBL2216739	Group translocator	0.106165761464	12 / 0
Cathepsin S	CTSS	P25774	CHEMBL2954	Protease	0.106165761464	519 / 0
Cyclin-dependent kinase 2	CDK2	P24941	CHEMBL301	Kinase	0.106165761464	424 / 0
Smoothened homolog	SMO	Q99835	CHEMBL5971	Frizzled family G protein-coupled receptor	0.106165761464	250 / 0
Dihydroorotate dehydrogenase	DHODH	Q02127	CHEMBL1966	Oxidoreductase	0.106165761464	34 / 0
Dopamine D2 receptor	DRD2	P14416	CHEMBL217	Family A G protein-coupled receptor	0.106165761464	666 / 0
Dopamine D4 receptor	DRD4	P21917	CHEMBL219	Family A G protein-coupled receptor	0.106165761464	320 / 0
Tyrosine-protein kinase	JAK2	O60674	CHEMBL2971	Kinase	0.106165761464	694 / 0

Table 1. (continued)

Target	Common name	Uniprot ID	ChEMBL ID	Target Class	Probability*	Known actives (3D/2D)
JAK2						
Tyrosine-protein kinase TYK2	TYK2	P29597	CHEMBL3553	Kinase	0.106165761464	135 / 0
Hexokinase type IV	GCK	P35557	CHEMBL3820	Enzyme	0.106165761464	231 / 0
MAP kinase p38 beta	MAPK11	Q15759	CHEMBL3961	Kinase	0.106165761464	98 / 0
MAP kinase ERK2	MAPK1	P28482	CHEMBL4040	Kinase	0.106165761464	542 / 0
Dual specificity phosphatase Cdc25B	CDC25B	P30305	CHEMBL4804	Phosphatase	0.106165761464	40 / 0
Proto-oncogene protein Wnt-3	WNT3	P56703	CHEMBL6079	Unclassified protein	0.106165761464	16 / 0
EZH2/SUZ12/EED/ RBBP7/RBBP4	EZH2	Q15910	CHEMBL2189110	Writer	0.106165761464	31 / 0
Calpain 1	CAPN1	P07384	CHEMBL3891	Protease	0.106165761464	262 / 0
Fibroblast activation protein alpha (by homology)	FAP	Q12884	CHEMBL4683	Protease	0.106165761464	78 / 0
Proto-oncogene tyrosine-protein kinase MER	MERTK	Q12866	CHEMBL5331	Kinase	0.106165761464	17 / 0
Vascular endothelial growth factor receptor 1	FLT1	P17948	CHEMBL1868	Kinase	0.106165761464	213 / 0
Potassium channel subfamily K member 3	KCNK3	O14649	CHEMBL2321613	Voltage-gated ion channel	0.106165761464	45 / 0
Potassium channel subfamily K member 9	KCNK9	Q9NPC2	CHEMBL2321614	Voltage-gated ion channel	0.106165761464	32 / 0
Phosphoglycerate kinase 1	PGK1	P00558	CHEMBL2886	Enzyme	0.106165761464	38 / 0
Cathepsin L	CTSL	P07711	CHEMBL3837	Protease	0.106165761464	412 / 0
dUTP pyrophosphatase	DUT	P33316	CHEMBL5203	Enzyme	0.106165761464	43 / 0
G-protein coupled receptor 55	GPR55	Q9Y2T6	CHEMBL1075322	Family A G protein-coupled receptor	0.106165761464	40 / 0
Microtubule-associated protein tau	MAPT	P10636	CHEMBL1293224	Unclassified protein	0.106165761464	12 / 0
Nuclear receptor ROR-gamma	RORC	P51449	CHEMBL1741186	Nuclear receptor	0.106165761464	238 / 0
Corticotropin releasing factor receptor 1 (by homology)	CRHR1	P34998	CHEMBL1800	Family B G protein-coupled receptor	0.106165761464	590 / 0
Sodium channel protein type V alpha subunit	SCN5A	Q14524	CHEMBL1980	Voltage-gated ion channel	0.106165761464	54 / 0
Beta amyloid A4 protein	APP	P05067	CHEMBL2487	Membrane receptor	0.106165761464	64 / 0
P2X purinoceptor 3	P2RX3	P56373	CHEMBL2998	Ligand-gated ion channel	0.106165761464	77 / 0
Protein kinase C beta	PRKCB	P05771	CHEMBL3045	Kinase	0.106165761464	31 / 0
Phosphodiesterase 9A	PDE9A	O76083	CHEMBL3535	Phosphodiesterase	0.106165761464	51 / 0
Voltage-gated potassium channel subunit Kv1.5	KCNA5	P22460	CHEMBL4306	Voltage-gated ion channel	0.106165761464	271 / 0
Alpha-synuclein	SNCA	P37840	CHEMBL6152	Unclassified protein	0.106165761464	8 / 0
Glucagon receptor	GCGR	P47871	CHEMBL1985	Family B G protein-coupled receptor	0.106165761464	68 / 0

Table 1. (continued)

Target	Common name	Uniprot ID	ChEMBL ID	Target Class	Probability*	Known actives (3D/2D)
GABA-A receptor; alpha-3/ beta-3/gamma-2	GABRB3 GABRA3 GABRG2	P28472 P34903 P18507	CHEMBL2094120	Ligand-gated ion channel	0.106165761464	309 / 0
GABA-A receptor; alpha-1/ beta-3/gamma-2	GABRB3 GABRG2 GABRA1	P28472 P18507 P14867	CHEMBL2094121	Ligand-gated ion channel	0.106165761464	252 / 0
GABA-A receptor; alpha-5/ beta-3/gamma-2	GABRB3 GABRG2 GABRA5	P28472 P18507 P31644	CHEMBL2094122	Ligand-gated ion channel	0.106165761464	275 / 0
GABA-A receptor; alpha-2/ beta-3/gamma-2	GABRA2 GABRB3 GABRG2	P47869 P28472 P18507	CHEMBL2094130	Ligand-gated ion channel	0.106165761464	283 / 0
6-phosphofructo-2-kinase/ fructose-2,6-bisphosphatase 3	PFKFB3	Q16875	CHEMBL2331053	Enzyme	0.106165761464	268 / 0
Neuropeptide Y receptor type 5	NPY5R	Q15761	CHEMBL4561	Family A G protein-coupled receptor	0.106165761464	507 / 0
Serine/threonine-protein kinase Chk1	CHEK1	O14757	CHEMBL4630	Kinase	0.106165761464	189 / 0
Histone deacetylase 6	HDAC6	Q9UBN7	CHEMBL1865	Eraser	0.106165761464	209 / 0
Anandamide amidohydrolase	FAAH	O00519	CHEMBL2243	Enzyme	0.106165761464	441 / 0
Hormone sensitive lipase	LIPE	Q05469	CHEMBL3590	Enzyme	0.106165761464	49 / 0
Proteinase-activated receptor 1	F2R	P25116	CHEMBL3974	Family A G protein-coupled receptor	0.106165761464	72 / 0
Vanilloid receptor	TRPV1	Q8NER1	CHEMBL4794	Voltage-gated ion channel	0.106165761464	596 / 0
Tankyrase-1	TNKS	O95271	CHEMBL6164	Enzyme	0.106165761464	91 / 0
Dopamine D3 receptor	DRD3	P35462	CHEMBL234	Family A G protein-coupled receptor	0.106165761464	301 / 0
Cholecystokinin B receptor	CCKBR	P32239	CHEMBL298	Family A G protein-coupled receptor	0.106165761464	551 / 0
Cathepsin (V and K)	CTSV	O60911	CHEMBL3272	Protease	0.106165761464	69 / 0
Serine/threonine-protein kinase B-raf	BRAF	P15056	CHEMBL5145	Kinase	0.106165761464	304 / 0
Acid ceramidase (by homology)	ASAH1	Q13510	CHEMBL5463	Enzyme	0.106165761464	33 / 0
Nucleotide-binding oligomerization domain-containing protein 1	NOD1	Q9Y239	CHEMBL1293222	Unclassified protein	0.106165761464	19 / 0

Table 2. Result of the docking study of compound 16g into COX-2, MAP p38α, EGFR, CDK2, BRAF, VEGFR1 in comparison to the co-crystallized ligad

Target (pdb)	Ligand	ΔG_b [a]	K_i [b]
**COX-2 (3LN1)**	**16g**	-10.13	37.53 nM
	**Celecoxib**	-10.27	29.88 nM
**p38α (3GCP)**	**16g**	-10.69	14.51 nM
	**SB2**	-9.22	174.23 nM
**EGFR (1M17)**	**16g**	-9.52	104.58 nM
	**Erlotinib**	-7.39	3.18 μM
**CDK2 (2VTP)**	**16g**	-10.0	46.4 nM
	**LZ9**	-7.57	18.2 μM
**BRAF (4RZV)**	**16g**	-11.02	8.4 nM
	**Vemurafenib**	-12.77	432.93 pM
**VEGFR1 (3HNG)**	**16g**	-10.67	15.06 nM
	**8ST**	-12.06	1.45 nM

Validation of the docking study into COX-2

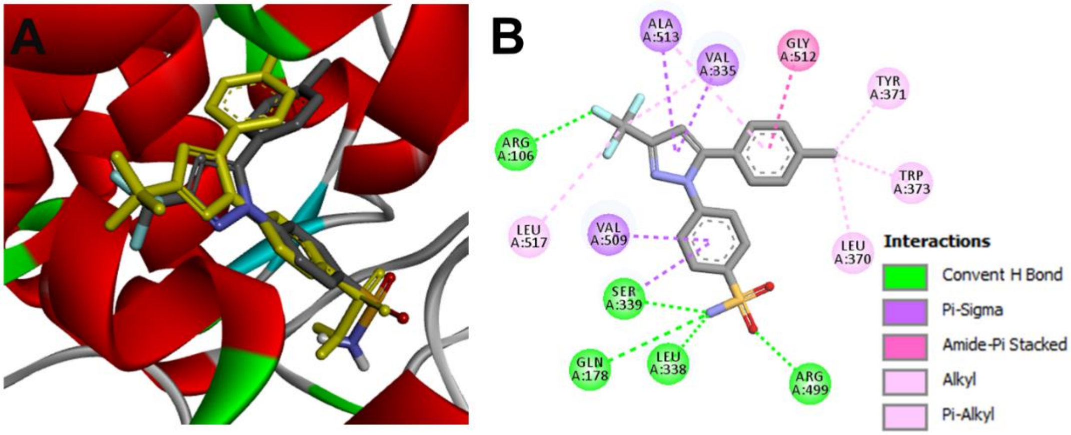

Figure S46. Validation of the docking study into COX-2 (pdb: 3LN1): A) 3D binding mode of re-docked celecoxib (shown as sticks colored by element) overlaid with the co-crystallized celecoxib (showed as yellow sticks) with RMSD of 0.82 Å; B) 2D binding mode of co-crystallized celecoxib showing different types of interactions with amino acids in COX-2.

Validation of the docking study into MAP P38α

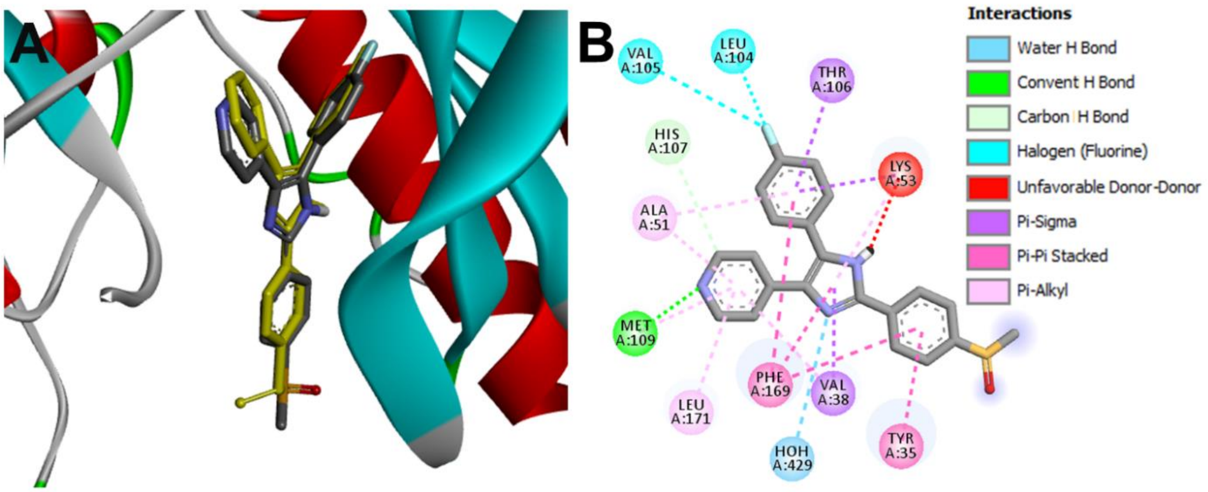

Figure S47. Validation of the docking study into MAP p38α (pdb: 3GCP): A) 3D binding mode of re-docked SB2 (shown as sticks colored by element) overlaid with the co-crystallized ligand, SB2 (showed as yellow sticks) with RMSD of 1.08 Å; B) 2D binding mode of co-crystallized SB2 showing different types of interactions with amino acids in p38.

Validation of the docking study into EGFR

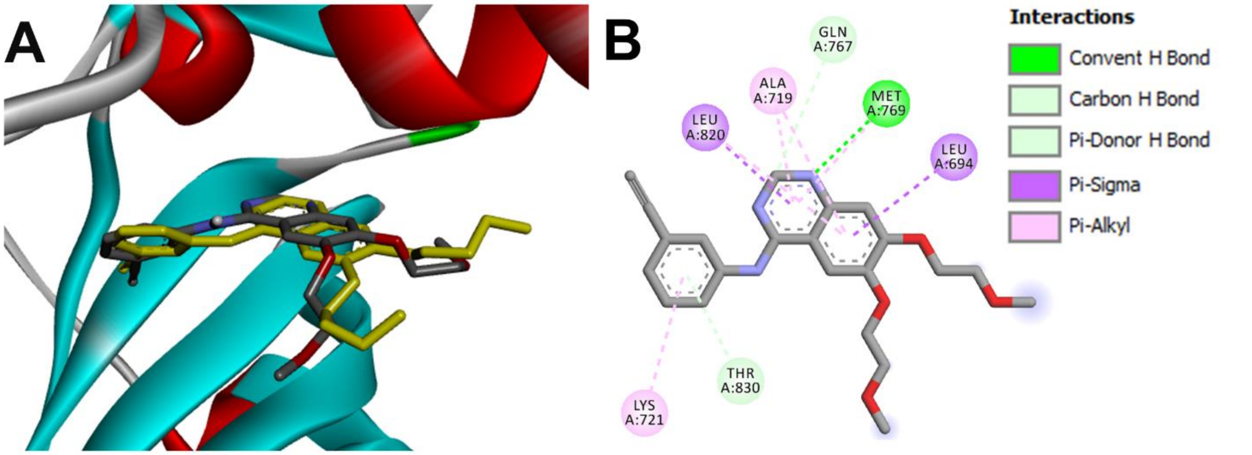

Figure S48. Validation of the docking study into EGFR (pdb: 1M17): A) 3D binding mode of re-docked erlotinib (shown as sticks colored by element) overlaid with the co-crystallized ligand, erlotinib (showed as yellow sticks) with RMSD of 1.44 Å; B) 2D binding mode of co-crystallized erlotinib showing different types of interactions with amino acids in EGFR.

Validation of the docking study into CDK2

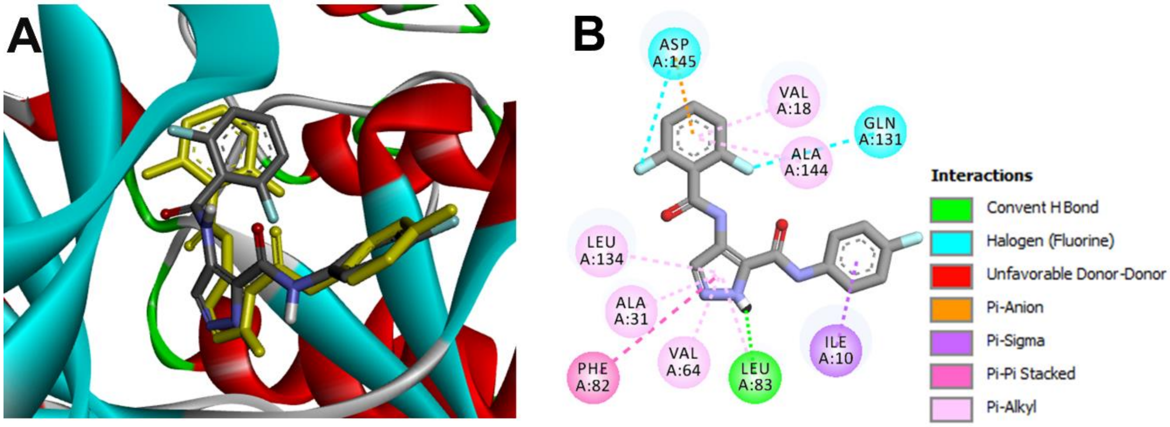

Figure 49. Validation of the docking study into CDK2 (pdb: 2VTP): A) 3D binding mode of re-docked LZ9 (shown as sticks colored by element) overlaid with the co-crystallized ligand, LZ9 (yellow sticks) with RMSD of 0.75 Å; B) 2D binding mode of LZ9 showing different types of interactions with amino acids CDK2.

Validation of the docking study into BRAF

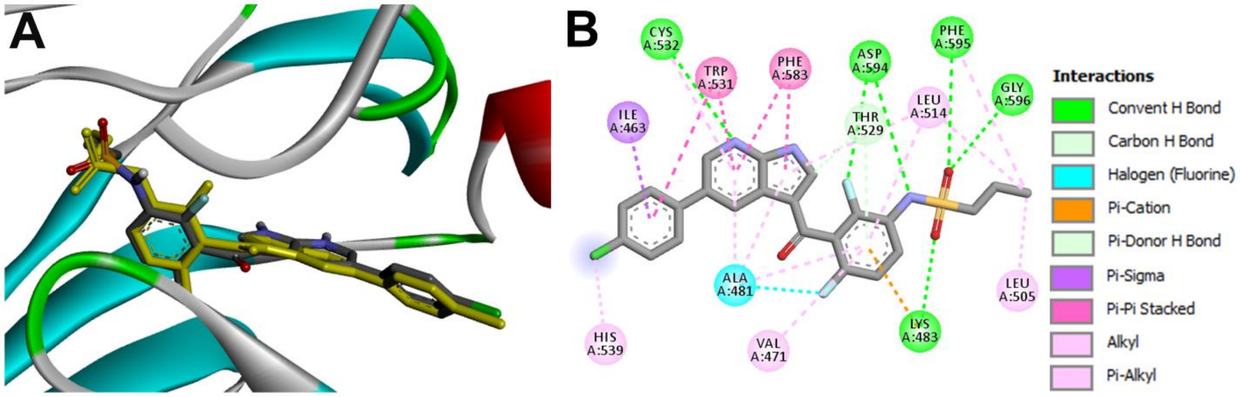

Figure 50. Validation of the docking study into BRAF (pdb: 4RZV): A) 3D binding mode of re-docked vemurafenib (shown as sticks colored by element) overlaid with the co-crystallized ligand, vemurafenib (yellow sticks) with RMSD of 0.58 Å ; B) 2D binding mode of vemurafenib showing different types of interactions with amino acids BRAF.

Validation of the docking study into VEGFR1

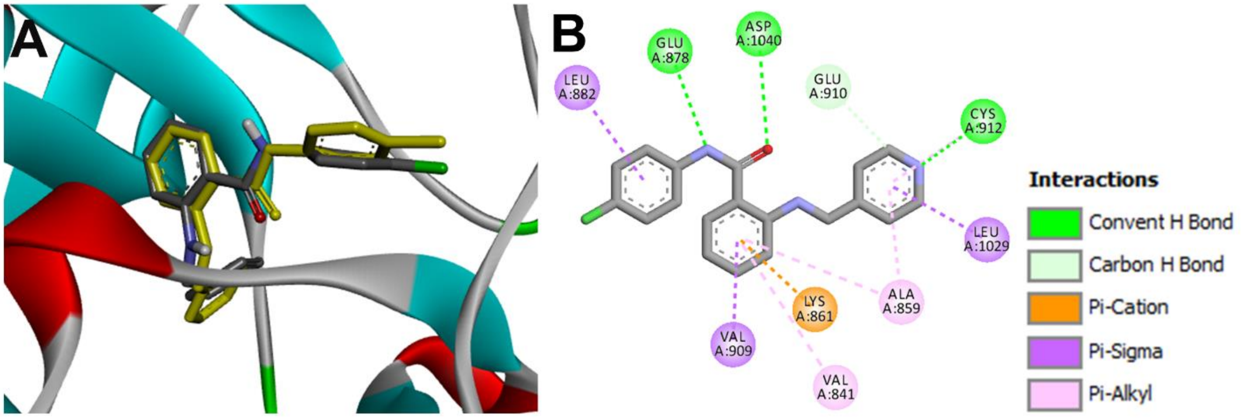

Figure 51. Validation of the docking study into VEGFR1 (pdb: 3HNG): A) 3D binding mode of re-docked 8ST (shown as sticks colored by element) overlaid with the co-crystallized ligand, 8ST (yellow sticks) with RMSD of 0.77 Å ; B) 2D binding mode of vemurafenib **s**howing different types of interactions with amino acids BRAF.

Figure 52. **Chemical structures of compound library**

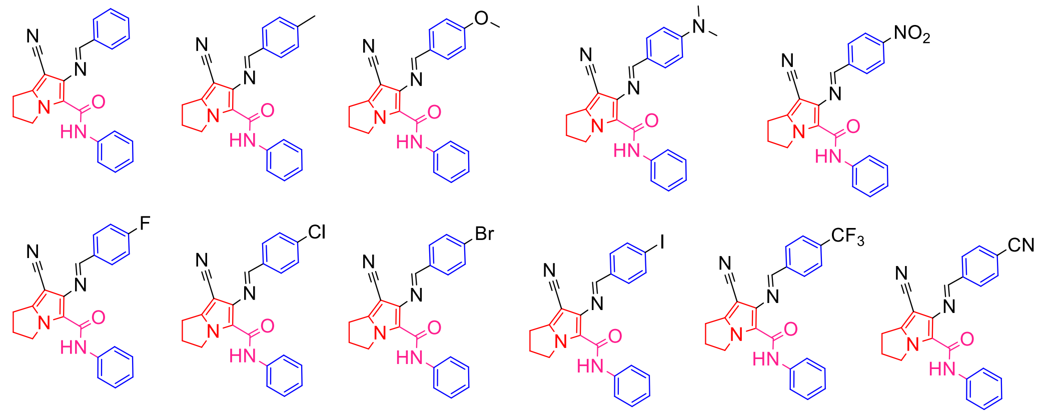

Figure 53. **Chemical structures of compound library (continued)**

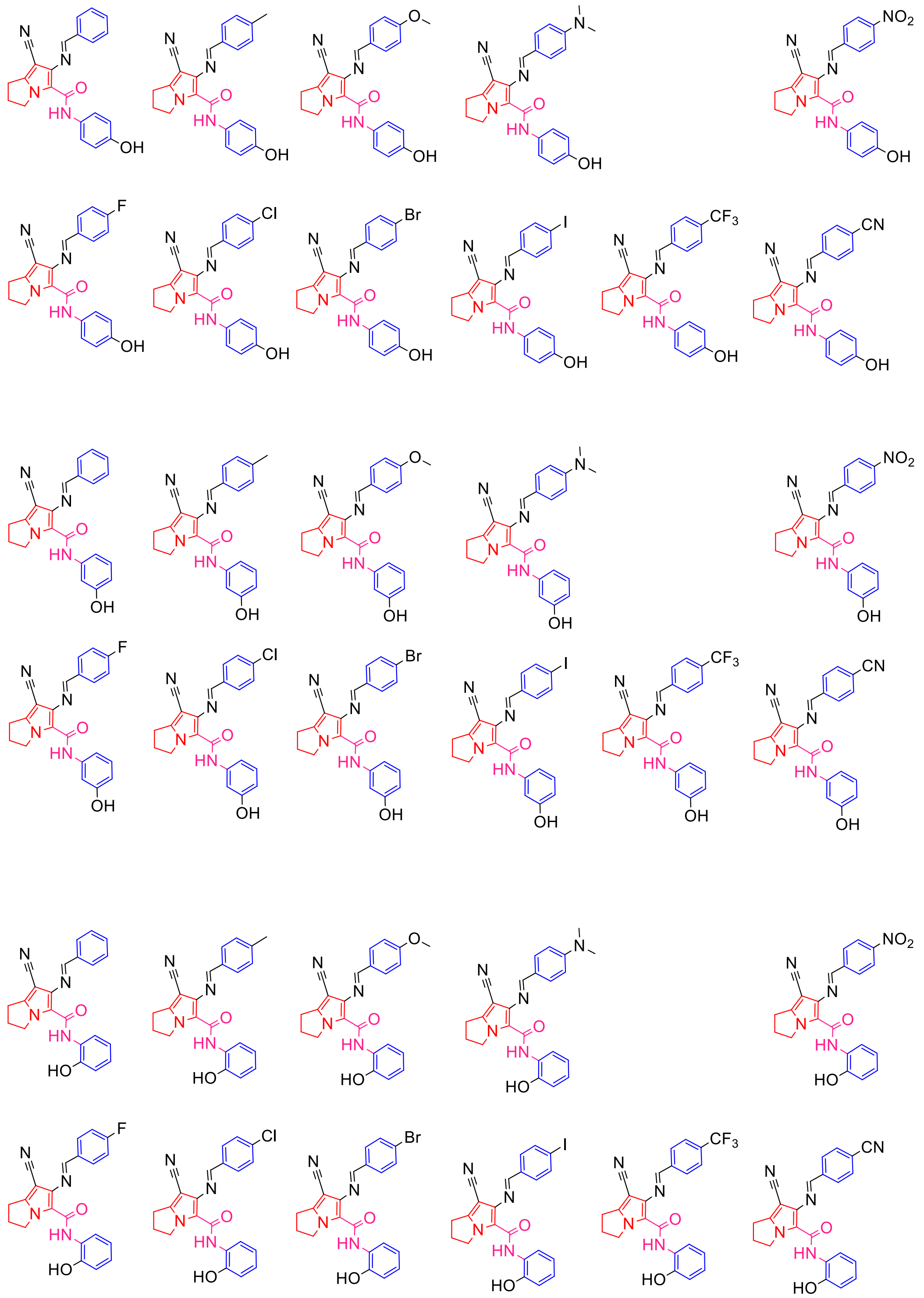

Figure 54. Chemical structures of compound library (continued)

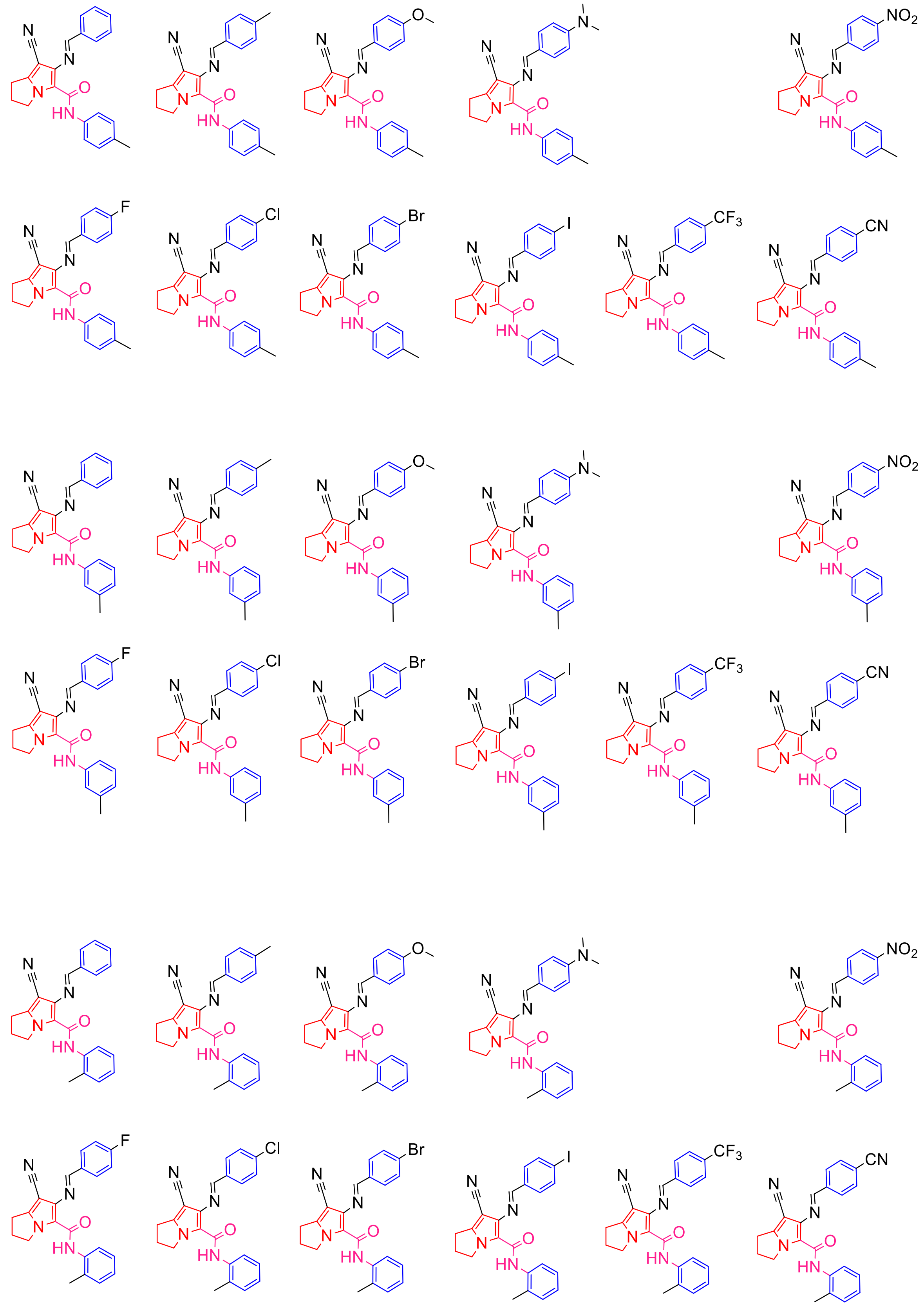

Figure 55. Chemical structures of compound library (continued)

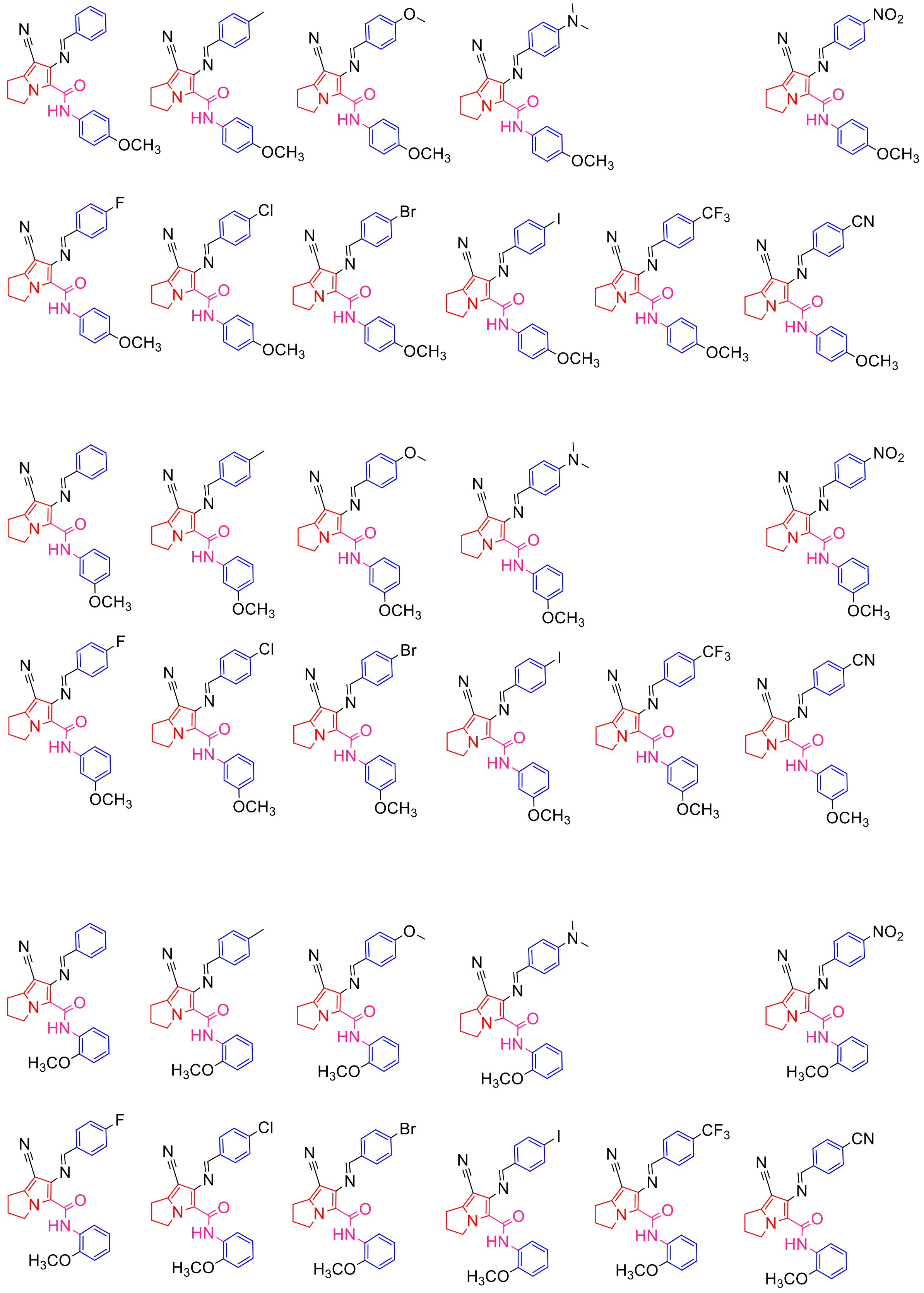

Figure 56. Chemical structures of compound library (continued)

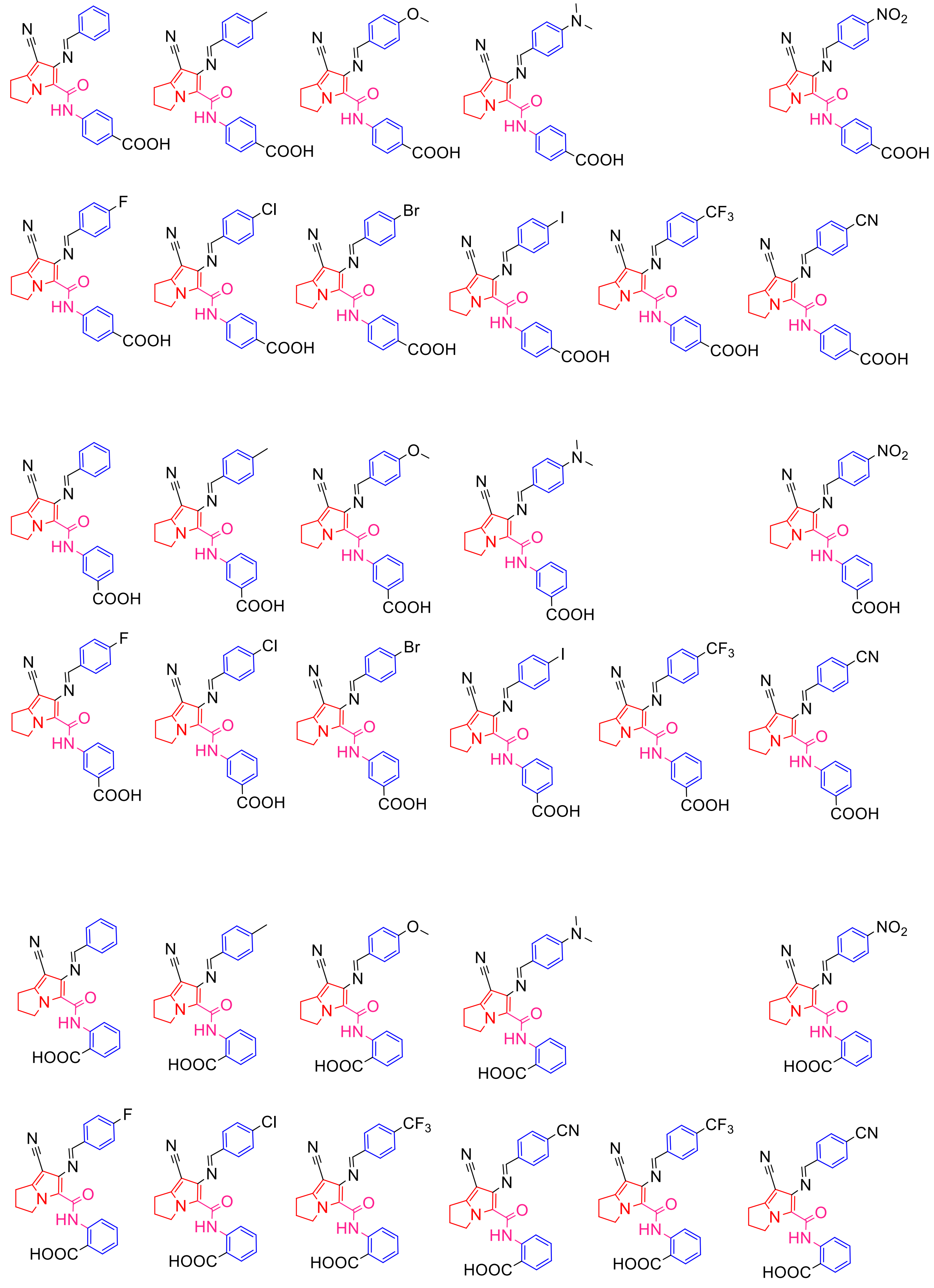

Figure 57. Chemical structures of compound library (continued)

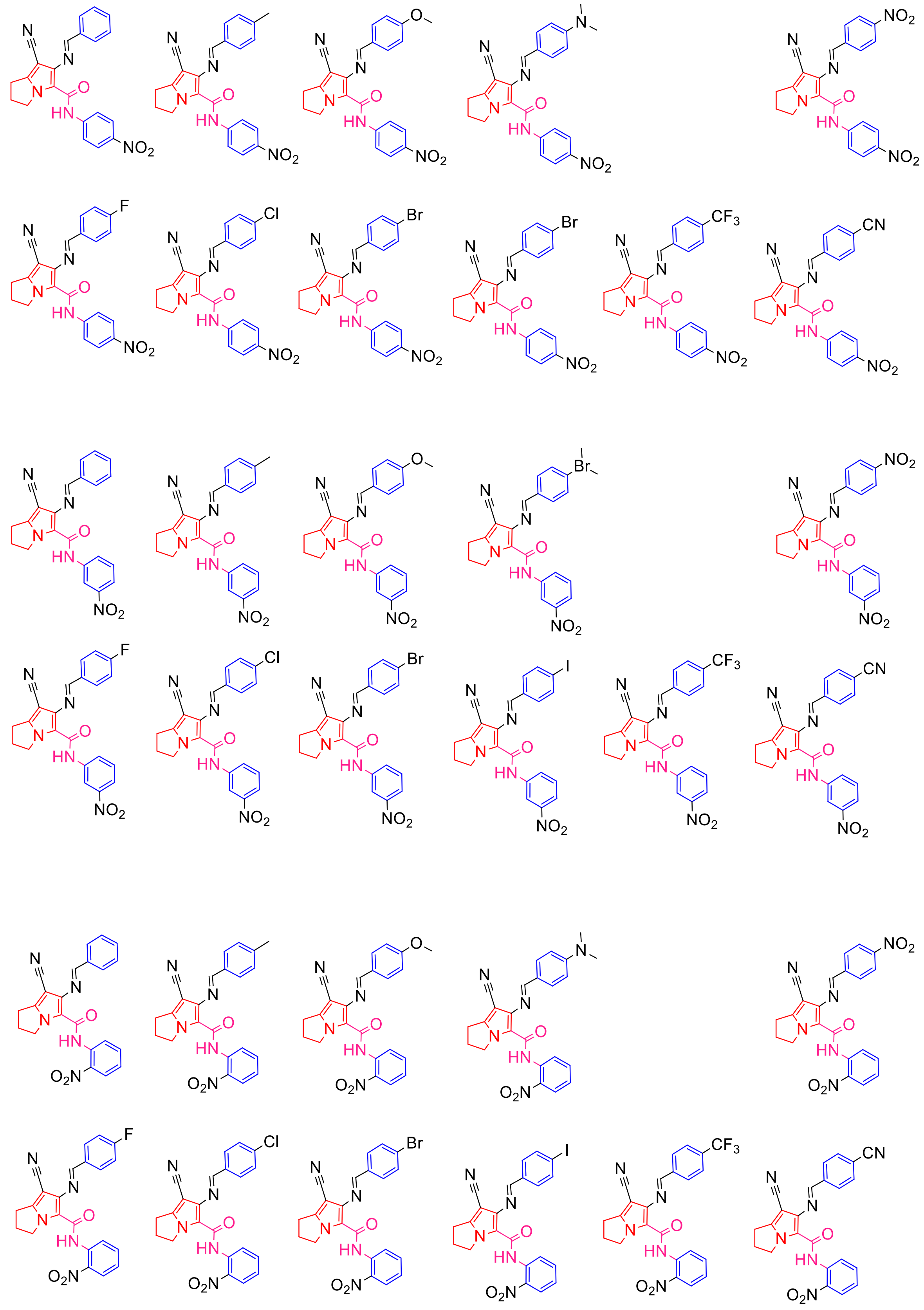

Figure 58. Chemical structures of compound library (continued)

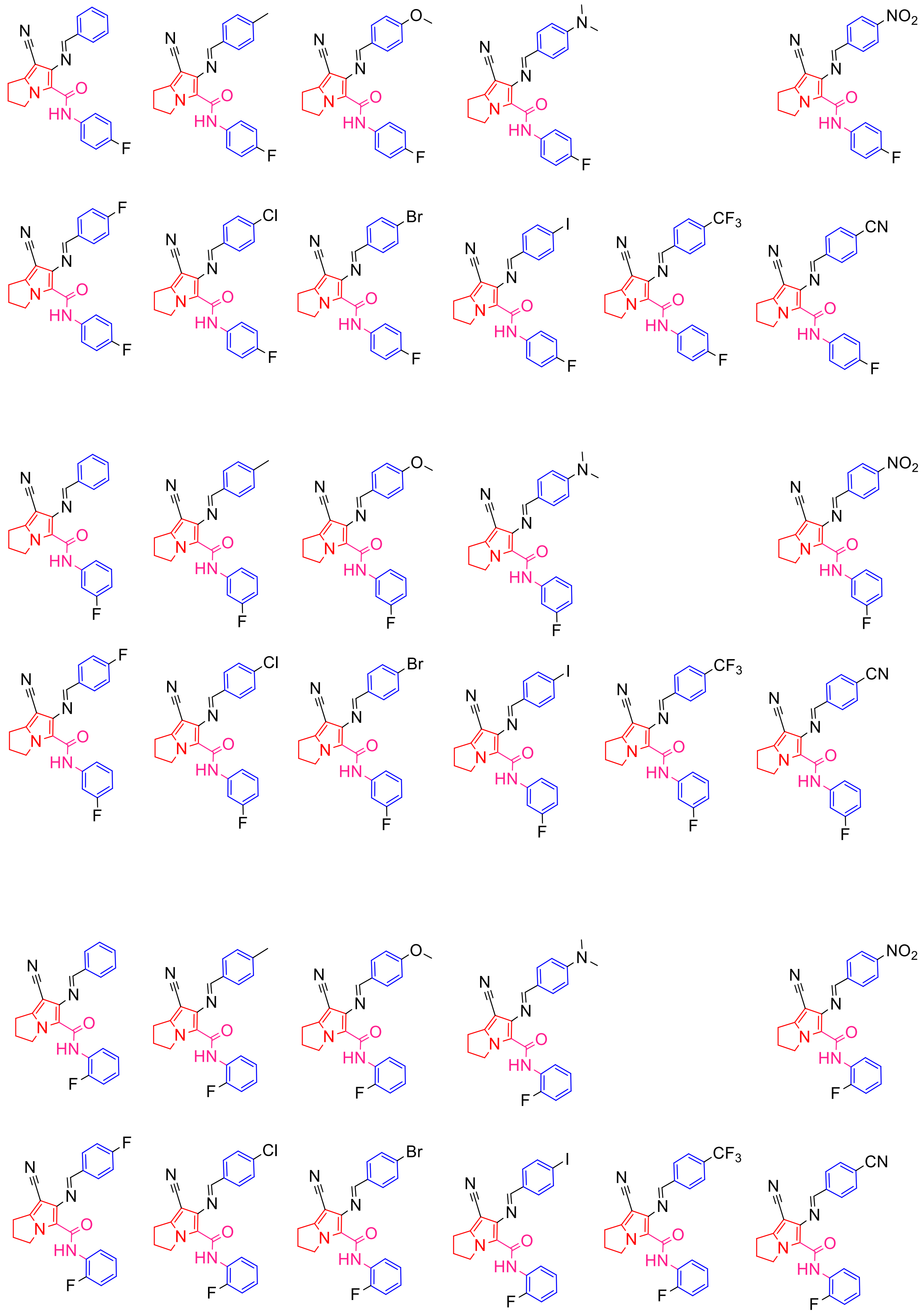

Figure 59. **Chemical structures of compound library (continued)**

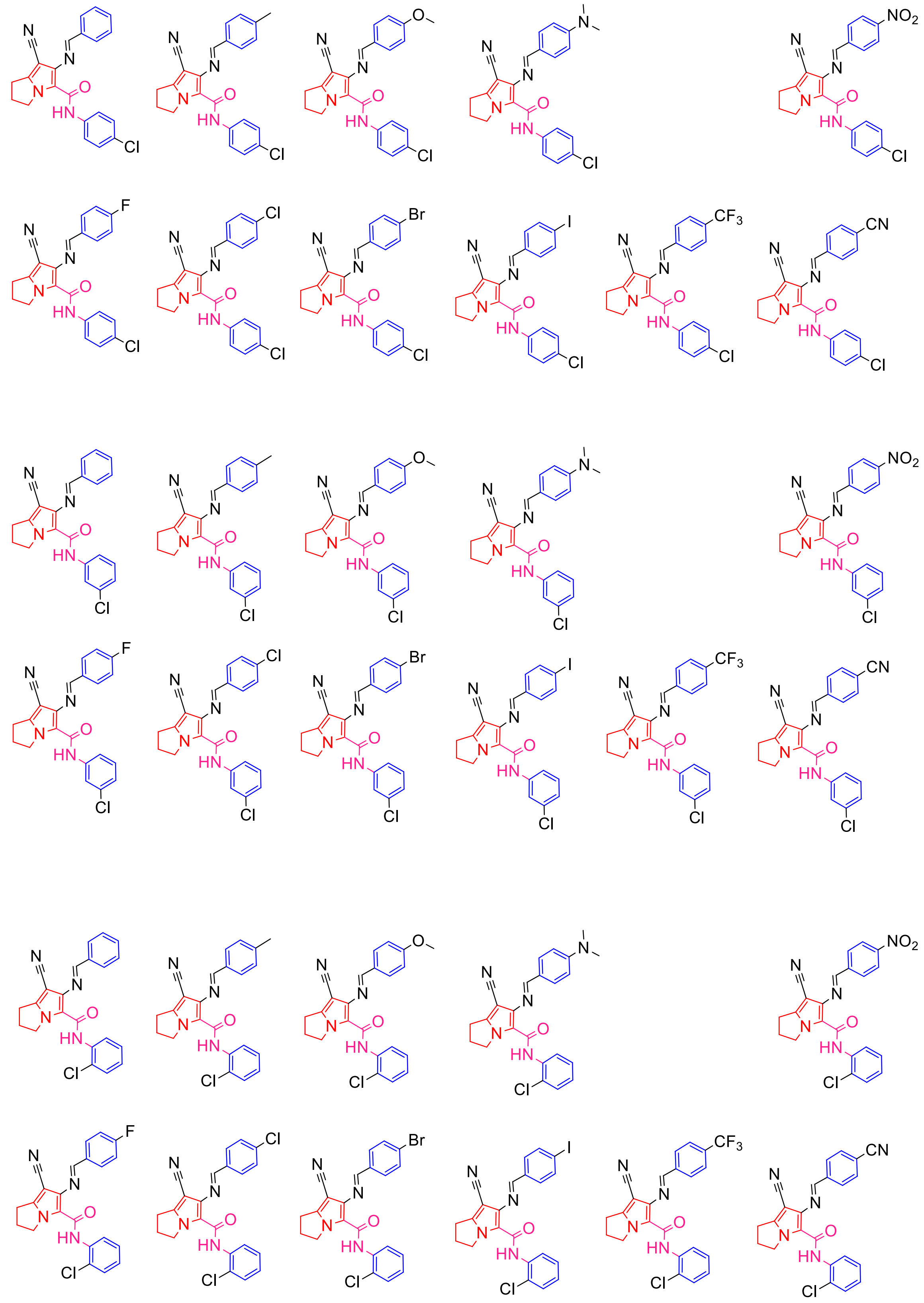

Figure 60. Chemical structures of compound library (continued)

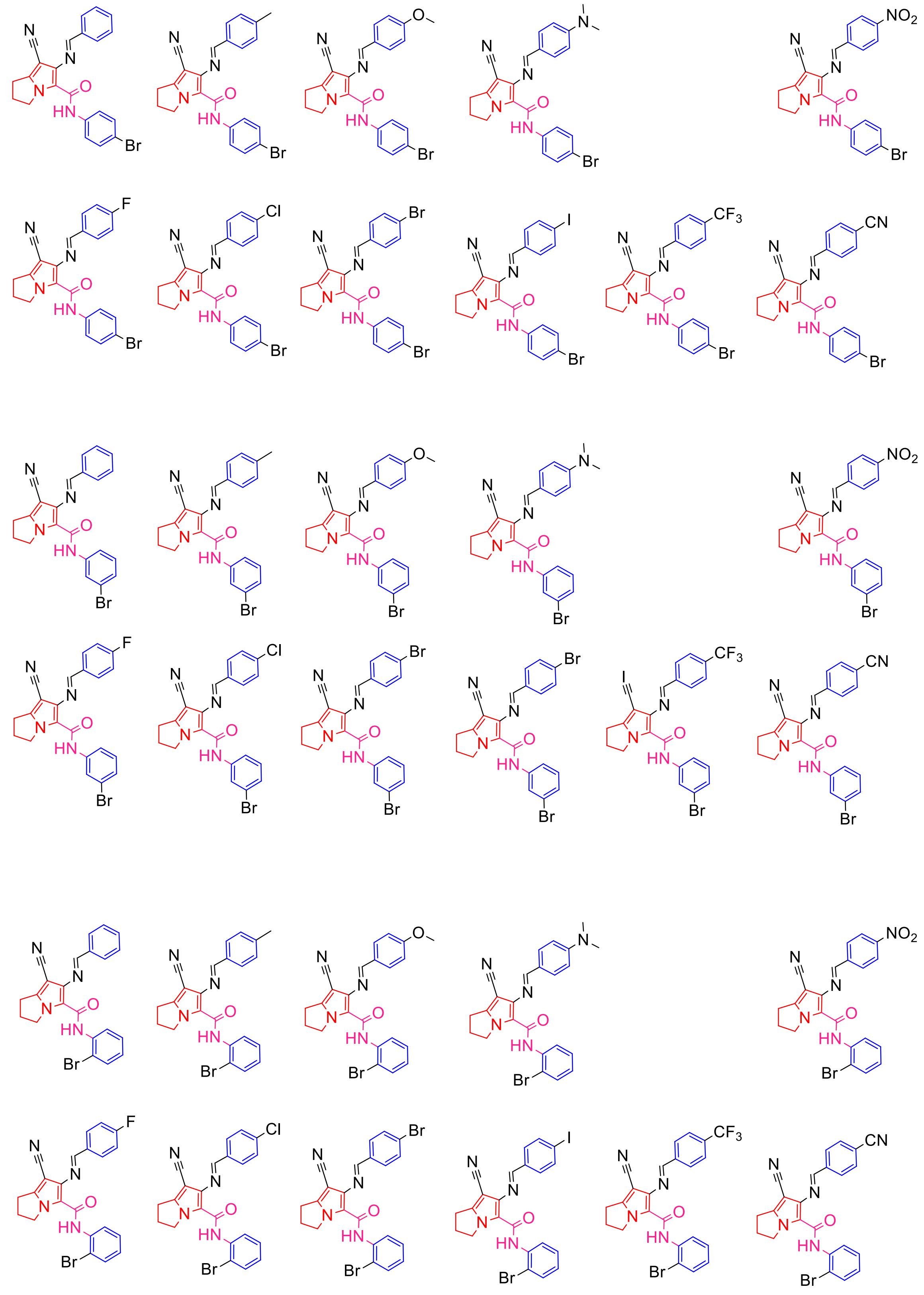

Figure 61. Chemical structures of compound library (continued)

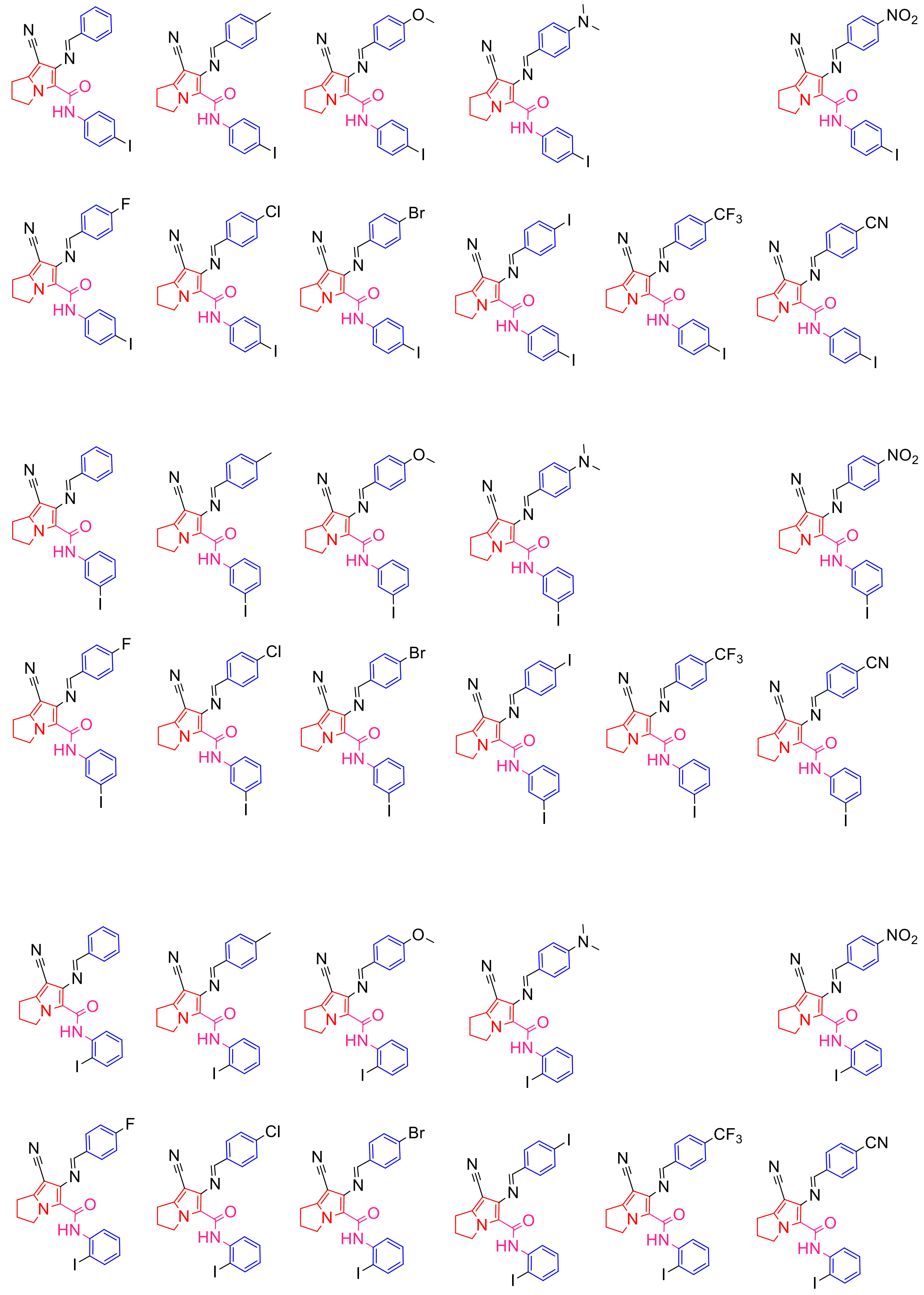

Supplement: Supplementary file 1 [file molecules-26-04002-s001.zip › molecules-1275201-supplementary.pdf]
